# Cross-metathesis reaction of α- and β-vinyl *C*-glycosides with alkenes

**DOI:** 10.3762/bjoc.11.150

**Published:** 2015-08-10

**Authors:** Ivan Šnajdr, Kamil Parkan, Filip Hessler, Martin Kotora

**Affiliations:** 1 Department of Organic Chemistry, Charles University in Prague, Hlavova 8, 153 00 Praha 2, Czech Republic, Fax: (+) 420 221 951 326; 2 Department of Chemistry of Natural Compounds, University of Chemistry and Technology, Prague, Technická 5, 160 00 Praha 6, Czech Republic

**Keywords:** *C*-glycosides, catalysis, carbohydrates, cross-metathesis, ruthenium

## Abstract

Cross-metathesis of α- and β-vinyl *C*-deoxyribosides and α-vinyl *C*-galactoside with various terminal alkenes under different conditions was studied. The cross-metathesis of the former proceeded with good yields of the corresponding products in ClCH_2_CH_2_Cl the latter required the presence of CuI in CH_2_Cl_2_ to achieve good yields of the products. A simple method for the preparation of α- and β-vinyl *C*-deoxyribosides was also developed. In addition, feasibility of deprotection and further transformations were briefly explored.

## Introduction

Natural and unnatural *C*-substituted glycosides are important compounds with a plethora of attractive biological properties and they often have been used as artificial DNA components [1]. Among various synthetic procedures providing *C*-deoxyribosides the one based on the use of a protected *C*-(2-deoxyribofuranosyl)ethyne, easily accessed by a coupling of a protected D-ribosyl halide and ethynylmagnesium chloride [2], offers a considerable synthetic flexibility since the triple bond could be transformed directly into various functional groups [3–13]. Thus the ethyne moiety was used in [2 + 2 + 2] cyclotrimerization to yield aryl *C*-deoxyribosides [3] and in a Sonogashira reaction for the synthesis of butenolidyl *C*-deoxyribosides [4]. Substituted alkynyl *C*-deoxyribosides [5,10–11] were used in other types of cycloaddition reactions providing indolyl *C*-deoxyribosides [6], cyclopentenonyl *C*-deoxyribosides [9], triazolyl *C*-deoxyribosides [12–13], carboranyl *C*-deoxyribosides [7], and finally also in Diels–Alder reaction with cyclobutadiene derivatives [8]. Despite of the above mentioned transformations, alkynyl *C*-deoxyribosides could also be used as a suitable starting material for hitherto rarely studied transformations.

One such a potential transformation is their hydrogenation to the corresponding vinyl *C*-deoxyribosides that could serve as intermediates for further functionalization. Interestingly, just a couple of reports regarding synthesis of vinyl *C*-deoxyribosides have been published so far. Among them is the Lindlar catalyst mediated hydrogenation of ethynyl β-*C*-deoxyriboside (prepared by a rather lengthy synthetic procedure) that provided vinyl β-*C*-deoxyribofuranosides [9]. Another procedure leading to pure vinyl β-*C*-deoxyribofuranoside was based on transformation of 6-*O*-*tert*-butyldiphenylsilyl-3,5-dideoxy-5-iodo-L-lyxo-hexofuranose [14]. A reaction sequence relying on Horner–Wadsworth–Emmons/ring closure–halogenation/Ramberg–Bäcklund/Wittig reaction gave rise to the equimolar mixture of styryl α- and β-*C*-deoxyribosides [15].

Finally, there is also a method utilizing an excess of vinylmagnesium bromide in the reaction with 3,5-bis-*O*-TBDPS-protected 2-deoxy-D-ribofuranose giving rise to a mixture of diastereoisomeric diols. The diasteroisomers were separated and cyclized in the presence of MsCl to the corresponding vinyl α-*C*-deoxyriboside α-**2** and β-*C*-deoxyriboside β-**2** [16]. As far as further transformation of vinyl *C*-deoxyribosides relying on the metathesis reaction is concerned, only one paper dealing with successful cross-metathesis with 4-vinyl-5-methyl-2-oxazolone has been reported [16]. This finding is rather surprising, because the metathesis reaction has been frequently used as a tool for chain elongation of various saccharides [17].

In view of the aforementioned, it is obvious that a development of a new and simple route to anomerically pure α- and β-vinyl *C*-deoxyribosides is desirable as well as to study the scope of their participation in cross-metathesis reactions. This procedure could thus open a new pathway for preparation of a number of alkenyl and alkyl *C*-deoxyriboside derivatives.

## Results and Discussion

### Synthesis of vinyl α- and β-*C*-deoxyribosides **2**

**Scheme 1:**
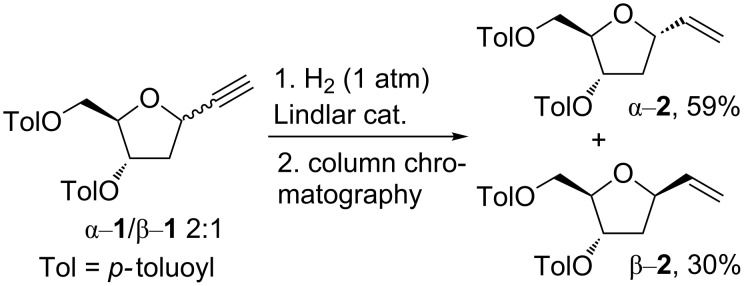
Synthesis of vinyl *C*-deoxyribosides α-**2** and β-**2**.

Although the simplest pathway for the preparation of vinyl α-*C*-deoxyriboside α-**2** and β-*C*-deoxyriboside β-**2** seems the reaction of a halogenose with ethynylmagnesium chloride followed by hydrogenation, this approach has not been reported yet (to the best of our knowledge). Presumably, difficulties regarding separation of a mixture of ethynyl α-*C*-deoxyriboside α-**1** and β-*C*-deoxyriboside β-**1** precluded any attempts. Notwithstanding this, we decided to test this approach. We found that hydrogenation of the epimeric mixture of ethynyl α-*C*-deoxyriboside α-**1** and β-*C*-deoxyriboside β-**1** on Lindlar catalyst at 1 atm of H_2_ provided, as expected, a mixture of the corresponding vinyl α-*C*-deoxyriboside α-**2** and vinyl β-*C*-deoxyriboside β-**2**. The mixture was easily separated into pure epimers (59% for α-**2** and 30% β-**2**) just by using a simple column chromatography (Scheme 1). Their identity was confirmed by comparison of the obtained spectral data with the published ones for related compounds [9,14,16]. This two-step reaction sequence is very simple and provides access to both epimers from a simply available starting material.

### Cross-metathesis reactions with vinyl α- and β-*C*-deoxyribosides **2**

**Figure 1:**
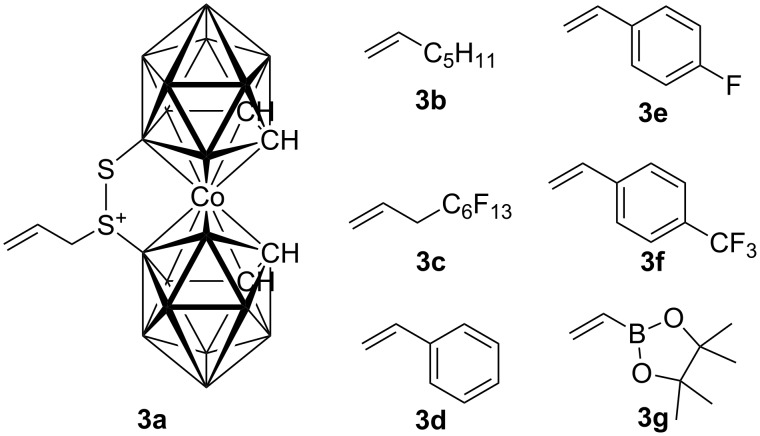
Alkenes **3** used in cross-metathesis reactions with **2**.

There is a general interest in synthesis of borylated [18] or carboranylated [8,19] saccharides and derivatives thereof because of their interesting properties. Bearing this in mind, we decided to explore the possibility of attaching the carborane moiety by using a cross-metathesis reaction. Cross-metathesis of α-**2** with the allylated carborane **3a** (Figure 1) was used as a model reaction [20–22]. Since it has been shown that the solvent [23] may profoundly affect the course of the cross-metathesis reaction in terms of activity and selectivity, we screened various reaction conditions to secure the highest yield of the desired cross-product of the reaction between α-**2** and **3a** (Table 1). The reactions were carried out in the presence of Hoveyda–Grubbs 2nd generation catalyst (HG II), which has been shown to be the best catalyst for cross-metathesis reactions [24]. Running the reaction under the standard conditions in dichloromethane or toluene under reflux, the desired cross-metathesis product α-**4a** was isolated in low 10% and 3% yields (Table 1, entries 1 and 2). Although it has been observed that the use of octafluorotoluene [23,25–27] as the solvent had a positive effect on yields, its use provided α-**4a** in a low 12% yield (Table 1, entry 3), but its use under microwave irradiation [26,28–30] gave rise to α-**4a** in 33% isolated yield (Table 1, entry 4). A similar result (36% yield) was obtained with a 1:1 octafluorotoluene/ClCH_2_CH_2_Cl mixture (Table 1, entry 5). Although microwave irradiation had a positive effect on the cross-metathesis reaction, see examples above, carrying out the reaction in a mixture of 1:1 octafluorotoluene/ClCH_2_CH_2_Cl under irradiation provided α-**4a** in only 3% (Table 1, entry 6). Finally, carrying out the reaction in pure ClCH_2_CH_2_Cl under reflux furnished the product in a nice 70% isolated yield (Table 1, entry 7), while microwave irradiation resulted in decreased yield of 58% (Table 1, entry 8). According to the obtained data in some cases microwave irradiation had a positive effect on the course of the reaction (Table 1, entry 4), whereas as in some cases it had a detrimental effect (Table 1, entries 6 and 8). Currently we do not know how to account for these observations; however, decomposition of the catalyst under these conditions cannot be excluded. In all of the above mentioned cases the unreacted starting material was recovered from the reaction mixtures.

**Table 1:** Conditions tested for cross-metathesis of α-**2** with **3a**.

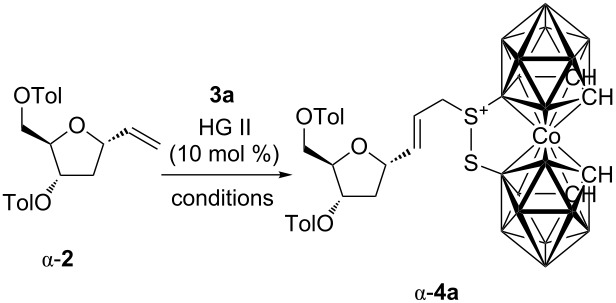		

Entry	Reaction conditions^a^	Yield (%)^b^

1	CH_2_Cl_2_, reflux, 24 h	10
2	toluene, reflux, 24 h	3
3	C_6_F_5_CF_3_, reflux, 16 h	12
4	C_6_F_5_CF_3_, mw^c^	33
5	C_6_F_5_CF_3_/ClCH_2_CH_2_Cl 1:1, 16 h	36
6	C_6_F_5_CF_3_/ClCH_2_CH_2_Cl 1:1, mw^c^	3
7	ClCH_2_CH_2_Cl, reflux, 12 h	70
8	ClCH_2_CH_2_Cl, mw, 110 °C, 2 h^c^	58

^a^α-**2** (0.26 mmol), solvent (5 mL). ^b^Isolated yields. ^c^mw = microwave irradiation.

With these results in hand we decided to screen the scope of cross-metathesis reactions with other terminal alkenes **3b**–**3g** (Figure 1, Table 2). Our first choice was 1-heptene (**3b**), which reacted under the above mentioned conditions (i.e., with HG II in ClCH_2_CH_2_Cl under reflux) to give the corresponding product α-**4b** in 59% isolated yield (Table 2, entry 2). We also carried out the reaction with perfluorohexylpropene (**3c**), because of our long term interest in the synthesis of perfluoroalkylated compounds [21,30–34] and their application [35]. The reaction furnished the desired compound α-**4c** in a good 50% isolated yield (Table 2, entry 3). Next we switched our attention to styrenes **3d**–**3f**. In all cases the corresponding products α-**4d**–α-**4f** were obtained in good 68, 60, and 59% isolated yields, respectively (Table 2, entries 4–6). Finally, cross-metathesis with vinylboronic acid pinacol ester (**3g**) was attempted. Once again the reaction proceeded well, furnishing boronate α-**4g** in 66% isolated yield (Table 2, entry 7). Then we turned to reactions of the above mentioned terminal alkenes with β-**2**. In all cases the corresponding products were obtained in good isolated yields in the range similar to α-**2**. The metathesis with the allylated carborane **3a** provided β-**4a** in 77% yield (Table 2, entry 8). The reaction with 1-heptene (**3b**) and perfluorohexylpropene (**3c**) gave the corresponding products β-**4b** and β-**4c** in 64 and 48% yields (Table 2, entries 9 and 10). In a similar manner also the styrenes **3d**–**3f** furnished the desired products β-**4d**–β-**4f** in 69, 58, and 61% yields, respectively (Table 2, entries 11–13). Similarly compound **3g** reacted well providing the boronate β-**4g** in a nice 64% yield (Table 2, entry 14). The latter boronate was subjected to coupling with iodobenzene under Suzuki conditions and the corresponding product β-**4d** was obtained in 51% isolated yield.

**Table 2:** Cross-coupling of **2α** and β-**2** with alkenes **3**.

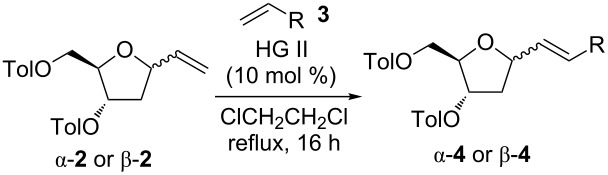				

Entry	**2**	**3**	**4**	Yield (%)^a^

1	α-**2**	**3a**	α-**4a**	74
2		**3b**	α-**4b**	59
3		**3c**	α-**4c**	50
4		**3d**	α-**4d**	68
5		**3e**	α-**4e**	60
6		**3f**	α-**4f**	59
7		**3g**	α-**4g**	66
8	β-**2**	**3a**	β-**4a**	77
9		**3b**	β-**4b**	64
10		**3c**	β-**4c**	48
11		**3d**	β-**4d**	69
12		**3e**	β-**4e**	58
13		**3f**	β-**4f**	61
14		**3g**	β-**4g**	64

^a^Isolated yields.

**Scheme 2:**
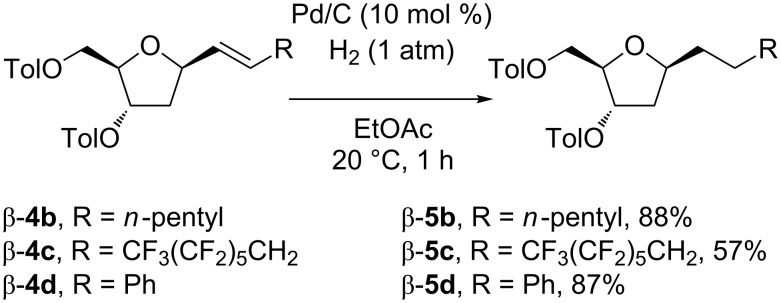
Hydrogenation of β-**4b**–β-**4d** to β-**5b**–β-**5d**.

**Scheme 3:**
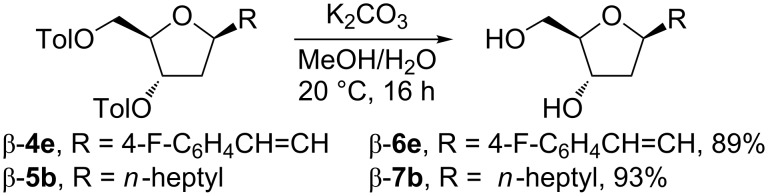
Deprotection of β-**4e** and β-**5b** to β-**6e** and β-**7b**.

With the *C*-deoxyribosides on hand, the feasibility of catalytic hydrogenation was also briefly explored. Compounds possessing the heptenyl side chain (β-**4b**), tridecafluorononenyl side chain (β-**4c**), and the styryl side chain (β-**4d**) were chosen as substrates. In all cases the hydrogenation by using Pd/C under low pressure of hydrogen (1 atm) proceeded uneventfully to give rise to products with the saturated side chain β-**5b**, β-**5c**, and β-**5d** in good isolated yields of 88, 57, and 87% (Scheme 2). In addition, deprotection of the toluoyl groups was tested on compounds bearing an unsaturated side chain such as β-**4e** and a saturated side chain such as β-**5b** by using K_2_CO_3_ in a mixture of MeOH/H_2_O. In both cases the reaction proceeded almost quantitatively providing the corresponding *C*-deoxyribosides β-**6e** and β-**7b** in 89 and 93% isolated yields (Scheme 3).

### Cross-metathesis reactions with 1-(tetra-*O*-acetyl-α-D-galactopyranosyl)ethene (**8**)

There have been, to the best of our knowledge, just a handful of reports of cross-metathesis reactions of other vinyl *C*-glycosides. Among these reports metatheses of 1-(D-glucopyranosyl)prop-2-ene derivatives with various alkenes [36–40] and one report regarding a 1-(α-D-galactopyranosyl)ethene derivative with allyl amines [41]. Because of our interest in the synthesis of various D-galactose derivatives, we decided to explore the scope of their metathesis reaction with several different alkenes.

The starting material – 1-(tetra-*O*-acetyl-α-D-galactopyranosyl)ethene (**8**) – was prepared according to the previously reported procedure. A solution of penta-*O*-acetyl-D-galactose, allyltrimethylsilane and BF_3_·Et_2_O was refluxed in acetonitrile giving a 6:1 mixture of α- and β-epimers of 1-(tetra-*O*-acetyl-D-galactopyranosyl)prop-2-ene in 98% yield. Zemplén deacetylation afforded quantitatively the same mixture of epimeric 1-(D-galactopyranosyl)prop-2-enes that were dissolved in ethanol and treated with ether. This allowed the α-epimer to precipitate and it could afterwards be isolated as a pure crystalline product in 60% yield [42]. Its acetylation afforded 1-(tetra-*O*-acetyl-α-D-galactopyranosyl)prop-2-ene in high yield and purity. It was then isomerized [43] to 1-(tetra-*O*-acetyl-α-D-galactopyranosyl)prop-1-ene (80% yield) that was subjected to cross-metathesis with ethene to give the desired compound **8** in 82% yield [41].

The above mentioned metathesis conditions – HG II, reflux in 1,2-dichloroethane – were also tested in the reactions of **8** with alkenes **3d**–**3f** (Table 3). However, the yields of the corresponding products **9d**–**9f** were around 60% (Table 3, entries 1–3, column IV). Switching the solvent to dichloromethane did not have any substantial effect on the yields of the corresponding products (57–70%) (Table 3, entries 1–3, column V). Moreover, in all above mentioned cases the starting material remained partially unreacted and could not be easily separated from the desired products.

**Table 3:** Cross-metathesis of **8** with alkenes **3**.

Entry	**3**	**9**	Yield (%)^a^ (in ClCH_2_CH_2_Cl)	Yield (%)^a^ (in CH_2_Cl_2_)

1	**3d**	**9d**	58	70
2	**3e**	**9e**	61	57
3	**3f**	**9f**	56	58

^a^Isolated yield.

A considerable improvement was observed when the metatheses were run in dichloromethane and in the presence of CuI (Table 4) [44]. In all cases the reactions provided the corresponding products in very good isolated yields. The first metatheses were carried out with 1-heptene (**3b**) and perfluorohexylpropene (**3c**) furnishing **9b** and **9c** in nice 80 and 79% isolated yields, respectively (Table 4, entries 1 and 2). Then we switched our attention to styrenes **3d**–**3f**. In all cases the corresponding products **9d**–**9f** were obtained in good 82, 79, and 78% isolated yields, respectively (Table 4, entries 3–5). In addition, in all cases deprotection under basic conditions provided the corresponding *C*-alkenylated D-galactoses in very good isolated yields (86–93%).

**Table 4:** Cross-metathesis of **8** with alkenes **3** in the presence of CuI.

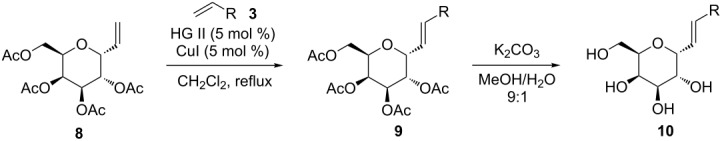					

Entry	**3**	**9**	Yield (%)^a^	**10**	Yield (%)^a^

1	**3b**	**9b**	80	**10b**	88
2	**3c**	**9c**	79	**10c**	80
3	**3d**	**9d**	82	**10d**	93
4	**3e**	**9e**	79	**10e**	90
5	**3f**	**9f**	78	**10f**	87

^a^Isolated yield.

## Conclusion

In conclusion, the cross-metathesis reaction of anomerically pure vinyl *C*-deoxyriboses (easily accessible from a mixture of ethynyl α/β-*C*-deoxyribosides) with alkenes bearing various functional groups proceeded in the presence of a catalytic amount of HG II catalysts in refluxing 1,2-dichloroethane giving rise to the corresponding alkenylated derivatives in good yields and without loss of stereochemical information. Deprotection as well as hydrogenation is also feasible providing the desired compounds as exemplified in selected examples. In addition, this methodology is also applicable to vinyl α-*C*-D-galactopyranoside, albeit the best results were obtained when the reaction was carried out in refluxing dichloromethane and in the presence of CuI. Deprotection of the prepared alkenylated derivatives proceeded without any problems.

Since homodimerization of the starting alkenes **2** and **8** has not been observed under the reaction conditions used (however, we cannot exclude that minor undetected amounts of homodimers of **2** or **8** were formed), they could be preliminarily considered as type II or III olefins according to the Grubbs classification of olefins [45].

## Supporting Information

File 1 Detailed experimental procedures for all compounds, characterization of the synthesized compounds, and copies of ^1^H/^13^C NMR spectra for all compounds.
