# Supplementary material for: Cross-metathesis reaction of α- and β-vinyl C-glycosides with alkenes
Source: Beilstein J Org Chem. 2015 Aug 10;11:1392–7. doi: 10.3762/bjoc.11.150 (PMC4578437; doi:10.3762/bjoc.11.150)
Supplement: File 1 — Detailed experimental procedures for all compounds, characterization of the synthesized compounds, and copies of 1H/13C NMR spectra for all compounds. [file Beilstein_J_Org_Chem-11-1392-s001.pdf]

Supporting Information

for

Cross-metathesis reaction of α- and β-vinyl *C*-glycosides with alkenes

Ivan Šnajdr[1], Kamil Parkan[2], Filip Hessler[1] and Martin Kotora[1]*

Address: [1]Department of Organic Chemistry, Charles University in Prague, Hlavova 8, 153 00 Praha 2, Czech Republic, Fax: (+) 420 221 951 326 and [2]Department of Chemistry of Natural Compounds, University of Chemistry and Technology, Prague, Technická 5, 160 00 Praha 6, Czech Republic

Email: Martin Kotora - martin.kotora@natur.cuni.cz

*Corresponding author

Detailed experimental procedures for all compounds, characterization of the synthesized compounds, and copies of $^{1}H/^{13}C$ NMR spectra for all compounds

Contents

I General S2

II Preparation of α-**2** and β-**2** S3

III Cross-metathesis of α-**2** and β-**2** S5

IV Catalytic reduction of the β-**4b**– β-**4d** S13

V Deprotection of **4** and **5** S15

VI Cross-metathesis of **8** S16

VII Deprotection of **9** S19

VIII Copies of spectra S22

I General.

All solvents were used as obtained unless otherwise noted. Dichloroethane was distilled and dried over 4 Å molecular sieves. All other reagents were obtained from commercial sources. All metathesis reactions were carried out under an argon atmosphere using Schlenk-tube technique. The NMR spectra were measured on Varian Mercury 300, Varian Mercury 400 and Bruker AVANCE 600 instruments (^{1}H at 300 or 600 MHz; ^{13}C at 75 or 151 MHz, ^{19}F at 282 MHz and ^{11}B NMR at 128 MHz) as solutions in $CDCl_3$ or MeOD at 20 °C unless otherwise noted. Chemical shifts are given in δ scale (^{1}H NMR spectra were referenced to TMS as an internal standard, ^{13}C NMR spectra to $CDCl_3$ at δ 77.0, ^{19}F NMR spectra were referenced to C_6F_6 at δ −163.0 and ^{11}B NMR to $BF_3{\cdot}Et_2O$ as an internal standard), coupling constants *J* are given in Hz. Melting points (uncorrected) were determined using a Kofler apparatus. Infrared spectra were recorded as $CHCl_3$ solutions or as KBr tablets and are reported in wave numbers (cm^{-1}). Fluka 60 silica gel was used for flash chromatography. TLC was performed on silica gel 60 F_{254}-coated aluminum.

II Synthesis of the starting compounds.

Synthesis of 1-(3,5-bis-*O*-(4-toluoyl)-2-deoxy-α-D-ribofuranosyl)ethene (α-2) and 1-(3,5-bis-*O*-(4-toluoyl)-2-deoxy-β-D-ribofuranosyl)ethene (β-2). Into a solution of 2:1 anomeric mixture of 1,2-dideoxy-3,5-di-*O*-(4-toluoyl)-D-ribofuranosyl)eth-1-yne (2 mmol, 0.76 g) in a 20:1 mixture of ethyl acetate/pyridine (21 mL) Lindlar catalyst (0.2 mmol, 42 mg) was added and the flask was filled with hydrogen atmosphere. The reaction mixture was then stirred at 20 °C for 4 h. Afterwards, all volatiles were removed under reduced pressure and column chromatography of the residue on silica gel (15:1 hexanes/EtOAc) furnished 0.45 g (59%) of **α-2** and 0.22 g (30 %) of **β-2**.

α-2: a colorless oil; R_f = 0.60 (4/1 hexanes/EtOAc); $[\alpha]_D$ = 32.3° (c = 0.0155 g/ml, $CHCl_3$); ^1H NMR (300 MHz, $CDCl_3$) δ 8.02–7.82 (m, 4H, H-Tol), 7.33–7.15 (m, 4H, H-Tol), 6.01 (ddd, *J* = 17.0, 10.3, 6.6 Hz, 1H, H-1), 5.52 (dt, *J* = 6.8, 3.3 Hz, 1H, H-3´), 5.31 (dt, *J* = 17.2, 1.4 Hz, 1H, H-2a), 5.18 (dt, *J* = 10.3, 1.4 Hz, 1H, H-2b), 4.83–4.66 (m, 1H, H-1´), 4.61–4.40 (m, 3H, H-4´, H-5´), 2.70 (dt, *J* = 14.2, 7.3 Hz, 1H, H-2´a), 2.42 (s, 3H, CH_3-Tol), 2.40 (s, 3H, CH_3-Tol), 2.08 (ddd, *J* = 13.6, 5.4, 3.7 Hz, 1H, H-2´b); ^{13}C NMR (75 MHz, $CDCl_3$) δ 166.36 (C=O), 166.16 (C=O), 144.03 (C-Tol), 143.79 (C-Tol), 138.46 (C-1), 129.71 (C-Tol), 129.12 (C-Tol), 127.10 (C-Tol), 126.96 (C-Tol), 116.05 (C-2), 81.63 (C-4), 80.02 (C-1), 76.49 (C-3), 64.70 (C-5), 38.20 (C-2), 21.69 (CH_3-Tol); IR (KBr, cm^{-1}) ν 1721, 1613, 1272, 1177, 1108, 1018, 755; MS (ES+, m/z (rel.%)) 231.1 (10), 136.1 (30), 119.0 (100), 91.1 (50), 65.0 (10); HRMS (ESI) calcd. for $C_{23}H_{24}O_5$: 403.1516, found 403.1517.

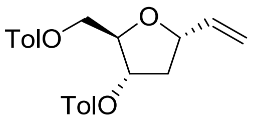

β-2: a colorless oil; R_f = 0.64 (4/1 hexanes/EtOAc); $[\alpha]_D$ = 28.4° (c = 0.0109 g/ml, $CHCl_3$); ^1H NMR (300 MHz, $CDCl_3$) δ 8.00–7.87 (m, 4H, H-Tol), 7.31–7.17 (m, 4H, H-Tol), 5.89 (ddd, *J* = 17.1, 10.3, 6.6 Hz, 1H, H-1), 5.51 (dt, *J* = 6.2, 1.5 Hz, 1H, H-3´), 5.37 (d, *J* = 17.2 Hz, 1H, H-2a), 5.19 (d, *J* = 10.3 Hz, 1H, H-2b), 4.68 (dt, *J* = 11.2, 5.8 Hz, 1H, H-1´), 4.52 (d, *J* = 4.5 Hz, 2H, H-5´), 4.42 (td, *J* = 4.4, 2.0 Hz, 1H, H-4´), 2.42 (s, 3H, CH_3-Tol), 2.40 (s, 3H, CH_3-Tol), 2.32 (ddd, *J* = 13.7, 5.2, 1.3 Hz, 1H, H-2´a), 2.09 (ddd, *J* = 13.8, 10.6, 6.0 Hz, 1H, H-2´b); ^{13}C NMR (75 MHz, $CDCl_3$) δ 166.32 (C=O), 166.08 (C=O), 144.06 (C-Tol), 143.75 (C-Tol), 137.34 (C-1), 129.70 (C-Tol), 129.16 (C-Tol), 129.13 (C-Tol), 127.02 (C-Tol), 117.12 (C-2), 82.58 (C-4), 80.24 (C-1), 77.12 (C-3), 64.74 (C-5), 39.08 (C-2), 21.68 (CH_3-Tol); IR (KBr, cm^{-1}) ν 1721, 1610, 1266, 1180, 1111, 1018, 758; MS (ES+, m/z (rel.%)) 244.1 (10), 231.1 (10), 136.1 (30), 119.0 (100), 108.1 (20), 91.1 (30); HRMS (ESI) calcd. for $C_{23}H_{24}O_5$: 403.1516, found 403.1515.

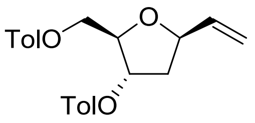

1-(2´,3´,4´,6´-Tetra-*O*-acetyl-α-D-galactopyranosyl)ethene (8). A solution of **1-(tetra-*O*-acetyl-α-D-galactopyranosyl)prop-1-ene** [1] (5.37 mmol, 2 g) in dry CH_2Cl_2 (55 mL) was placed in a pressure reactor. Grubbs' 2nd generation catalyst (1.07 mmol, 912 mg, in two portions with 24 h interval) was added to the solution and the reactor was purged with vacuum. Afterwards ethylene was charged up to 10 bar and the reaction mixture was refluxed for 3 days. All volatiles were removed in vacuo and column chromatography of the residue on silica gel (4:1 hexane/EtOAc) afforded the title compound **8** (1.58 g, 82%) as white crystals. Spectral characteristics were in agreement with the previously reported data.[2] R_f = 0.2 (3/1 hexane/EtOAc); m.p. 98-99°C (ethyl acetate/heptane); R_f = 0.4 (3/1 hexane/EtOAc); $[\alpha]_D$ = 135.2° (*c* = 0.451 g/ml, $CHCl_3$); ^{1}H NMR (600 MHz, $CDCl_3$) δ 5.96 (ddd, *J* = 17.7, 10.6, 4.9 Hz, 1H, H-1), 5.49 – 5.45 (m, 2H; H-2a, H-2b), 5.41 (dd, *J* = 3.3, 1.8 Hz, 1H, H-4´), 5.34 (dd, *J* = 10.5, 6.0 Hz, 1H, H-2´), 5.13 (dd, *J* = 10.5, 3.3 Hz, 1H, H-3´), 4.79 (m, 1H, H-1´), 4.19 (dt, *J* = 6.4, 1.7 Hz, 1H, H-5´), 4.14 (dd, *J* = 11.3, 6.9 Hz, 1H, H-6a´), 4.08 (dd, *J* = 11.3, 5.9 Hz, 1H, H-6b´), 2.15 (s, 3H, CH_3CO), 2.05 (s, 3H, CH_3CO), 2.05 (s, 3H, CH_3CO), 2.01 ppm (s, 3H, CH_3CO); ^{13}C NMR (150 MHz, $CDCl_3$) δ 170.7, 170.4, 170.2, 170.0 (4 x CH_3CO), 130.0 (C-1), 121.0 (C-2), 73.2 (C-1´), 68.5 (C-5´), 68.4 (C-3´), 68.3 (C-4´), 68.1 (C-2´), 62.1 (C-6´), 21.0, 20.85, 20.84, 20.8 ppm (4 x CH_3CO); IR ($CDCl_3$, cm^{-1}): 3089, 2982, 2974, 1748, 1645, 1409, 1371, 1235, 1167, 1058, 994, 919, 601, 589; MS (ESI+, m/z (rel.%)): 381.1 (100); HRMS (ESI) calcd. for $C_{16}H_{22}O_9Na$: 381.11560, found 381.11560.

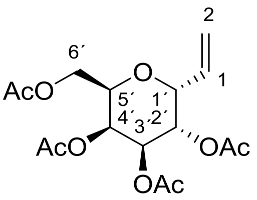

[1] Liu S., Ben R. N. *Org. Lett.* **2005**, *7*, 2385-2388.
[2] Chen, G.; Schmieg. J.; Tsuji, M.; Franck, W. R. *Org. Lett.* **2004**, *6*, 4077-4080.

III Cross-metathesis of α-2 and β-2

Screening of cross-metathesis between α-2 and 3a under various conditions (Table 1). Into a solution of 1-(3,5-bis-*O*-(4-toluoyl)-2-deoxy-α-D-ribofuranosyl)ethene (0.26 mmol, 0.1 g) **α-2** (0.26 mmol, 105 mg), in an appropriate solvent or a mixture of solvents (5 mL) under argon were added Hoveyda–Grubb's 2nd generation catalyst (0.026 mmol, 16 mg) and the alkene **3a** (0.78 mmol, 335 mg). Then the reaction was heated under reflux or in a microwave reactor. For details regarding the solvents used and reaction conditions see Table 1 in the main text. Then all volatiles were removed under reduced pressure and column chromatography of the residue on silica gel (15:1 hexanes/EtOAc) furnished the corresponding product **α-4a**.

General procedure for cross-metathesis between α-2 or β-2 and alkenes 3 (Table 2) Into a solution of 1-(3,5-bis-*O*-(4-toluoyl)-2-deoxy-α-D-ribofuranosyl)ethene (0.26 mmol, 0.105 mg), **α-2** or **β-2** in dry 1,2-dichloroethane (4 mL) under argon were added Hoveyda–Grubb's 2nd generation catalyst (0.026 mmol, 16 mg) and the respective alkene (0.78 mmol). The formed reaction mixture was stirred under reflux for 16 h. All volatiles were removed under reduced pressure and column chromatography of the residue on silica gel (15:1 hexanes/EtOAc) furnished the corresponding product.

(1*E*)-[3,5-Bis-*O*-(4-toluoyl)-2-deoxy-α-D-ribofuranosyl]-{8,8'-μ-(propen-3-yl-disulfido)-[3,3'-como-cobalt(III)-bis-(1,2-dicarbaundecaborate)]} (α-4a). 74%; a red solid; R_f = 0.42

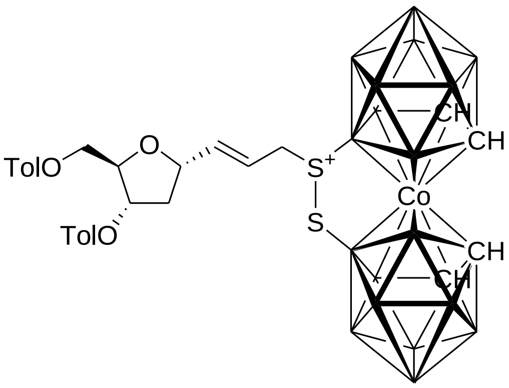

(4/1 hexanes/EtOAc); m.p. 108-109 °C ($CHCl_3$); $[\alpha]_D$ = -46.15° (c = 0.0065 g/ml, $CHCl_3$); ^{1}H NMR (300 MHz, $CDCl_3$) δ 7.94-7.86 (m, 4H, H-Tol), 7.30-7.20 (m, 4H, H-Tol), 6.18 (dd, *J* = 15.2, 6.2, 1H, H-1), 5.85–5.64 (m, 1H, H-2), 5.49 (dt, 1H, 6.5, 3.3 Hz, H-3´), 4.79 (dt, *J* = 12.1, 6.1 Hz, 1H, H-1´), 4.55 (m, 1H, H-4´), 4.49 (d, *J* = 2.5 Hz, 2H, H-5´), 4.39 (dd, *J* = 18.6, 9.0 Hz, 1H, H-3a), 4.08-3.90 (m, 1H, H-3b), 3.71 (bs, 1H, H-carborane), 3.64 (bs, 1H, H-carborane), 3.47 (bs, 1H, H-carborane), 3.31 (bs, 1H, H-carborane), 2.79–2.65 (m, 1H, H-2´a), 2.43 (s, 3H, CH_3-Tol), 2.41 (s, 3H, CH_3-Tol), 2.14 (s, 1H, H-2´b); ^{13}C NMR (75 MHz, $CDCl_3$) δ 166.36 (C=O), 166.26 (C=O), 144.28 (C-Tol), 143.97 (C-Tol), 142.20 (d, *J* = 11.1 Hz, C-1), 129.70 (C-Tol), 129.30 (C-Tol), 129.21 (C-Tol), 126.93 (C-Tol), 126.67 (C-Tol), 119.52 (d, *J* = 44.0 Hz, C-2), 82.11 (d, *J* = 11.8 Hz, C-4´), 78.36 (d, *J* = 19.2 Hz, C-1´), 76.30 (C-3´, 64.52 (C-5´), 51.87 (C-

carborane), 51.44 (C-carborane), 50.48 (C-carborane), 49.15 (d, *J* = 22.1 Hz, C-3), 48.67 (C-carborane), 38.08 (C-2´), 21.70 (CH_3-Tol); ^{11}B NMR (128 MHz, $CDCl_3$) δ 21.33 (s, 2B, B-8, B-8´), 1.43 (bs, 2B, B-10, B-10´), -1.61-14.20 (m, 12B, B-4, B-4´, B-5, B-5´, B-7, B-7´, B-9, B-9´, B-11, B-11´, B-12, B-12´), -23.67 (bs, 2B, B-6, B-6´); IR (KBr, cm^{-1}) ν 2587, 1715, 1609, 1271, 1178, 1108, 1006, 977, 753; MS (ES+, m/z (rel.%)): 802.2 (100), 771.2 (20), 403.1 (10); HRMS (ESI) calcd. for $C_{28}H_{45}O_5{}^{10}B_3B_{15}CoNaS_2$ 802.37166, found 802.37169.

(1*E*)-1-(3,5-Bis-*O*-(4-toluoyl)-2-deoxy-α-D-ribofuranosyl)hept-1-ene (α-4b). Yield:59 %; a

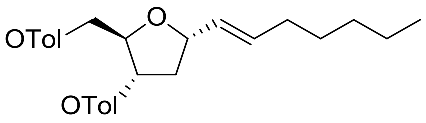

colorless oil; R_f = 0.75 (4/1 hexanes/EtOAc); $[\alpha]_D$ = 12.0° (c = 0.005 g/ml, $CHCl_3$); ^{1}H NMR (300 MHz, $CDCl_3$) δ 7.93 (dd, *J* = 8.2, 2.2 Hz, 4H, H-Tol), 7.28-7.18 (m, 4H, H-Tol), 5.74 (dt, *J* = 15.5, 6.3 Hz, 1H, H-2), 5.62 (dd, *J* = 15.4, 7.1 Hz, 1H, H-1), 5.50 (dt, *J* = 7.1, 3.7 Hz, 1H, H-3´), 4.69 (dt, *J* = 13.6, 6.8 Hz, 1H, H-1´), 4.58–4.39 (m, 3H, H-4´, H-5´), 2.68 (dt, *J* = 13.9, 7.1 Hz, 1H, H-2´a), 2.42 (s, 3H, CH_3-Tol), 2.40 (s, 3H, CH_3-Tol), 2.03 (ddd, *J* = 13.4, 6.2, 4.4 Hz, 3H, H-2´b, H-3), 1.48 – 1.15 (m, 6H, H-4,5,6), 0.96 – 0.79 (m, 3H, H-7); ^{13}C NMR (75 MHz, $CDCl_3$) δ 166.37 (C=O), 166.19 (C=O), 143.98 (C-Tol), 143.73 (C-Tol), 133.92 (C-2), 129.93 (C-1), 129.70 (C-Tol), 129.09 (C-Tol), 127.13 (C-Tol), 127.02 (C-Tol), 81.28 (C-4´), 79.99 (C-1´), 76.60 (C-3´), 64.78 (C-5´), 38.56 (C-2´), 32.15 (C-3), 31.42 (C-5), 28.64 (C-4), 22.52 (C-6), 21.68 (CH_3-Tol), 14.02 (C-7); IR (KBr, cm^{-1}): 1721, 1610, 1281, 1180, 1111, 1006, 970, 752; MS (ES+, m/z (rel.%)): 473.2 (100), 459.2 (10); HRMS (ESI) calcd. for $C_{28}H_{34}O_5Na$: 473.22985, found 473.22987.

(1*E*)-1-(3,5-Bis-*O*-(4-toluoyl)-2-deoxy-α-D-ribofuranosyl)-4,4,5,5,6,6,7,7,8,8,9,9,9-trideca-fluoronon-1-ene (α-4c). Yield: 50%; a colorless oil; R_f = 0.65 (4/1 hexanes/EtOAc); $[\alpha]_D$ =

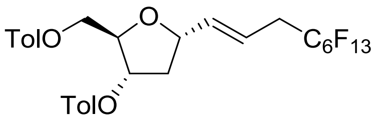

15.0° (c = 0.006 g/ml, $CHCl_3$); ^{1}H NMR (300 MHz, $CDCl_3$) δ 7.92 (td, *J* = 8.8, 2.0 Hz, 4H, H-Tol), 7.28–7.17 (m, 4H, H-Tol), 5.93 (dd, *J* = 15.5, 6.4 Hz, 1H, H-1), 5.74 (dt, *J* = 15.0, 6.9 Hz, 1H, H-2), 5.52 (dt, *J* = 6.7, 3.3 Hz, 1H, H-3´), 4.78 (dt, *J* = 13.1, 6.4 Hz, 1H, H-1´), 4.62–4.42 (m, 3H, H-4´, H-5´), 2.86 (td, *J* = 18.1, 7.0 Hz, 2H, H-3), 2.73 (dt, *J* = 14.2, 7.3 Hz, 1H, H-2´a), 2.41 (s, 3H, CH_3-Tol), 2.41 (s, 3H, CH_3-Tol), 2.07 (ddd, *J* = 13.7, 5.5, 2.9 Hz, 1H, H-2´b); ^{13}C NMR (150 MHz, $CDCl_3$) δ 165.87 (C=O), 165.69 (C=O), 143.66 (C-Tol), 143.39 (C-Tol), 138.04 (C-1), 129.25 (C-Tol), 129.20 (C-Tol), 128.68 (C-Tol, C-2), 126.58 (C-Tol), 126.37 (C-Tol), 117.95 (C-4), 81.36 (C-4´), 78.34 (C-1´), 75.91 (C-3´), 64.11 (C-5´), 37.82 (C-2´), 33.89 (C-3), 29.24 (C-F), 21.18 (CH_3-Tol); ^{19}F NMR (282 MHz, $CDCl_3$) δ -

80.81 (t, *J* = 9.5 Hz, 3F), -112.89-113.21 (m, 2F), -121.95 (bs, 2F), -122.70-123.21 (m, 4F), -125.98-136.28 (td, *J* = 14.8, 6.9 Hz, 2F); IR (KBr, cm^{-1}): 1724, 1613, 1275, 1245, 1207, 1180, 1147, 1111, 1006, 752; MS (ES+, m/z (rel.%)): 767.1 (100), 735.1 (30), 439.0 (20), 217.0 (20); HRMS (ESI) calcd. for $C_{30}H_{25}O_5Na$: 735.13866, found 735.13888.

(1*E*)-2-Phenyl-1-(3,5-bis-*O*-(4-toluoyl)-2-deoxy-α-D-ribofuranosyl)ethene (α-4d). Yield: 68%; a colorless oil; R_f = 0.56 (4/1 hexanes/EtOAc); $[\alpha]_D$ = 5.6° (c = 0.0071 g/ml, $CHCl_3$); ^{1}H NMR (300 MHz, $CDCl_3$) δ 8.03–7.86 (m, 4H, H-Tol), 7.45–7.11 (m, 9H, 4H-Tol, 5H-Ph), 6.66 (d, *J* = 15.9 Hz, 1H, H-2), 6.44–6.29 (dd, *J* = 17.1, 6,8 Hz, 1H, H-1), 5.58 (dt, *J* = 6.6, 3.4 Hz, 1H, H-3´), 4.95 (dt, *J* = 13.1, 6.6 Hz, 1H, H-1´), 4.61 (dd, *J* = 4.7, 3.0 Hz, 1H, H-4´), 4.54 (d, *J* = 4.7 Hz, 2H, H-5´), 2.77 (dt, *J* = 14.0, 7.1 Hz, 1H, H-2´a), 2.41 (s, 3H, CH_3-Tol), 2.41 (s, 3H, CH_3-Tol), 2.18 (ddd, *J* = 13.8, 5.5, 3.7 Hz, 1H, H-2´b); ^{13}C NMR (75 MHz, $CDCl_3$) δ 166.39 (C=O), 166.16 (C=O), 144.04 (C-Tol), 143.82 (C-Tol), 136.53 (C-Ph), 131.23 (C-2), 129.75 (C-Ph), 129.74 (C-Ph), 129.15 (C-Tol), 128.57 (C-1, C-Ph), 127.77 (C-Tol), 127.11 (C-Tol), 126.94 (C-Tol), 126.59 (C-Tol), 81.78 (C-4´), 79.74 (C-1´), 76.49 (C-3´), 64.74 (C-5´), 38.59 (C-2´), 21.70 (CH_3-Tol); IR (KBr, cm^{-1}): 1721, 1610, 1311, 1278, 1177, 1108, 1018, 752; MS (ES+, m/z (rel.%)): 479.1 (100), 407.1 (10); HRMS (ESI) calcd. for $C_{29}H_{28}O_5Na$: 479.18290, found 479.18302.

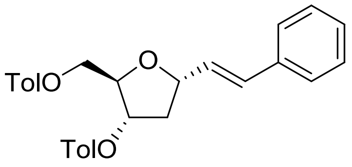

(1*E*)-2-(4-Fluorophenyl)-1-(3,5-bis-*O*-(4-toluoyl)-2-deoxy-α-D-ribofuranosyl)ethene (α-4e). Yield: 60%; a colorless oil; R_f = 0.43 (4/1 hexanes/EtOAc); $[\alpha]_D$ = 0° (c = 0.005 g/ml, $CHCl_3$); ^{1}H NMR (300 MHz, $CDCl_3$) δ 8.02–7.83 (m, 4H, H-Tol), 7.40–7.28 (m, 2H, H-Ph), 7.28–7.12 (m, 4H, H-Tol), 7.00 (t, *J* = 8.6 Hz, 2H, H-Ph), 6.61 (d, *J* = 15.9 Hz, 1H, H-2), 6.27 (dd, *J* = 15.9, 6.6 Hz, 1H, H-1), 5.57 (dt, *J* = 6.6, 3.4 Hz, 1H, H-3´), 4.92 (dt, *J* = 13.1, 6.6 Hz, 1H, H-1´), 4.60 (dd, *J* = 4.6, 2.9 Hz, 1H, H-4´), 4.53 (d, *J* = 4.7 Hz, 2H, H-5´), 2.76 (dt, *J* = 14.0, 7.2 Hz, 1H, H-2´a), 2.40 (s, 6H, CH_3-Tol), 2.17 (ddd, *J* = 13.7, 5.5, 3.7 Hz, 1H, H-2´b); ^{13}C NMR (75 MHz, $CDCl_3$) δ 166.37 (C=O), 166.12 (C=O), 162.41 (d, *J* = 247.0, C-F), 144.09 (C-Tol), 143.84 (C-Tol), 132.70 (C-Ph), 130.05 (C-2), 129.74 (C-Tol), 129.70 (C-Tol), 129.57 (C-Tol), 129.54 (C-Tol), 129.14 (C-1, C-Tol), 128.14 (C-Tol), 128.04 (C-Tol), 127.09 (C-Tol), 126.91 (C-Tol), 115.48 (d, *J* = 21.6, C-Ph), 81.79 (C-4´), 79.61 (C-1´), 76.48 (C-3´), 64.69 (C-2´), 38.55 (C-2´), 21.69 (CH_3-Tol); ^{19}F NMR (282 MHz, $CDCl_3$) δ -114.19 (td, *J* = 8.6, 4.7 Hz, 1F); IR (KBr, cm^{-1}): 1715, 1613,

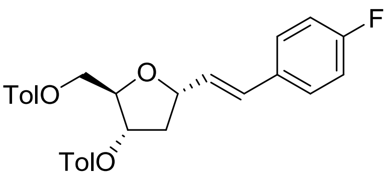

1512, 1311, 1278, 1186, 1105, 1024, 755; MS (ES+, m/z (rel.%)): 497.1 (100), 403.1 (10); HRMS (ESI) calcd. for $C_{29}H_{27}O_5FNa$: 497.17347, found 497.17335.

(1*E*)-2-(4-Trifluoromethylphenylphenyl)-1-(3,5-bis-*O*-(4-toluoyl)-2-deoxy-α-D-ribofuranosyl)ethene (α-4f). Yield: 59%; a colorless solid; R_f = 0.68 (4/1 hexanes/EtOAc); m.p. 139-141 °C ($CHCl_3$); $[\alpha]_D$ = 5.47° (c = 0.0073 g/ml, $CHCl_3$); ^1H NMR (300 MHz, $CDCl_3$) δ 8.03–7.80 (m, 4H, H-Tol), 7.60–7.42 (m, 4H, H-Ph), 7.28–7.12 (m, 4H, H-Tol), 6.69 (d, *J* = 16.0 Hz, 1H, H-2), 6.44 (dd, *J* = 15.9, 6.2 Hz, 1H, H-1), 5.58 (dt, *J* = 6.5, 3.2 Hz, 1H, H-3´), 4.96 (dt, *J* = 12.8, 6.4 Hz, 1H, H-1´), 4.63 (td, *J* = 4.7, 2.7 Hz, 1H, H-4´), 4.53 (d, *J* = 4.7 Hz, 2H, H-5´), 2.77 (dt, *J* = 14.0, 7.2 Hz, 1H, H-2´a), 2.41 (s, 3H, CH_3-Tol), 2.40 (s, 3H, CH_3-Tol), 2.19 (ddd, *J* = 13.8, 5.2, 3.4 Hz, 1H, H-2´b); ^{13}C NMR (75 MHz, $CDCl_3$) δ 166.36 (C=O), 166.07 (C=O), 144.17 (C-Tol), 143.89 (C-Tol), 140.06 (C-Ph), 132.66 (C-2), 129.73 (C-Tol), 129.67 (C-Tol), 129.60 (C-Tol), 129.48 (C-1), 129.16 (C-Tol), 129.13 (C-Ph), 127.04 (C-Ph), 126.86 (C-Tol), 126.82 (C-Tol), 126.69 (C-Tol), 125.74 (dt, *J* = 8.0, 4.0, C-Ph), 125.53 (dt, *J* = 7.5, 3.6, C-Ph), 122.36 (CF_3), 82.00 (C-4´), 79.30 (C-1´), 76.41 (C-3´), 64.62 (C-5´), 38.46 (C-2´), 21.68 (CH_3-Tol); ^{19}F NMR (282 MHz, $CDCl_3$) δ -62.50-62.59 (m, 3F); IR (KBr, cm^{-1}): 1715, 1610, 1329, 1263, 1180, 1120, 1066, 1015, 752; MS (ES+, m/z (rel.%)): 547.1 (100), 407.1 (20), 253.0 (5); HRMS (ESI) calcd. for $C_{30}H_{27}O_5F_3Na$: 547.17028, found 547.17019.

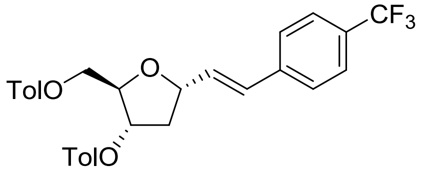

(1*E*)-2-(2-(4,4,5,5-Tetramethyl-1,3,2-dioxaborolan-2-yl)-1-(3,5-bis-*O*-(4-toluoyl)-2-deoxy-α-D-ribofuranosyl)ethene (α-4g). Yield: 66%, pink liquid; R_f = 0.13 (4/1 hexanes/EtOAc); $[\alpha]_D$ = 20.2° (c = 0.0089 g/ml, $CHCl_3$); ^1H NMR (300 MHz, $CDCl_3$) δ 7.96-7.85 (m, 4H, H-Tol), 7.24–7.14 (m, 4H, H-Tol), 6.72 (dd, *J* = 18.0, 5.4 Hz, 1H, H-1), 5.73 (dd, *J* = 18.0, 1.5 Hz, 1H, H-2), 5.50 (dt, *J* = 6.6, 3.3 Hz, 1H, H-3´), 4.85-4.76 (m, 1H, H-1´), 4.61–4.42 (m, 3H, H-4´, H-5´), 2.67 (ddd, *J* = 13.6, 7.9, 6.8 Hz, 1H, H-2´a), 2.40 (s, 6H, CH_3-Tol), 2.12 (ddd, *J* = 13.7, 5.1, 3.4 Hz, 1H, H-2´b), 1.26 (s, 12H, CH_3-Pinacol); ^{13}C NMR (75 MHz, $CDCl_3$) δ 166.34 (C=O), 166.12 (C=O), 152.32 (C-1), 143.88 (C-Tol), 143.76 (C-Tol), 129.77 (C-Tol), 129.71 (C-Tol), 129.10 (C-2), 129.08 (C-Tol), 127.09 (C-Tol), 126.99 (C-Tol), 83.29 (C-Pinacol), 81.81 (C-4´), 80.41 (C-1´), 76.28 (C-3´), 64.70 (C-5´), 37.87 (C-2´), 24.82 (CH_3-Pinacol), 24.73(CH_3-Pinacol), 21.68 (CH_3-Tol); ^{11}B NMR (128 MHz, $CDCl_3$) δ 30.00 (s, 1B); IR (KBr, cm^{-1}): 1718, 1610, 1368,

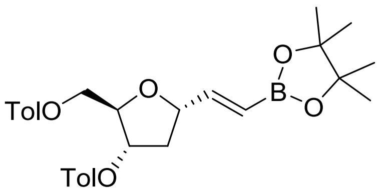

1323, 1275, 1180, 1144, 1108, 1018, 755; MS (ES+, m/z (rel.%)): 529.3 (100), 371.2 (10); HRMS (ESI) calcd. for $C_{29}H_{35}O_7BNa$: 529.23681, found 529.23730.

(1*E*)-[3,5-Bis-*O*-(4-toluoyl)-2-deoxy-β-D-ribofuranosyl]-{8,8'-μ-(propen-3-yl-disulfido)-[3,3'-como-cobalt(III)-bis-(1,2-dicarbaundecaborate)]} (β-4a). Yield: 77%; a red solid; R_f = 0.44 (4/1 hexanes/EtOAc); m.p. 135-137 °C ($CHCl_3$); $[\alpha]_D$ = -12.2° (c = 0.0082 g/ml, $CHCl_3$); ^{1}H NMR (300 MHz, $CDCl_3$) δ 7.90 (dd, *J* = 9.6, 7.9 Hz, 4H, H-Tol), 7.35–7.17 (m, 4H, H-Tol), 6.09 (ddd, *J* = 14.3, 7.9, 5.7 Hz, 1H, H-1), 5.76 (ddd, *J* = 15.3, 8.7, 6.1 Hz, 1H, H-2), 5.51 (d, *J* = 5.9 Hz, 1H, H-3´), 4.80–4.26 (m, 5H, H-1´, H-4´, H-5´, H-3a), 3.92 (dt, *J* =

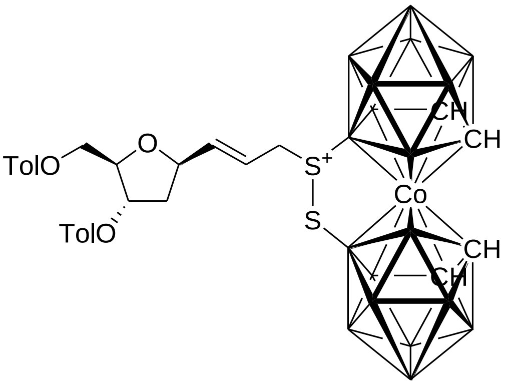

12.9, 6.8 Hz, 1H, H-3b), 3.71 (bs, 1H, H-carborane), 3.66 (bs, 1H, H-carborane), 3.47 (bs, 1H, H-carborane), 3.30 (bs, 1H, H-carborane), 2.42 (s, 6H, CH_3-Tol), 2.36 (t, *J* = 6.6 Hz, 1H, H-2´a), 2.18–1.96 (m, 1H, H-2´b); ^{13}C NMR (75 MHz, $CDCl_3$) δ 166.38 (C=O), 166.11 (C=O), 144.23 (C-Tol), 144.08 (C-Tol), 141.12 (d, *J* = 9.2 Hz, C-1), 129.70 (C-Tol), 129.64 (C-Tol), 129.37 (C-Tol), 129.34 (C-Tol), 129.22 (C-Tol), 126.90 (C-Tol), 126.81 (C-Tol), 119.96 (d, *J* = 92.0 Hz, C-1), 83.11 (C-4´), 78.35 (d, *J* = 34.9 Hz, C-1´), 77.25 (C-3´), 64.48 (C-5´), 51.75 (d, *J* = 24.4 Hz, C-3), 50.43 (C-carborane), 48.89 (C-carborane), 48.61 (C-carborane), 39.38 (C-2´), 21.73 (CH_3-Tol); ^{11}B NMR (128 MHz, $CDCl_3$): δ 21.30 (s, 2B, B-8, B-8´), 1.16 (bs, 2B, B-10, B-10´), -1.80-14.39 (m, 12B, B-4, B-4´, B-5, B-5´, B-7, B-7´, B-9, B-9´, B-11, B-11´, B-12, B-12´), -23.88 (bs, 2B, B-6, B-6´); IR (KBr, cm^{-1}): 2589, 1715, 1609, 1269, 1176, 1107, 1006, 977, 754; MS (ES+, m/z (rel.%)): 802.1 (100), 403.0 (30); HRMS (ESI) calcd. for $C_{28}H_{45}O_5{}^{10}B_3B_{15}CoNaS_2$: 802.37166, found 802.37167.

(1*E*)-1-(3,5-Bis-*O*-(4-toluoyl)-2-deoxy-β-D-ribofuranosyl)hept-1-ene (β-4b). Yield: 64%; a

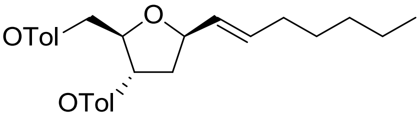

colorless oil; R_f = 0.77 (4/1 hexanes/EtOAc); $[\alpha]_D$ = 7.4° (c = 0.0051 g/ml, $CHCl_3$); ^{1}H NMR (300 MHz, $CDCl_3$) δ 7.94 (dd, *J* = 8.2, 3.0 Hz, 4H, H-Tol), 7.24 (dd, *J* = 8.1, 4.9 Hz, 4H, H-Tol), 5.81 (dt, *J* = 15.4, 6.7 Hz, 1H, H-1), 5.56–5.39 (m, 2H, H-2, H-3´), 4.63 (ddd, *J* = 10.6, 7.5, 5.1 Hz, 1H, H-1´), 4.51 (d, *J* = 4.3 Hz, 2H, H-5´), 4.38 (dt, *J* = 6.6, 3.1 Hz, 1H, H-4´), 2.42 (s, 3H, CH_3-Tol), 2.40 (s, 3H, CH_3-Tol), 2.26 (dd, *J* = 13.7, 5.1 Hz, 1H, H-2´a), 2.12-1.97 (m, 3H, H-2´b, H-3), 1.45 – 1.17 (m, 6H, H-4,5,6), 0.88 (t, *J* = 6.6 Hz, 3H, H-7);

^{13}C NMR (75 MHz, $CDCl_3$) δ 166.34 (C=O), 166.09 (C=O), 144.00 (C-Tol), 143.71 (C-Tol), 135.05 (C-2), 129.72 (C-Tol), 129.69 (C-Tol), 129.14 (C-Tol), 129.11 (C-Tol), 128.83 (C-1), 127.20 (C-Tol), 127.09 (C-Tol), 82.34 (C-4´), 80.29 (C-1´), 77.27 (C-3´), 64.83 (C-5´), 39.34 (C-2´), 32.20 (C-3), 31.39 (C-5), 28.62 (C-4), 22.50 (C-6), 21.70 (CH_3-Tol), 21.68 (CH_3-Tol), 14.03 (C-7); IR (KBr, cm^{-1}): 1718, 1610, 1278, 1174, 1114, 1018, 755; MS (ES+, m/z (rel.%)): 408.1 (10), 178.1 (20), 165.1 (20), 149.0 (100), 119.1 (80), 91.1 (40); HRMS (ESI) calcd. for $C_{28}H_{34}O_5Na$: 473.22985, found 473.22976.

(1*E*)-1-(3,5-Bis-*O*-(4-toluoyl)-2-deoxy-β-D-ribofuranosyl)-4,4,5,5,6,6,7,7,8,8,9,9,9-trideca-fluoronon1-en (β-4c). Yield: 48%; a colorless oil; R_f = 0.65 (4/1 hexanes/EtOAc); $[\alpha]_D$ = 6.45° (c = 0.0062 g/ml, $CHCl_3$); ^1H NMR (300 MHz, $CDCl_3$) δ 7.94 (d, *J* = 8.0 Hz, 4H, H-Tol), 7.35–7.14 (m, 4H, H-Tol), 5.84-5.77 (m, 2H, H-1, H-2), 5.52 (d, *J* = 5.5 Hz, 1H, H-3´), 4.72 (dt, *J* = 10.0, 4.8 Hz, 1H, H-1´), 4.53 (t, *J* = 4.1 Hz, 2H, H-5´), 4.43 (td, *J* = 4.2, 2.0 Hz, 1H, H-4´), 2.83 (td, *J* = 18.2, 4.8 Hz, 2H, H-3), 2.42 (s, 3H, CH_3-Tol), 2.41 (s, 3H, CH_3-Tol), 2.34 (dd, *J* = 13.8, 5.3 Hz, 1H, H-2´a), 2.08 (ddd, *J* = 13.7, 10.6, 6.1 Hz, 1H, H-2´b); ^{13}C NMR (75 MHz, $CDCl_3$) δ 166.29 (C=O), 166.06 (C=O), 144.14 (C-Tol), 143.83 (C-Tol), 137.26 (C-1), 129.68 (C-2, C-Tol), 129.17 (C-Tol), 129.13 (C-Tol), 127.09 (C-Tol), 126.93 (C-Tol), 119.52 (C-4), 82.75 (C-4´), 79.02 (C-1´), 64.58 (C-5´), 39.16 (C-2´), 34.43 (C-3), 21.67 (CH_3-Tol), 21.59 (CH_3-Tol); ^{19}F NMR (282 MHz, $CDCl_3$) δ -80.84 (t, *J* = 9.5 Hz, 3F), -113.09 (tq, *J* = 17.0, 11.6, 8.1 Hz, 2F), -121.79-122.16 (m, 2F), -122.75-123.22 (m, 4F), -126.17 (td, *J* = 14.8, 6.9 Hz, 2F); IR (KBr, cm^{-1}): 1718, 1610, 1317, 1275, 1242, 1204, 1180, 1141, 1108, 755; MS (ES+, m/z (rel.%)): 440.0 (20), 399.0 (10), 136.0 (30), 119.0 (100), 91.1 (60); HRMS (ESI) calcd. for $C_{30}H_{25}O_5Na$: 735.13866, found 735.13893.

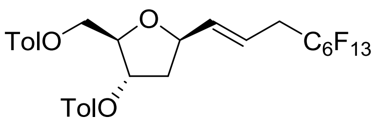

(1*E*)-2-Phenyl-1-(3,5-bis-*O*-(4-toluoyl)-2-deoxy-β-D-ribofuranosyl)ethene (β-4d). Yield: 69%; a colorless solid; R_f = 0.58 (4/1 hexanes/EtOAc); m.p. 110-112 °C ($CHCl_3$); $[\alpha]_D$ = 46.15° (c = 0.0065 g/ml, $CHCl_3$); ^1H NMR (300 MHz, $CDCl_3$) δ 7.97 (d, *J* = 8.1 Hz, 4H, H-Tol), 7.41–7.17 (m, 9H, 4H-Tol, 5H-Ph), 6.70 (d, *J* = 15.9 Hz, 1H, H-2), 6.22 (dd, *J* = 15.9, 7.1 Hz, 1H, H-1), 5.57 (dt, *J* = 6.1, 1.5 Hz, 1H, H-3´), 4.94–4.80 (m, 1H, H-1´), 4.66–4.50 (m, 2H, H-5´), 4.47 (td, *J* = 4.3, 2.1 Hz, 1H, H-4´), 2.44 (s, 3H, CH_3-Tol), 2.41 (s, 3H, CH_3-Tol), 2.37 (dd, *J* = 5.2, 1.3 Hz, 1H, H-2´a), 2.19 (ddd, *J* = 13.8, 10.5, 6.0 Hz, 1H, H-2´b); ^{13}C NMR (75 MHz, $CDCl_3$) δ 166.36 (C=O), 166.11 (C=O), 144.11 (C-

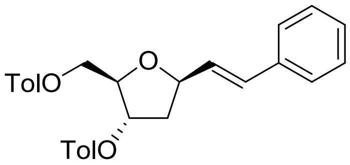

Tol), 143.79 (C-Tol), 136.35 (C-Ph), 132.31 (C-2), 129.74 (C-Ph), 129.19 (C-Tol), 129.17 (C-Tol), 128.54 (C-Ph), 128.44 (C-Ph), 127.88 (C-1), 127.17 (C-Tol), 127.03 (C-Tol), 126.61 (C-Tol), 82.64 (C-4´), 80.08 (C-1´), 77.18 (C-3´), 64.76 (C-5´), 39.47 (C-2´), 21.72 (CH_3-Tol), 21.69 (CH_3-Tol); IR (KBr, cm^{-1}): 1721, 1610, 1272, 1177, 1111, 1018, 752; MS (ES+, m/z (rel.%)): 479.1 (100), 405.1 (10); HRMS (ESI) calcd. for $C_{29}H_{28}O_5Na$: 479.18290, found 479.18280.

(1*E*)-2-(4-Fluorophenyl)-1-(3,5-bis-*O*-(4-toluoyl)-2-deoxy-β-D-ribofuranosyl)ethene (β-4e). Yield: 58%; a colorless oil; R_f = 0.44 (4/1 hexanes/EtOAc); $[\alpha]_D$ = -38.5° (c = 0.0065 g/ml, $CHCl_3$); ^1H NMR (300 MHz, $CDCl_3$) δ 8.03–7.88 (m, 4H, H-Tol), 7.37–7.15 (m, 6H, 4H-Tol, 2h-Ph), 7.07–6.89 (m, 2H, H-Ph), 6.65 (d, *J* = 15.9 Hz, 1H, H-2), 6.12 (dd, *J* = 15.9, 7.0 Hz, 1H, H-1), 5.56 (dt, *J* = 6.2, 1.5 Hz, 1H, H-3´), 4.85 (dt, *J* = 11.5, 5.9 Hz, 1H, H-1´), 4.63-4.49 (m, 2H, H-5´), 4.46 (td, *J* = 4.3, 2.0 Hz, 1H, H-4´), 2.43 (s, 3H, CH_3-Tol), 2.41 (s, 3H, CH_3-Tol), 2.36 (dd, *J* = 5.1, 1.3 Hz, 1H, H-2´a), 2.18 (ddd, *J* = 13.8, 10.5, 6.0 Hz, 1H, H-2´b); ^{13}C NMR (75 MHz, $CDCl_3$) δ 166.33 (C=O), 166.10 (C=O), 162.5 (d, *J* = 247.3, C-F), 144.13 (C-Tol), 143.82 (C-Tol), 132.52 (C-Ph), 131.10 (C-2), 129.72 (C-Tol), 129.19 (C-Tol), 129.16 (C-Tol), 128.20 (C-Tol), 128.09 (C-1), 127.15 (C-Tol), 127.00 (C-Tol), 115.46 (d, *J* = 21.6, C-Ph), 82.64 (C-4´), 79.95 (C-1´), 77.14 (C-3´), 64.71 (C-5´), 39.44 (C-2´), 21.68 (CH_3-Tol); ^{19}F NMR (282 MHz, $CDCl_3$) δ -114.02 (td, *J* = 8.6, 4.7 Hz, 1F); IR (KBr, cm^{-1}): 1724, 1610, 1509, 1308, 1275, 1177, 1108, 1018, 752; MS (ES+, m/z (rel.%)): 497.1 (100), 403.1 (10); HRMS (ESI) calcd. for $C_{29}H_{27}O_5FNa$: 497.17347, found 497.17338.

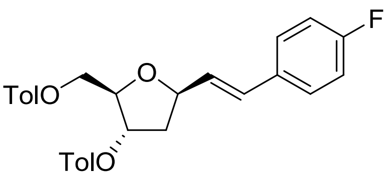

(1*E*)-2-(4-Trifluoromethylphenyl)-1-(3,5-bis-*O*-(4-toluoyl)-2-deoxy-β-D-ribofuranosyl)-ethene (β-4f). Yield: 61%; a colorless solid; R_f = 0.69 (4/1 hexanes/EtOAc); m.p. 107-109 °C ($CHCl_3$); $[\alpha]_D$ = -36.4° (c = 0.0077 g/ml, $CHCl_3$); ^1H NMR (300 MHz, $CDCl_3$) δ 7.96 (dt, *J* = 8.4, 2.3 Hz, 4H, H-Tol), 7.54 (d, *J* = 8.2 Hz, 2H, H-Ph), 7.42 (d, *J* = 8.1 Hz, 2H, H-Ph), 7.31–7.16 (m, 4H, H-Tol), 6.72 (d, *J* = 15.9 Hz, 1H, H-2), 6.31 (dd, *J* = 15.9, 6.6 Hz, 1H, H-1), 5.58 (dt, *J* = 6.3, 1.6 Hz, 1H, H-3´), 4.89 (dt, *J* = 11.2, 5.9 Hz, 1H, H-1´), 4.63 (dd, *J* = 11.6, 4.1 Hz, 1H, H-5´a), 4.58 – 4.45 (m, 2H, H-5´b, H-4´), 2.48-2.36 (m, 7H, CH_3-Tol, H-2´a), 2.20 (ddd, *J* = 13.8, 10.5, 6.1 Hz, 1H, H-2´b); ^{13}C NMR (75 MHz, $CDCl_3$) δ 166.31 (C=O), 166.09 (C=O), 144.18 (C-Tol), 143.88 (C-Tol), 139.88 (C-

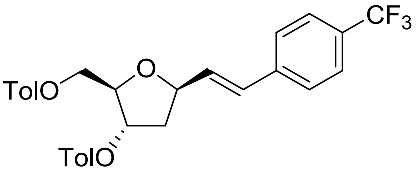

Ph), 131.24 (C-2), 130.45 (C-1), 129.72 (C-Tol), 129.21 (C-Tol), 129.17 (C-Tol), 127.11 (C-Tol), 126.95 (C-Tol), 126.73 (C-Ph), 125.47 (dt, J = 7.2, 3.5 Hz, C-Ph), 122.36 (C-Ph), 82.80 (C-4´), 79.56 (C-1´), 77.06 (C-3´), 64.62 (C-5´), 39.39 (C-2´), 21.65 (CH_3-Tol); ^{19}F NMR (282 MHz, $CDCl_3$) δ -62.52 (bs, 3F); IR (KBr, cm^{-1}): 1709, 1613, 1332, 1278, 1180, 1120, 1066, 964, 755; MS (ES+, m/z (rel.%)): 547.1 (100), 405.1 (30), 253.0 (5); HRMS (ESI) calcd. for $C_{30}H_{27}O_5F_3Na$: 547.17028, found 547.17019.

(1*E*)-2-(2-(4,4,5,5-Tetramethyl-1,3,2-dioxaborolan-2-yl)-1-(3,5-bis-*O*-(4-toluoyl)-2-deoxy-β-D-ribofuranosyl)ethene (β-4g). Yield: 64%; pink oil; R_f = 0.15 (4/1 hexanes/EtOAc); $[\alpha]_D$ = 0° (c = 0.0119 g/ml, $CHCl_3$); ^1H NMR (300 MHz, $CDCl_3$) δ 8.03–7.83 (m, 4H, H-Tol), 7.25 (d, J = 7.4 Hz, 4H, H-Tol), 6.62 (dd, J = 18.0, 6.0 Hz, 1H, H-1), 5.76 (dd, J = 18.1, 1.3 Hz, 1H, H-2), 5.50 (d, J = 5.9 Hz, 1H, H-3´), 4.72 (dt, J = 10.6, 5.5 Hz, 1H, H-1´), 4.46 (m, 3H, H-4´, H-5´), 2.42 (s, 3H, CH_3-Tol), 2.40 (s, 3H, CH_3-Tol), 2.32 (ddd, J = 13.8, 5.4, 1.3 Hz, 1H, H-2´a), 2.17–2.03 (m, 1H, H-2´b), 1.26 (s, 12H, CH_3-Pinacol); ^{13}C NMR (75 MHz, $CDCl_3$) δ 166.34 (C=O), 166.04 (C=O), 150.78 (C-1), 144.06 (C-Tol), 143.68 (C-Tol), 129.77 (C-Tol), 129.71 (C-Tol), 129.15 (C-2), 129.10 (C-Tol), 127.12 (C-Tol), 126.99 (C-Tol), 83.39 (C-Pinacol), 82.62 (C-4´), 80.83 (C-1´), 76.95 (C-3´), 64.74 (C-5´), 38.66 (C-2´), 24.82 (CH_3-Pinacol), 24.73 (CH_3-Pinacol), 21.70 (CH_3-Tol), 21.68 (CH_3-Tol); ^{11}B NMR (128 MHz, $CDCl_3$) δ 22.46 (s, 1B); IR (KBr, cm^{-1}): 1719, 1611, 1369, 1270, 1177, 1106, 995, 754; MS (ES+, m/z (rel.%)): 529.3 (100), 371.1 (10); HRMS (ESI) calcd. for $C_{29}H_{35}O_7BNa$: 529.23681, found 529.23711.

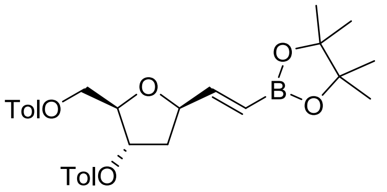

Cross-coupling of β-4g with phenyl iodide to β-4d under Suzuki conditions.
(1*E*)-2-Phenyl-1-(3,5-bis-*O*-(4-toluoyl)-2-deoxy-β-D-ribofuranosyl)ethene (β-4d). Into a solution of **β-4g** (0.1 mmol, 51 mg) in dry dimethoxyethane (3 mL) was added 5% tetrakis(triphenylphospine)palladium(0) catalyst (0.005 mmol, 5.7 mg), phenyl iodide (0.3 mmol, 61 mg) and dry potassium carbonate (0.3 mmol, 41 mg) and the flask was filled with argon. The reaction mixture was then stirred at 85 °C for 16 h. All volatiles were removed under reduced pressure and column chromatography of the residue on silica gel (15:1 hexanes/EtOAc) furnished 23 mg (51%) of the title compound as a colorless oil. Spectral characteristics were in agreement with the previously obtained values.

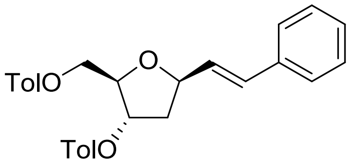

IV Catalytic Reduction of the β-4b–β-4d.

1-(3,5-Bis-*O*-(4-toluoyl)-2-deoxy-β-D-ribofuranosyl)heptane (β-5b). Into a solution of **β-4b**

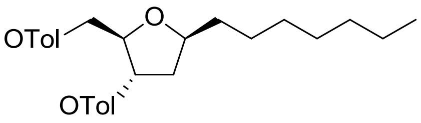

(0.01 mmol, 50 mg) in dry EtOAc (4 mL) was added 10% Pd/C catalyst (0.001 mmol, 118 mg) and the flask was filled with a hydrogen atmosphere. The formed reaction mixture was then stirred at 20 °C for 1 h. All volatiles were removed under reduced pressure and column chromatography of the residue on silica gel (10:1 hexanes/EtOAc) furnished 45 mg (88%) of the title compound as a colorless oil. R_f = 0.80 (4/1 hexanes/EtOAc); $[\alpha]_D$ = 28.9° (c = 0.0114 g/ml, $CHCl_3$); ^{1}H NMR (300 MHz, $CDCl_3$) δ 7.94 (dd, *J* = 8.0, 3.6 Hz, 4H, H-Tol), 7.34–7.14 (m, 4H, H-Tol), 5.54–5.39 (m, 1H, H-3´), 4.59–4.41 (m, 2H, H-5´), 4.34 (td, *J* = 4.4, 2.3 Hz, 1H, H-4´), 4.21 (ddt, *J* = 10.8, 5.6, 5.5 Hz, 1H, H-1´), 2.42 (s, 3H, CH_3-Tol), 2.41 (s, 3H, CH_3-Tol), 2.21 (dd, *J* = 13.6, 4.9 Hz, 1H, H-2´a), 1.93 (ddd, *J* = 13.7, 10.5, 6.3 Hz, 1H, H-2´b), 1.78–1.13 (m, 12H, H-1,2,3,4,5,6), 0.88 (t, *J* = 6.8, 3H, H-7); ^{13}C NMR (75 MHz, $CDCl_3$) δ 166.35 (C=O), 166.15 (C=O), 143.95 (C-Tol), 143.68 (C-Tol), 129.70 (C-Tol), 129.68 (C-Tol), 129.12 (C-Tol), 129.10 (C-Tol), 127.24 (C-Tol), 127.15 (C-Tol), 82.19 (C-4´), 79.45 (C-1´), 77.17 (C-3´), 64.81 (C-5´), 38.60 (C-2´), 35.22 (C-1), 31.78 (C-5), 29.66 (C-3), 29.21 (C-4), 25.93 (C-2), 22.66 (C-6), 21.67 (CH_3-Tol), 14.09 (C-7); IR (KBr, cm^{-1}): 1718, 1611, 1406, 1270, 1175, 1120, 1104, 1018, 981, 753; MS (ES+, m/z (rel.%)): 475.3 (100), 461.2 (10), 453.3 (10), 317.2 (10); HRMS (ESI) calcd. for $C_{28}H_{36}O_5Na$: 475.2455, found 475.24542.

1-(3,5-Bis-*O*-(4-toluoyl)-2-deoxy-β-D-ribofuranosyl)-4,4,5,5,6,6,7,7,8,8,9,9,9-tridecafluorononane (β-5c). Into a solution of **β-4c** (0.005 mmol, 33 mg) in dry ethyl acetate (1.5 mL)

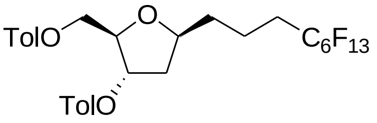

was added 10% Pd/C catalyst (0.0016 mmol, 17 mg) and the flask was filled with a hydrogen atmosphere. The formed reaction mixture was then stirred at 20 °C for 1 h. All volatiles were removed under reduced pressure and column chromatography of the residue on silica gel (12:1 hexanes/EtOAc) afforded 19 mg (57%) of the title compound as a colorless solid. R_f = 0.52 (4/1 hexanes/EtOAc); m.p. 54-55 °C ($CHCl_3$); $[\alpha]_D$ = 8.3° (c = 0.0181 g/ml, $CHCl_3$); ^{1}H NMR (300 MHz, $CDCl_3$) δ 7.93 (dd, *J* = 8.1, 5.1 Hz, 4H, H-Tol), 7.37–7.15 (m, 4H, H-Tol), 5.28 (dt, *J* = 7.6, 5.0 Hz, 1H, H-3´), 4.56 (dd, *J* = 11.8, 3.4 Hz, 1H, H-5´), 4.38 (dd, *J* = 11.8, 6.6 Hz, 1H, H-5´), 4.18 (td, *J* = 6.1, 3.4 Hz, 1H, H-4´), 2.42 (s, 3H, CH_3-Tol), 2.41 (s, 3H, CH_3-Tol), 2.18–1.76 (m, 4H, H-3, H-2´a, H-1´), 1.70–1.32 (m, 5H, H-2´b, H-1, H-2); ^{13}C NMR (75 MHz, $CDCl_3$) δ 167.05 (C=O), 166.49 (C=O), 144.11 (C-Tol), 144.06 (C-Tol),

129.77 (C-Tol), 129.20 (C-Tol), 129.15 (C-Tol), 126.98 (C-Tol), 126.83 (C-Tol), 77.22 (C-4´), 74.91 (C-1´), 71.75 (C-3´), 65.81 (C-5´), 30.76 (C-F), 30.29 (C-2´), 28.92 (C-1), 25.01 (C-3), 21.67 (CH_3-Tol), 20.01 (C-2); ^{19}F NMR (282 MHz, $CDCl_3$) δ -80.81, -80.93 (d, *J* = 9.5 Hz, 3F), -114.39 (bs, 2F), -121.96 (bs, 2F), -122.90 (bs, 2F), -123.59 (bs, 2F), -126.15 (bs, 2F); IR (KBr, cm^{-1}): 1718, 1611, 1310, 1271, 1241, 1206, 1180, 1144, 1108, 1019, 754; MS (ES+, m/z (rel.%)) 755.2 (10), 739.2 (100), 722.6 (10), 699.2 (10), 581.2 (10); HRMS (ESI) calcd. for $C_{30}H_{29}O_5F_{13}Na$: 739.16996, found 739.17014.

2-(Phenyl)-1-(3,5-bis-*O*-(4-toluoyl)-2-deoxy-β-D-ribofuranosyl)ethane (β-5d). Into a solution of **β-4d** (0.012 mmol, 56 mg) in dry EtOAc (3 mL) was added 10% Pd/C catalyst (0.004 mmol, 43 mg) and the flask was filled with hydrogen. The reaction mixture was then stirred at 20 °C for 1 h. All volatiles were removed under reduced pressure and column chromatography of the residue on silica gel (10:1 hexanes/EtOAc) furnished 49 mg (87%) of the title compound as a colorless oil. R_f = 0.65 (4/1 hexanes/EtOAc); $[\alpha]_D$ = 17.6° (c = 0.018 g/ml, $CHCl_3$); ^{1}H NMR (300 MHz, $CDCl_3$) δ 7.95 (td, *J* = 7.2, 6.1, 2.7 Hz, 4H, H-Tol), 7.37–7.08 (m, 9H, 4H-Tol, 5H-Ph), 5.57–5.42 (m, 1H, H-3´), 4.65–4.44 (m, 2H, H-5´), 4.37 (tt, *J* = 4.6, 2.2 Hz, 1H, H-4´), 4.33–4.14 (m, 1H, H-1´), 2.93–2.55 (m, 2H, H-2), 2.43 (s, 3H, CH_3-Tol), 2.41 (s, 3H, CH_3-Tol), 2.24 (dd, *J* = 13.6, 5.0 Hz, 1H, H-2´a), 2.08-1.84 (m, 3H, H-2´b, H-1); ^{13}C NMR (75 MHz, $CDCl_3$) δ 166.37 (C=O), 166.17 (C=O), 144.01 (C-Tol), 143.76 (C-Tol), 141.76 (C-Ph), 129.70 (C-Tol), 129.15 (C-Tol), 128.37 (C-Tol), 127.23 (C-Ph), 127.09 (C-Ph), 125.89 (C-Ph), 82.30 (C-4´), 78.67 (C-3´), 64.76 (C-1´), 38.60 (C-2´), 36.97 (C-1), 32.25 (C-2), 21.71 (CH_3-Tol); IR (KBr, cm^{-1}): 1721, 1610, 1299, 1275, 1177, 1108, 1018, 752; MS (ES+, m/z (rel.%)): 481.1 (100), 459.1 (10), 405.1 (30), 323.1 (10); HRMS (ESI) calcd. for $C_{23}H_{24}O_5$: 481.19855, found 481.29857.

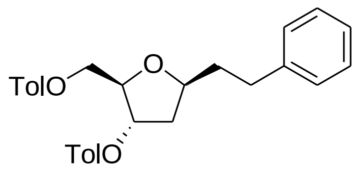

V Deprotection of toluoyl groups from 4.

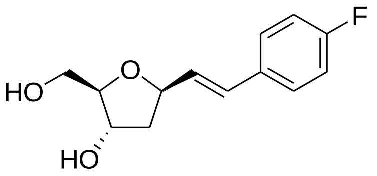

(1*E*)-2-(4-Fluorophenyl)-1-(2-deoxy-β-D-ribofuranosyl)ethene (β-6e). Into a solution of **β-5e** (0.01 mmol, 48 mg) in the 9:1 mixture of MeOH/H_2O (2.2 mL) was added K_2CO_3 (0.025 mmol, 35 mg). The formed reaction mixture was then stirred at 20 °C for 16 h. All volatiles were removed under reduced pressure and column chromatography of the residue on silica gel (1:1 hexanes/EtOAc) furnished 21.5 mg (89%) of the title compound as a colorless solid. R_f = 0.15 (1/1 hexanes/EtOAc); m.p. 82-84 °C ($CHCl_3$); $[\alpha]_D$ = 39.3° (c = 0.0061 g/ml, $CHCl_3$); ^{1}H NMR (300 MHz, MeOD) δ 7.45 (ddd, *J* = 8.7, 5.6, 2.7 Hz, 2H, H-Ph), 7.14–6.97 (m, 2H, H-Ph), 6.65 (d, *J* = 15.9 Hz, 1H, H-2), 6.22 (dd, *J* = 15.9, 7.3 Hz, 1H, H-1), 4.74 (dt, *J* = 10.2, 6.4 Hz, 1H, H-1´), 4.31 (dt, *J* = 6.1, 2.2 Hz, 1H, H-3´), 3.90 (td, *J* = 5.1, 2.5 Hz, 1H, H-4´), 3.61 (d, *J* = 4.9 Hz, 2H, H-5´), 2.06 (ddd, *J* = 13.0, 5.6, 2.0 Hz, 1H, H-2´a), 1.94 (ddd, *J* = 13.0, 9.9, 5.8 Hz, 1H, H-2´b); ^{13}C NMR (75 MHz, MeOD) δ 166.31 (d, *J* = 245.4 Hz, C-F), 137.07 (d, *J* = 3.2 Hz, C-Ph), 133.98 (C-2), 133.28 (C-1), 131.82 (d, *J* = 8.1 Hz, C-Ph), 118.83 (d, *J* = 21.8 Hz, C-Ph), 91.49 (C-4´), 83.39 (C-1´), 76.94 (C-3´), 66.59 (C-5´), 45.17 (C-2´); ^{19}F NMR (282 MHz, MeOD) δ -116.70 (tt, *J* = 9.5, 5.3 Hz, 1F); IR (KBr, cm^{-1}): 3395, 3342, 2929, 2893, 1601, 1512, 1233, 1048, 973, 851, 812; MS (ES+, m/z (rel.%)): 261.1 (100); HRMS (ESI) calcd. for $C_{13}H_{15}O_3FNa$: 261.09029, found 261.09021.

(1*E*)-1-(2-Deoxy-β-D-ribofuranosyl)heptane (β-7b). Into a solution of **β-5b** (0.007 mmol, 30 mg) in the 9:1 mixture of MeOH/H_2O (1.6 mL) was added K_2CO_3 (0.016 mmol, 23 mg). The formed reaction mixture was then stirred at 20 °C for 16 h. All volatiles were removed under reduced pressure and column chromatography of the residue on silica gel (1:1 hexanes/EtOAc) furnished 13.3 mg (93%) of the title compound as a colourless oil. R_f = 0.12 (4/1 hexanes/EtOAc); $[\alpha]_D$ = 22.8° (c = 0.0114 g/ml, $CHCl_3$); ^{1}H NMR (300 MHz, MeOD) δ 4.20 (dt, *J* = 6.4, 2.3 Hz, 1H, H-3´), 4.11 (dt, *J* = 11.1, 5.7 Hz, 1H, H-1´), 3.78 (td, *J* = 5.1, 2.8 Hz, 1H, H-4´), 3.55 (d, *J* = 5.1 Hz, 2H, H-5´), 1.93 (ddd, *J* = 13.0, 5.3, 2.0 Hz, 1H, H-2´a), 1.67–1.25 (m, 13H, H-2´b, H-1,2,3,4,5,6), 0.93 (t, *J* = 6.9, 3H, H-7); ^{13}C NMR (75 MHz, MeOD) δ 87.13 (C-4´), 78.59 (C-1´), 72.82 (C-3´), 62.68 (C-5´), 40.46 (C-2´), 35.27 (C-1), 31.59 (C-5), 29.41 (C-3), 29.00 (C-4), 25.84 (C-2), 22.31 (C-6), 13.02 (C-7); IR (KBr, cm^{-1}): 3345, 2925, 2855, 1465, 1378, 1103, 1045; MS (ES+, m/z (rel.%)): 455.2 (10), 344.2 (10),

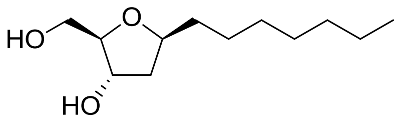

307.0 (10), 253.1 (10), 239.1 (100), 225.1 (10); HRMS (ESI) calcd. for $C_{12}H_{24}O_3Na$: 239.16177, found 239.16173.

VI Cross-metathesis of 8

General procedure. Into a solution of 1-(2´,3´,4´,6´-tetra-*O*-acetyl-α-D-galactopyranosyl)ethene (**8**, 0.28 mmol, 0.1 g) in dry CH_2Cl_2 (5 mL) under argon was added Hoveyda–Grubb's 2nd generation catalyst (0.014 mmol, 8.7 mg), CuI (0.014 mmol, 2.7 mg) and the respective alkene (0.42 mmol). The formed reaction mixture was stirred under reflux for 16 h. All volatiles were removed under reduced pressure and column chromatography of the residue on silica gel (4:1 hexanes/EtOAc) furnished the corresponding product **9**.

(1*E*)-1-(2´,3´,4´,6´-Tetra-*O*-acetyl-α-D-galactopyranosyl)hept-1-ene (9b). Yield: 80%; a

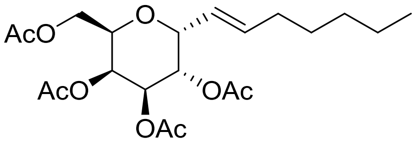

colorless oil; R_f = 0.2 (3/1 hexane/EtOAc); $[\alpha]_D$ = 104.9° (c = 0.609 g/ml, $CHCl_3$); ^{1}H NMR (500 MHz, $CDCl_3$) δ 5.87 (ddd, *J* = 14.0, 6.9, 1.3 Hz, 1H, H-2), 5.61 (ddd, *J* = 15.5, 6.4, 1.3 Hz, 1H, H-1), 5.41 (dd, *J* = 3.5, 1.6 Hz, 1H, H-4´), 5.28 (dd, *J* = 10.5, 6.0 Hz, 1H, H-2´), 5.16 (dd, *J* = 10.5, 3.3 Hz, 1H, H-3´), 4.75 (t, *J* = 6.2 Hz, 1H, H-1´), 4.15 (m, 1H, H-6a´), 4.12 – 4.04 (m, 2H, H-6b´, H-5´), 2.14 (s, 3H, CH_3CO), 2.13 – 2.05 (m, 2H, CH_2-3), 2.04 (s, 3H, CH_3CO), 2.02 (s, 3H, CH_3CO), 2.01 (s, 3H, CH_3CO), 1.45 – 1.35 (m, 2H, CH_2-4),1.35 – 1.20 (m, 4H, CH_2-5, CH_2-6), 0.90 (q, *J* = 6.9 Hz, 3H, CH_3-7); ^{13}C NMR (125 MHz, $CDCl_3$) δ 170.4, 170.2, 170.1, 169.8 (4 × CH_3*C*O), 138.8 (C-2), 121.1 (C-1), 73.2 (C-1´), 68.3 (C-5´), 68.1 (C-3´), 68.1 (C-2´), 67.9 (C-4´), 62.7 (C-6´), 33.4(C-3), 32.0 (C-5), 29.4 (C-4), 23.2 (C-6), 21.5, 21.4, 21.4, 21.3 (4 × *C*H_3CO), 14.7 (C-7); IR ($CDCl_3$, cm^{-1}): 3029, 2960, 1747, 1664, 1433, 1233, 1167, 1054, 977, 603; MS (ESI+, m/z (rel.%)): 451.0 (100); HRMS (ESI) calcd. for $C_{21}H_{32}O_9Na$: 451.19385, found 451.19376.

(1*E*)-1-(2´,3´,4´,6´-Tetra-*O*-acetyl-α-D-galactopyranosyl)-4,4,5,5,6,6,7,7,8,8,9,9,9-trideca-fluoronon-1-ene (9c). Yield: 79%; a colorless foam; R_f = 0.2

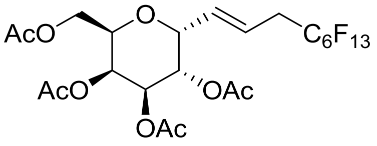

(3/1 hexane/EtOAc); $[\alpha]_D$ = 73.6° (c = 1.186 g/ml, $CHCl_3$); ^{1}H NMR (400 MHz, $CDCl_3$) δ 6.02 – 5.83 (m, 2H, H-1, H-2), 5.43 (d, *J* = 3.3 Hz, 1H, H-4´), 5.34 (dd, *J* = 10.3, 5.9 Hz, 1H, H-2´), 5.13 (dd, *J* = 10.3, 3.3 Hz, 1H, H-3´), 4.84 (dd, *J* = 6.0, 3.6 Hz, 1H, H-1´), 4.18 – 4.03 (m, 3H, H-6a´, H-6b´, H-5´), 2.95 (dt, *J* = 18.0, 4.7 Hz, 2H, CH_2-3), 2.16 (s, 3H, CH_3CO), 2.06 (bs, 6H, 2 × CH_3CO), 2.03 (s, 3H, CH_3CO); ^{13}C NMR (100 MHz, $CDCl_3$) δ 170.5,

170.1, 170.0, 169.8 (4 × CH_3*C*O), 130.1 (C-1), 123.8 (t, *J* = 4.3, C-2), 72.3 (C-1´), 68.7 (C-5´), 68.2, 68.0, 67.7 (C-3´, C-2´, C-4´), 62.0 (C-6´), 35.0 (t, *J* = 22.6 Hz, C-3), 20.7, 20.6, 20.6, 20.6 (4 × C*H*3CO); ^{19}F NMR (377 MHz, $CDCl_3$) δ -80.82 (tt, *J* = 9.7, 2.8, 3F), -112.466 -113.580 (m, 2F), -121.94 (bs, 2F), -122.9 -123.20 (m, 4F), -126.16 (tdd, *J* = 14.9, 7.4, 3.6 Hz, 2F); IR ($CDCl_3$, cm^{-1}): 2960, 1749, 1371, 1348, 1242, 1242, 1233, 1167, 1145, 1122, 1083, 1057, 970, 603, 564, 533; MS (ESI+, m/z (rel.%)): 713.4 (100); HRMS (ESI) calcd. for $C_{23}H_{23}O_9F_{13}Na$: 713.10267, found 713.10254.

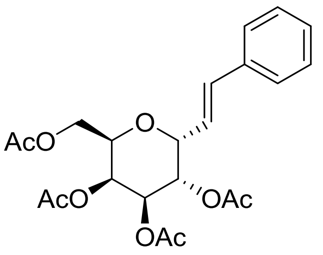

(1*E*)-2-Phenyl-1-(2´,3´,4´,6´-tetra-*O*-acetyl-α-D-galactopyranosyl)ethene (9d). Yield: 82%; a colorless oil; R_f = 0.4 (3/1 hexane/EtOAc); $[\alpha]_D$ = 129.7° (c = 0.410 g/ml, $CHCl_3$); ^{1}H NMR (600 MHz, $CDCl_3$) δ 7.41 (dd, *J* = 8.2, 1.2 Hz, 2H, H-2´´, H-6´´), 7.35 (ddd, *J* = 7.6, 6.8, 1.2 Hz, 2H, H-3´´, H-5´´), 7.30 (m, 1H, H-4´´), 6.77 (dd, *J* = 16.1, 1.7 Hz, 1H, H-2), 6.32 (dd, *J* = 16.1, 5.7 Hz, 1H, H-1), 5.44 (dd, *J* = 3.4, 1.8 Hz, 1H, H-4´), 5.40 (dd, *J* = 10.5, 6.0 Hz, 1H, H-2´), 5.22 (dd, *J* = 10.5, 3.3 Hz, 1H, H-3´), 4.97 (dt, *J* = 5.8, 1.8 Hz, 1H, H-1´), 4.25 (dd, *J* = 6.4, 1.7 Hz, 1H, H-5´), 4.16 (dd, *J* = 11.4, 6.8 Hz, 1H, H-6a´), 4.11 (dd, *J* = 11.4, 6.1 Hz, 1H, H-6b´), 2.16 (s, 3H, C*H*3CO), 2.06 (s, 3H, C*H*3CO), 2.05 (s, 3H, C*H*3CO), 2.02 (s, 3H, C*H*3CO); ^{13}C NMR (151 MHz, $CDCl_3$) δ 170.5, 170.2, 170.1, 170.0 (4 × CH_3*C*O), 136.1 (C-1´´), 135.9 (C-2´), 128.7 (C-3´´, C-5´´), 128.4 (C-4´´), 126.6 (C-2´´, C-6´´), 120.6 (C-1), 73.2 (C-1´), 68.4 (C-5´), 68.4 (C-3´), 68.1 (C-2´, C-4´), 61.9 (C-6´), 20.8, 20.7, 20.69, 20.67 (4 × *C*H3CO); IR ($CHCl_3$, cm^{-1}): 3085, 3064, 3030, 1747, 1653, 1647, 1600, 1578, 1496, 1449, 1371, 1232, 1164, 1083, 1056, 1030, 965, 694, 601; MS (ESI+, m/z (rel.%)): 457.2 (100); HRMS (ESI) calcd. for $C_{22}H_{26}O_9Na$: 457.14690, found 457.14679.

(1*E*)-2-(4´´-Fluorophenyl)-1-(2´,3´,4´,6´-tetra-*O*-acetyl-α-D-galactopyranosyl)ethene (9e).

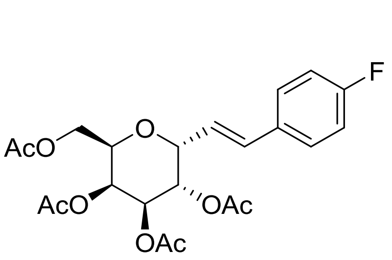

Yield: 79%; a colorless oil; R_f = 0.4 (3/1 hexane/EtOAc); $[\alpha]_D$ = 140.3° (c = 0.258 g/ml, $CHCl_3$); ^{1}H NMR (600 MHz, $CDCl_3$): δ 7.37 (m, 2H, H-2´´, H-6´´), 7.04 (m, 2H, H-3´´, H-5´´), 6.73 (dd, *J* = 16.1, 1.8 Hz, 1H, H-2), 6.23 (dd, *J* = 16.1, 5.6 Hz, 1H, H-1), 5.44 (dd, *J* = 3.4, 1.8 Hz, 1H, H-4´), 5.39 (dd, *J* = 10.5, 5.9 Hz, 1H, H-2´), 5.21 (dd, *J* = 10.5, 3.3 Hz, 1H, H-3´), 4.95 (dt, *J* = 5.8, 1.9 Hz, 1H, H-1´), 4.23 (ddd, *J* = 6.4, 1.8 Hz, 1H, H-5´), 4.17 (dd, *J* = 11.3, 6.8 Hz, 1H, H-6a´), 4.11 (dd, *J* = 11.3, 6.0 Hz, 1H, H-6b´), 2.16 (s, 3H, C*H*3CO), 2.06 (s, 3H, C*H*3CO), 2.05 (s,

3H, C*H*$_3$CO), 2.03 (s, 3H, C*H*$_3$CO). ^{13}C NMR (151 MHz, CDCl$_3$) δ 170.6, 170.4, 170.3, 170.0 (4 × CH$_3$*C*O), 162.9 (d, *J* = 248.2 Hz, C-4´´), 162.1 (C-2), 135.0 (C-1´´), 132.1 (d, *J* = 3.4 Hz, C-1´´), 128.2 (d, *J* = 8.2 Hz, C-2´´, C-6´´), 120.4 (d, *J* = 2.3 Hz, C-1), 115.7 (d, *J* = 21.6 Hz, C-3´´, C-5´´), 73.2 (C-1´), 68.6 (C-3´, C-5´), 68.2 (C-2´), 68.2 (C-4´), 62.0 (C-6´), 21.0, 20.9, 20.9, 20.8 (4 × C*H*$_3$CO); ^{19}F NMR (376 MHz, CDCl$_3$) δ -113.04 (m, 1F); IR (CDCl$_3$, cm^{-1}): 1749, 1653, 1603, 1510, 1371, 1233, 1158, 1056, 966, 853; MS (ESI+, m/z (rel.%)): 475.3 (100); HRMS (ESI) calcd. for $C_{22}H_{25}O_9FNa$: 475.13748, found 475.13735.

(1*E*)-2-(4´´-Trifluoromethylphenyl)-1-(2´,3´,4´,6´-tetra-*O*-acetyl-α-D-galactopyranosyl)-ethene (9f). Yield: 78%; a colorless foam; R_f = 0.4 (3/1 hexane/EtOAc); $[\alpha]_D$ = 131.3° (c = 0.498 g/ml, CHCl$_3$); ^{1}H NMR (600 MHz, CDCl$_3$) δ 7.60 (d, *J* = 8.2 Hz, 2H, H-2´´, H-6´´), 7.50 (d, *J* = 8.2 Hz, 2H, H-3´´, H-5´´), 6.80 (dd, *J* = 16.2, 1.7 Hz, 1H, H-2), 6.40 (dd, *J* = 16.1, 5.4 Hz, 1H, H-1), 5.45 (dd, *J* = 3.3, 1.8 Hz, 1H, H-4´), 5.41 (dd, *J* = 10.3, 5.9 Hz, 1H, H-2´), 5.20 (dd, *J* = 10.3, 3.3 Hz, 1H, H-3´), 4.98 (td, *J* = 5.7, 1.7 Hz, 1H, H-1´), 4.25 – 4.18 (m, 2H, H-5´, H-6a´´), 4.11 (dd, *J* = 10.8, 5.4 Hz, 1H, H-6b´), 2.16 (s, 3H, C*H*$_3$CO), 2.07 (s, 3H, C*H*$_3$CO), 2.06 (s, 3H, C*H*$_3$CO), 2.03 (s, 3H, C*H*$_3$CO); ^{13}C NMR (151 MHz, CDCl$_3$) δ 170.5, 170.15, 170.1, 169.8 (4 × CH$_3$*C*O), 139.3 (C-1´´), 134.3 (C-2), 130.2 (q, *J* = 32.5 Hz, C-4´´), 126.8 (C-2´´, C-6´´), 124.9 (q, *J* = 273.1, *C*F$_3$), 125.7 (d, *J* = 3.9 Hz, C-3´´, C-5´´). 123.7 (C-1), 72.8 (C-1´), 68.7 (C-5´), 68.4 (C-3´), 68.1 (C-2´), 68.0 (C-4´), 61.8 (C-6´), 20.8, 20.7, 20.7, 20.7 (4 × *C*H$_3$CO); ^{19}F NMR (376 MHz, CDCl$_3$) δ -62.62 (s, CF$_3$); IR (CDCl$_3$, cm^{-1}): 1747, 1650, 1617, 1579, 1510, 1415, 1371, 1326, 1234, 1169, 1130, 1110, 1068, 1058, 1017, 969, 830, 600, 504; MS (ESI+, m/z (rel.%)): 525.3 (100); HRMS (ESI) calcd. for $C_{23}H_{25}O_9F_3Na$: 525.13429, found 525.13422.

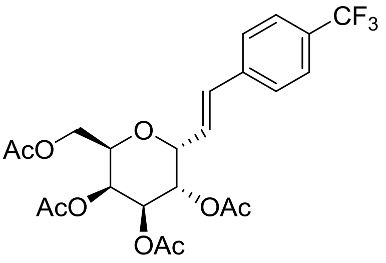

VII Deprotection of 9.

(1*E*)-1-(α-D-Galactopyranosyl)hept-1-ene (10b). Into a solution of compound **9b** (70 mg; 0.16 mmol) in the 9:1 mixture of MeOH with H_2O (5 mL) was added K_2CO_3 (113 mg; 0.82 mmol). The formed reaction mixture was then stirred at room temperature for 16 h. All volatiles were removed in vacuo and column chromatography of the residue on silica gel (CH_2Cl_2/MeOH 12:1) afforded 37.4 mg (88%) of compound **10b** as a white solid. R_f = 0.25 (CH_2Cl_2/MeOH 10/1); m.p. 89-90°C (*i*PrOH); $[\alpha]_D$ = 115.6° (c = 0.198 g/ml, MeOH); ^{1}H NMR (400 MHz, MeOD) δ 5.86 (ddd, *J* = 15.4, 6.7, 1.4 Hz, 1H, H-2), 5.74 (dd, *J* = 15.7, 5.4 Hz, 1H, H-1), 4.43 (t, *J* = 5.7 Hz, 1H, H-1´), 3.95 (dd, J = 9.7, 5.9 Hz, 1H, H-2´), 3.88 (dd, *J* = 3.2, 1.8 Hz, 1H, H-4´), 3.80 (td, *J* = 6.0, 5.3, 1.5 Hz, 1H, H-5´), 3.72 (dd, *J* = 11.3, 6.6 Hz, 1H, H-6a´), 3.66 (dd, *J* = 11.3, 5.2 Hz, 1H, H-6b´), 3.56 (dd, *J* = 9.7, 3.3 Hz, 1H, H-3´), 2.10 (q, *J* = 7.0 Hz, 2H, CH_2-3), 1.47 – 1.37 (m, 2H, CH_2-4), 1.35 – 1.27 (m, 4H, CH_2-5,6), 0.90 (t, *J* = 6.8 Hz, 3H, CH_3-7); ^{13}C NMR (100 MHz, MeOD) δ 137.1 (C-2), 124.7 (C-1), 76.9 (C-1´), 74.2 (C-5´), 72.4 (C-3´), 71.0 (C-4´), 70.0 (C-2´), 62.7 (C-6´), 33.8 (*C*H_2-3), 30.0 (*C*H_2-4), 32.6, 23.6 (*C*H_2-5,6), 14.4 (*C*H_3-7); IR (KBr, cm^{-1}): 3396, 3040, 2954, 2922, 2872, 2854, 1663, 1467, 1455, 1433, 1385, 1316, 1086, 1064, 1038, 982, 973. MS (ESI+, m/z (rel.%)): 283.1 (100); HRMS (ESI) calcd. for $C_{13}H_{24}O_5Na$: 283.15160, found 283.15160.

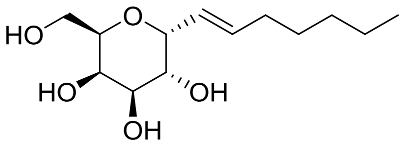

(1*E*)-1-(α-D-Galactopyranosyl)-4,4,5,5,6,6,7,7,8,8,9,9,9-tridecafluoronon-1-ene (10c). Into a solution of compound **9c** (80 mg; 0.12 mmol) in the 9:1 mixture of MeOH with H_2O (6 mL) was added K_2CO_3 (80 mg; 0.58 mmol). The formed reaction mixture was then stirred at room temperature for 16 h. All volatiles were removed in vacuo and column chromatography of the residue on silica gel (CH_2Cl_2/MeOH 12:1) afforded 48.4 mg (80%) of compound **10c** as a white foam. R_f = 0.35 (CH_2Cl_2/MeOH 10/1); $[\alpha]_D$ = 72.3° (c = 0.278 g/ml, MeOH); ^{1}H NMR (401 MHz, MeOD) δ 6.11 (dd, *J* = 15.8, 4.4 Hz, 1H, H-1), 5.89 (ddd, *J* = 14.1, 7.1, 3.5 Hz, 1H, H-2), 4.53 (dd, *J* = 6.1, 4.4, 1H, H-1´), 4.00 (dd, *J* = 9.7, 6.1 Hz, 1H, H-2´), 3.88 (dd, *J* = 3.2, 1.6 Hz, 1H, H-4´), 3.78 (m, 2H, H-5´, H-6a´), 3.67 (dd, *J* = 10.9, 4.4 Hz, 1H, H-6b´´), 3.51 (dd, *J* = 9.7, 3.2 Hz, 1H, H-3´), 3.02 (dd, *J* = 18.7, 7.2 Hz, 2H, H-3a, H-3b); ^{13}C NMR (100 MHz, MeOD) δ 133.9 (C-1), 121.8 (t, *J* = 5.4 Hz, C-2), 76.2 (C-1´), 74.6 (C-5´), 72.4 (C-3´), 70.9 (C-4´), 69.7 (C-2´), 62.7 (C-6´), 35.6 (t, *J* = 22.5 Hz, C-3); ^{19}F NMR (377 MHz, MeOD) δ -82.42 (tt, *J* = 10.2, 2,7 Hz, 3F), -114.03 (m, 2F), -122.95 (m, 2F), -123.98 (m, 4F), -127.36 (m, 2F); IR (KBr, cm^{-1}): 3356, 2924, 2855, 1432, 1353, 1318, 1238, 1145, 1122,

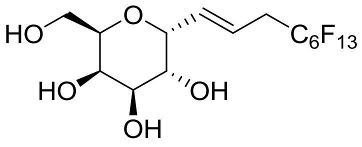

1079, 983, 566, 531; MS (ESI+, m/z (rel.%)): 713.4 (100); HRMS (ESI) calcd. for $C_{23}H_{23}O_9F_{13}Na$: 713.10267, found 713.10254.

(1*E*)-2-Phenyl-1-(α-D-galactopyranosyl)ethene (10d). Into a solution of compound **9d** (0.1 g; 0.23 mmol) in a 9:1 mixture of MeOH with H_2O (5 mL) was added K_2CO_3 (73 mg; 0.53 mmol). The formed reaction mixture was then stirred at room temperature for 16 h. All volatiles were removed in vacuo and column chromatography of the residue on silica gel (CH_2Cl_2/MeOH 12:1) afforded 57 mg (93%) of compound **10d** as white crystals. R_f = 0.3 (10/1 CH_2Cl_2/MeOH), $[\alpha]_D$ = 109.6° (c = 0.427 g/ml, MeOH); m.p. 156-157 °C (*i*PrOH); ^{1}H NMR (600 MHz, MeOD) δ 7.45 (m, *J* = 7.3 Hz, 2H, H-2´´, 6´´), 7.32 (m, *J* = 7.7 Hz, 2H, H-3´´, 5´´), 7.24 (m, *J* = 7.4 Hz, 1H, H-4´´), 6.80 (dd, *J* = 16.3, 1.8 Hz, 1H, H-2), 6.58 (dd, *J* = 16.3, 5.1 Hz, 1H, H-1), 4.68 (td, *J* = 5.9, 1.8 Hz, 1H, H-1´), 4.08 (dd, *J* = 9.7, 6.0 Hz, 1H, H-2´), 3.93 (dd, *J* = 3.2, 1.8 Hz, 1H, H-4´), 3.91 (ddd, *J* = 6.7, 4.8, 1.7 Hz, 1H, H-5´), 3.82 (dd, *J* = 11.5, 7.0 Hz, 1H, H-6a´), 3.72 (dd, *J* = 11.5, 4.8 Hz, 1H, H-6b´), 3.64 (dd, *J* = 9.7, 3.3 Hz, 1H, H-3´); ^{13}C NMR (150 MHz, MeOD) δ 138.4 (C-4´´), 135.0 (C-2), 129.6 (C-3´´, C-5´´), 128.6 (C-4´´), 127.5 (C-2´´, C-6´´), 124.7 (C-1), 76.9 (C-1´), 74.6 (C-5´), 72.6 (C-3´), 71.0 (C-4´), 70.2 (C-2´), 62.8 (C-6´); IR (KBr, cm^{-1}): 3414, 1645, 1599, 1577, 1495, 1328, 1205, 1080, 1056, 1016, 975, 975, 749, 700, 530; MS (ESI+, m/z (rel.%)): 289.2 (100); HRMS (ESI) calcd. for $C_{14}H_{18}O_5Na$: 289.10464, found 289.10467.

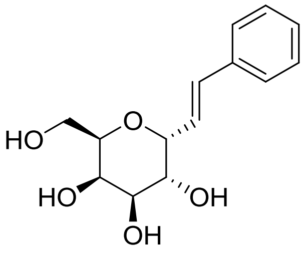

(1*E*)-2-(4´´-Fluorophenyl)-1-(α-D-galactopyranosyl)ethene (10e). Into a solution of compound **9e** (60 mg; 0.13 mmol) in a 9:1 mixture of MeOH with H_2O (5 mL) was added K_2CO_3 (42 mg; 0.31 mmol). The formed reaction mixture was then stirred at room temperature for 16 h. All volatiles were removed in vacuo and column chromatography of the residue on silica gel (12:1 CH_2Cl_2/MeOH) afforded 34 mg (90%) of compound **10e** as white crystals. R_f = 0.3 (10/1 CH_2Cl_2/MeOH); m.p. 145-146 °C (*i*PrOH); $[\alpha]_D$ = 89.7° (c = 0.243 g/ml, MeOH); ^{1}H NMR (600 MHz, MeOD) δ 7.45 (m, 2H, H-6´´, H-2´´), 7.04 (m, 2H, H-3´´, H-5´´), 6.76 (dd, *J* = 16.3, 1.8 Hz, 1H, H-2), 6.50 (dd, *J* = 16.3, 5.1 Hz, 1H, H-1), 4.64 (td, *J* = 5.8, 1.8 Hz, 1H, H-1´), 4.05 (dd, *J* = 9.7, 6.0 Hz, 1H, H-2´), 3.91 (dd, *J* = 3.2, 1.8 Hz, 1H, H-4´), 3.87 (ddd, *J* = 6.7, 4.7, 1.8 Hz, 1H, H-5´), 3.79 (dd, *J* = 11.5, 7.0 Hz, 1H, H-6a´), 3.70 (dd, *J* = 11.5, 4.7 Hz, 1H, H-6b´), 3.61 (dd, *J* = 9.7, 3.3 Hz, 1H, H-3´); ^{13}C NMR (151 MHz, MeOD) δ 163.6 (d, *J* = 245.3 Hz, C-4´´), 134.9 (d, *J* = 3.2 Hz, C-

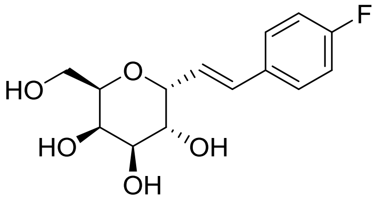

1´´), 133.7 (C-2), 129.2 (d, J = 8.0 Hz, C-2´´, C-6´´), 124.7 (C-1), 116.3 (d, J = 21.8 Hz, C-3´´, C-5´´), 116.2, 76.9 (C-1´), 74.6 (C-5´), 72.5 (C-3´), 71.0 (C-4´), 70.1 (C-2´), 62.8 (C-6´); ^{19}F NMR (376 MHz, MeOD) δ -116.93 (bs, 1F); IR (KBr, cm^{-1}): 3429, 1635, 1603, 1510, 1414, 1159, 1034, 970, 852, 181 cm^{-1}; MS (ESI+, m/z (rel.%)): 307.2 (100); HRMS (ESI) calcd. for $C_{14}H_{17}O_5FNa$: 307.09522, found 307.09521.

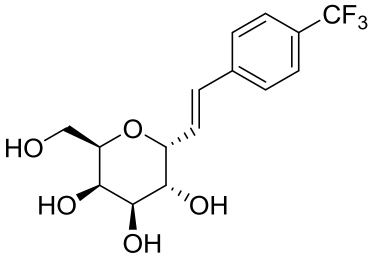

(1*E*)-2-(4´´-Trifluoromethylphenyl)-1-(α-D-galactopyranosyl)ethene (10f). Into a solution of compound **9f** (80 mg; 0.16 mmol) in a 9:1 mixture of MeOH with H_2O (5 mL) was added K_2CO_3 (51 mg; 0.37 mmol). The formed reaction mixture was then stirred at room temperature for 16 h. All volatiles were removed in vacuo and column chromatography of the residue on silica gel (12:1 CH_2Cl_2/MeOH) afforded 46 mg (87%) of compound **10f** as white crystals. R_f = 0.3 (10/1 CH_2Cl_2/MeOH); m.p. 176-177 °C (*i*PrOH); $[\alpha]_D$ = 79.7° (c = 0.212 g/ml, MeOH); ^{1}H NMR (401 MHz, MeOD) δ 7.61 (m, 4H, H-2´´, H-3´´, H-5´´, H-6´´), 6.86 (dd, J = 16.3, 1.9 Hz, 1H, H-2), 6.72 (dd, J = 16.3, 4.7 Hz, 1H, H-1), 4.69 (ddd, J = 6.3, 4.7, 1.9 Hz, 1H, H-1´), 4.08 (dd, J = 9.6, 6.0 Hz, 1H, H-2´), 3.91 (dd, J = 3.3, 1.8 Hz, 1H, H-4´), 3.88 (ddd, J = 6.5, 4.5, 1.8 Hz, 1H, H-5´), 3.81 (dd, J = 11.3, 7.0 Hz, 1H, H-6a´), 3.71 (dd, J = 11.4, 4.5 Hz, 1H, H-6b´), 3.60 (dd, J = 9.7, 3.2 Hz, 1H, H-3´); ^{13}C NMR (100 MHz, MeOD) δ 142.3 (C-1´´), 133.1 (C-2), 130.1 (q, J = 32.8 Hz, C-4´´), 128.3 (C-1), 127.9 (C-2´´, C-6´´, *C*F$_3$), 126.5 (d, J = 3.9 Hz, C-3´´, C-5´´); 76.7 (C-1´), 74.9 (C-5´), 72.6 (C-3´), 71.0 (C-4´), 70.1 (C-2´), 62.9 (C-6´); ^{19}F NMR (376 MHz, MeOD) δ -64.01 (bs, 3F); IR (KBr, cm^{-1}): 3498, 3408, 3350, 3055, 2954, 2923, 1614, 1576, 1516, 1414, 1332, 1287, 1163, 1119, 1110, 1081, 1068, 1061, 1060, 1016, 852, 735, 503; MS (ESI+, m/z (rel.%)): 357.1 (100); HRMS (ESI) calcd. for $C_{15}H_{17}O_5F_3Na$: 357.09203, found 357.09201.

VIII Copies of spectra

(1*E*)-[3,5-Bis-*O*-(4-toluoyl)-2-deoxy-α-D-ribofuranosyl]-{8,8'-μ-(propen-3-yl-disulfido)-[3,3'-como-cobalt(III)-bis-(1,2-dicarbaundecaborate)]} (α-4a).

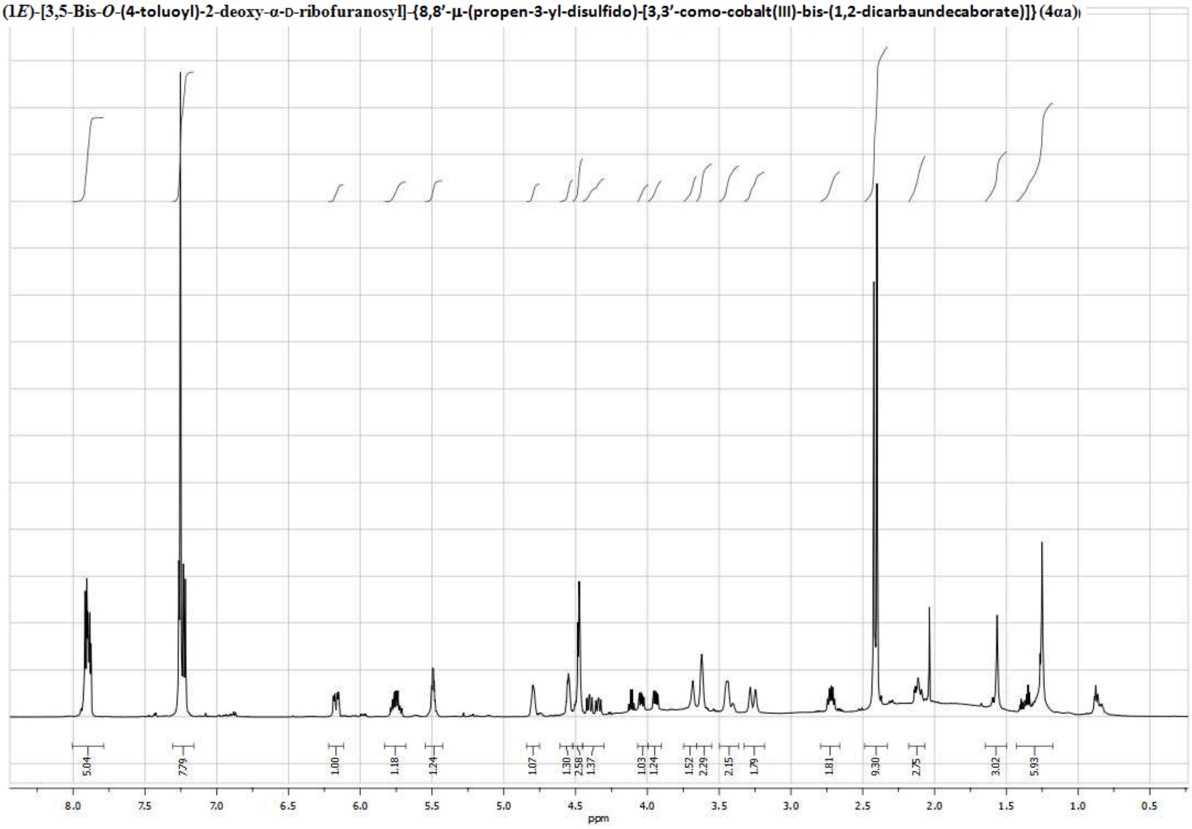

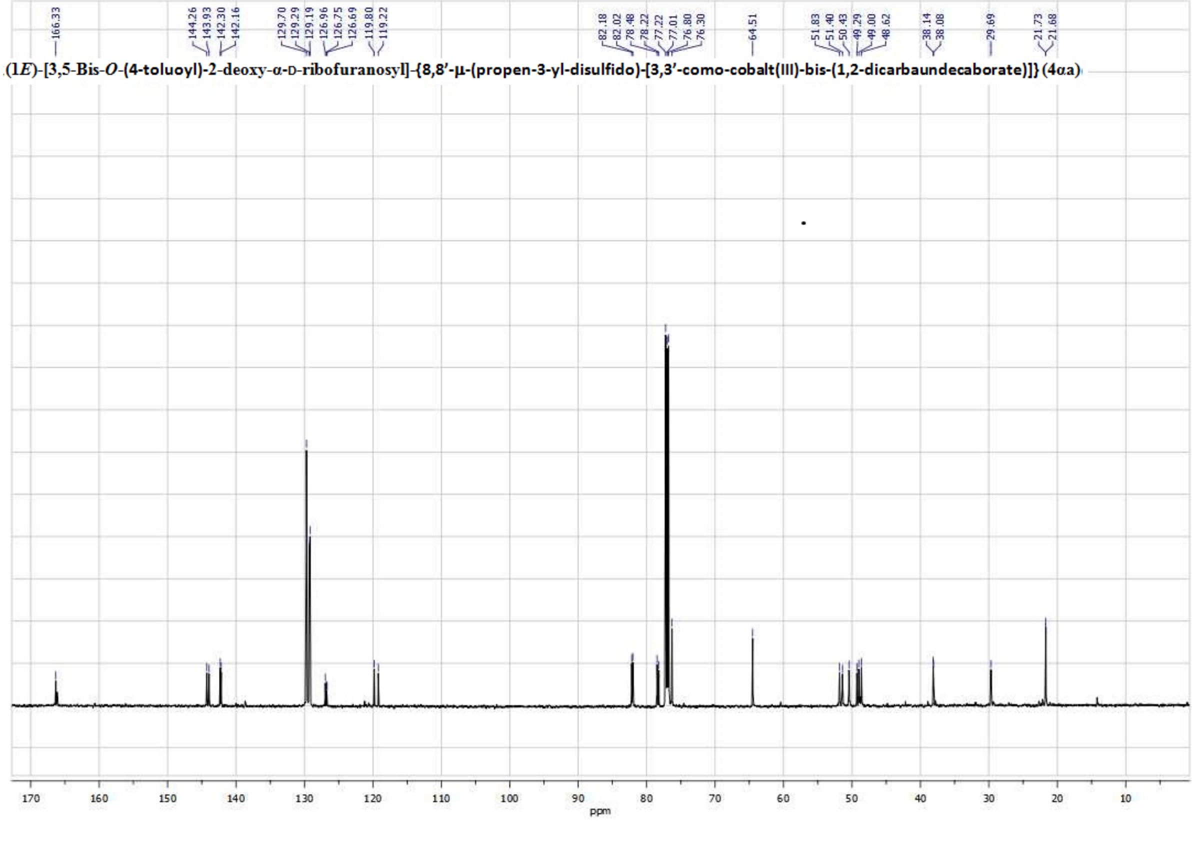

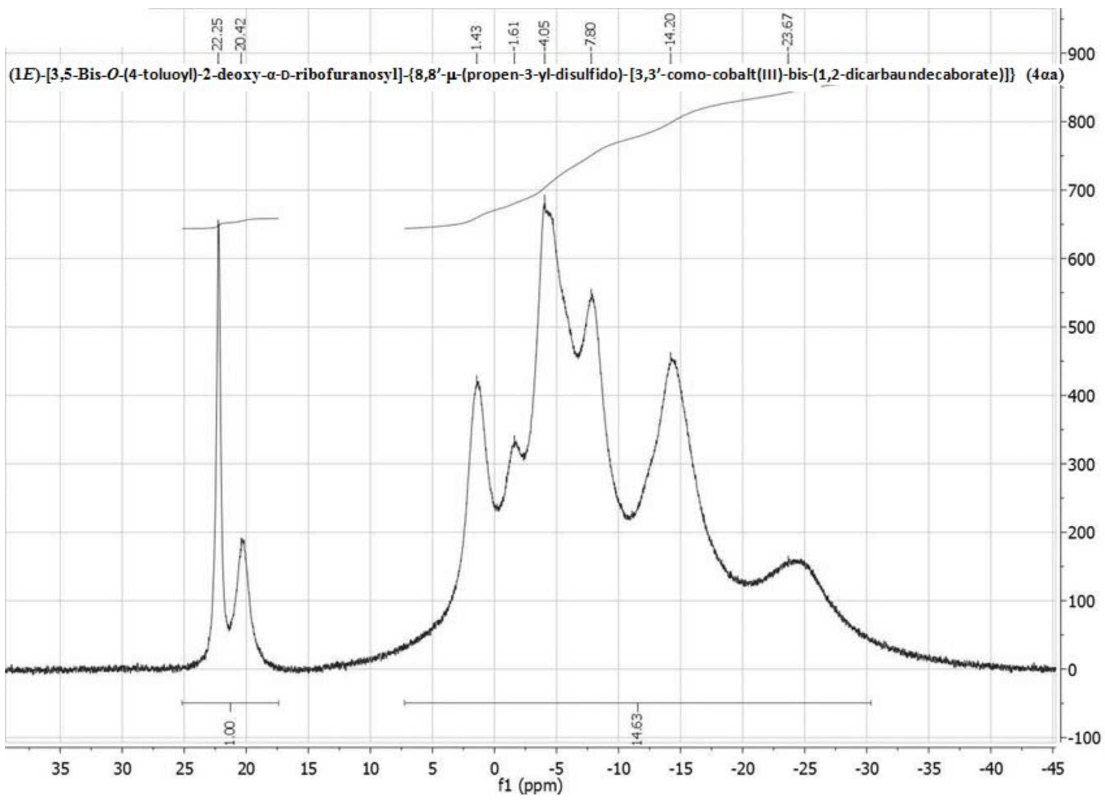
(1E)-[3,5-Bis-O-(4-toluoyl)-2-deoxy-α-D-ribofuranosyl]-{8,8′-μ-(propen-3-yl-disulfido)-[3,3′-como-cobalt(III)-bis-(1,2-dicarbaundecaborate)]} (4αa)
22.25
20.42
-1.43
-1.61
-4.05
-7.80
-14.20
-23.67
1.00
14.63
f1 (ppm)

(1*E*)-1-(3,5-Bis-*O*-(4-toluoyl)-2-deoxy-α-D-ribofuranosyl)hept-1-ene (α-4b).

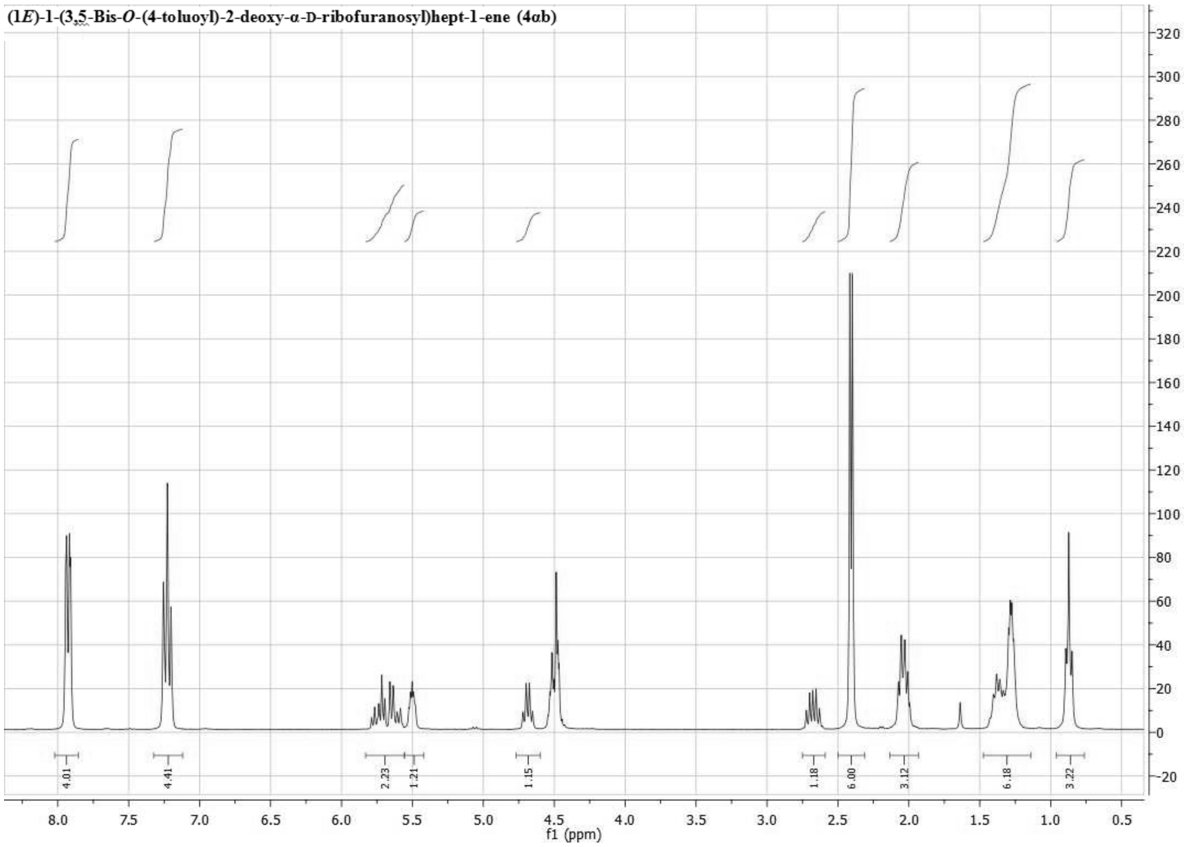

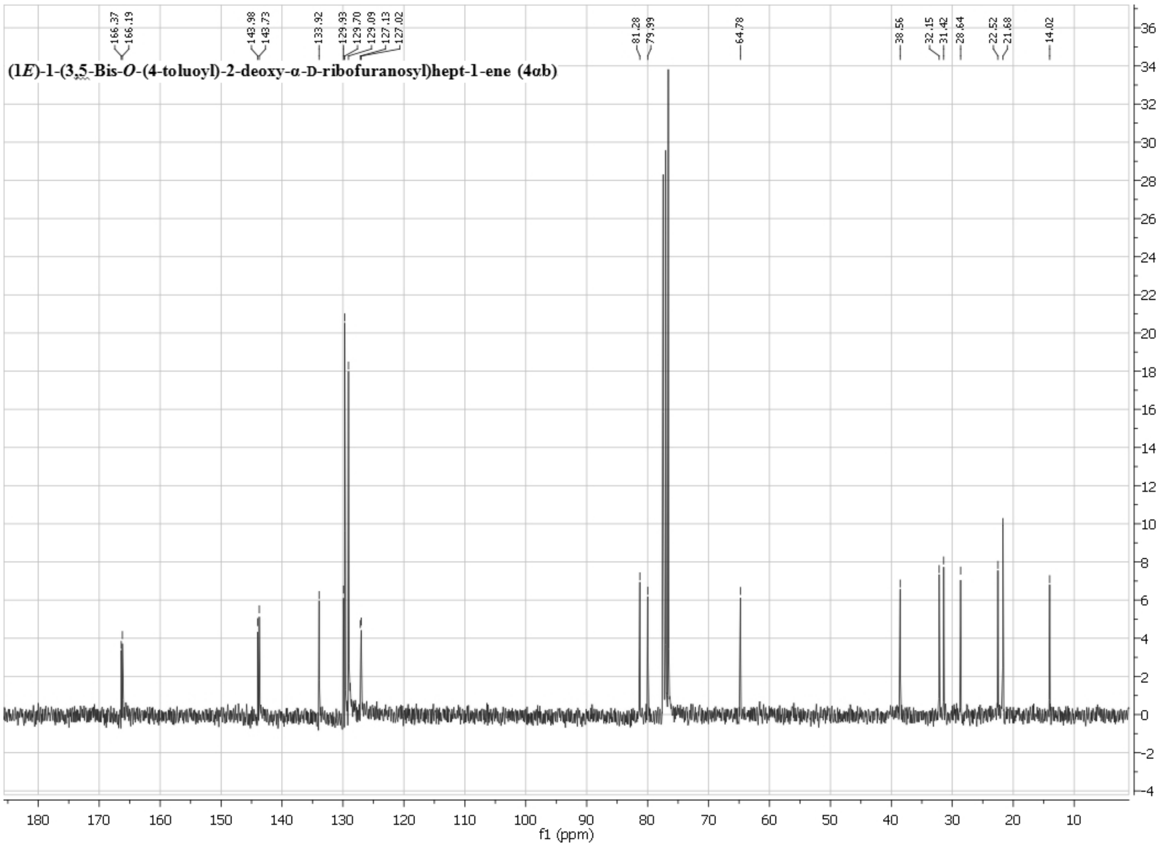

(1*E*)-1-(3,5-Bis-*O*-(4-toluoyl)-2-deoxy-α-D-ribofuranosyl)-4,4,5,5,6,6,7,7,8,8,9,9,9-trideca-fluoronon-1-ene (α-4c).

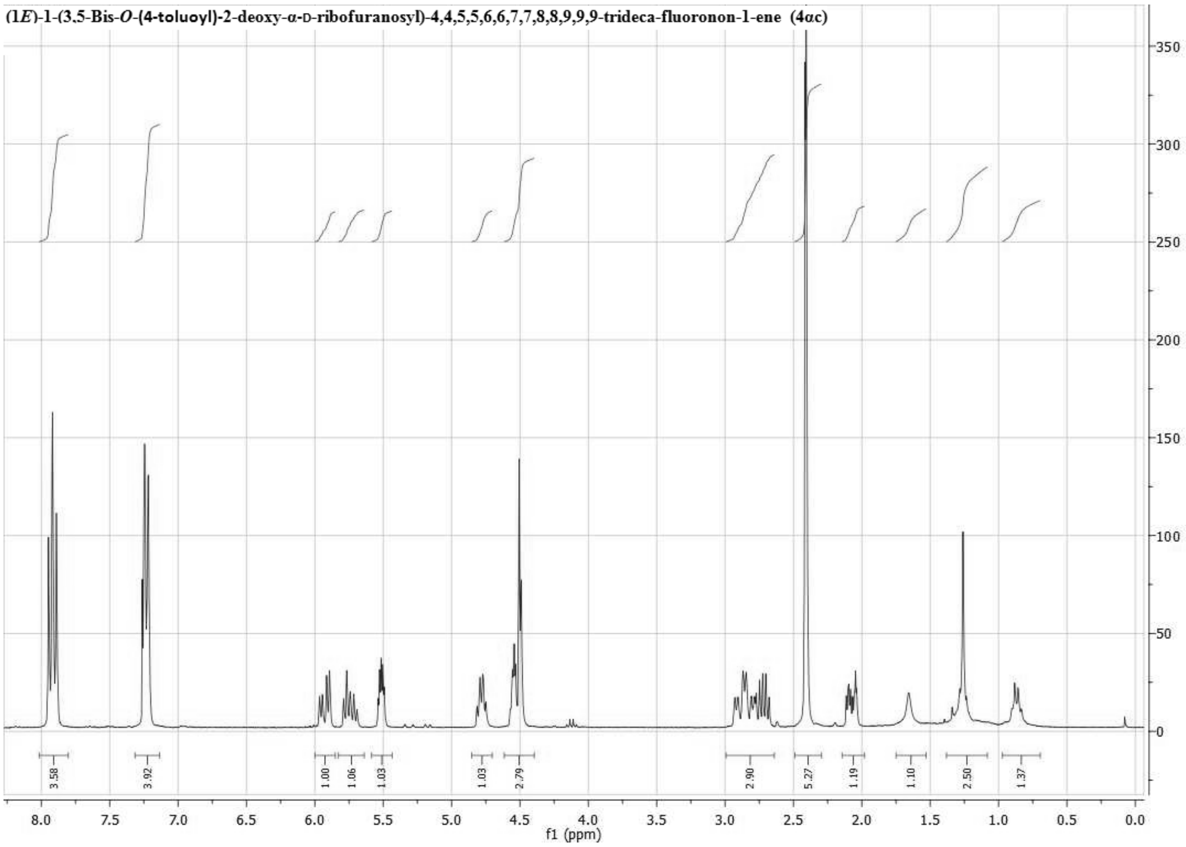

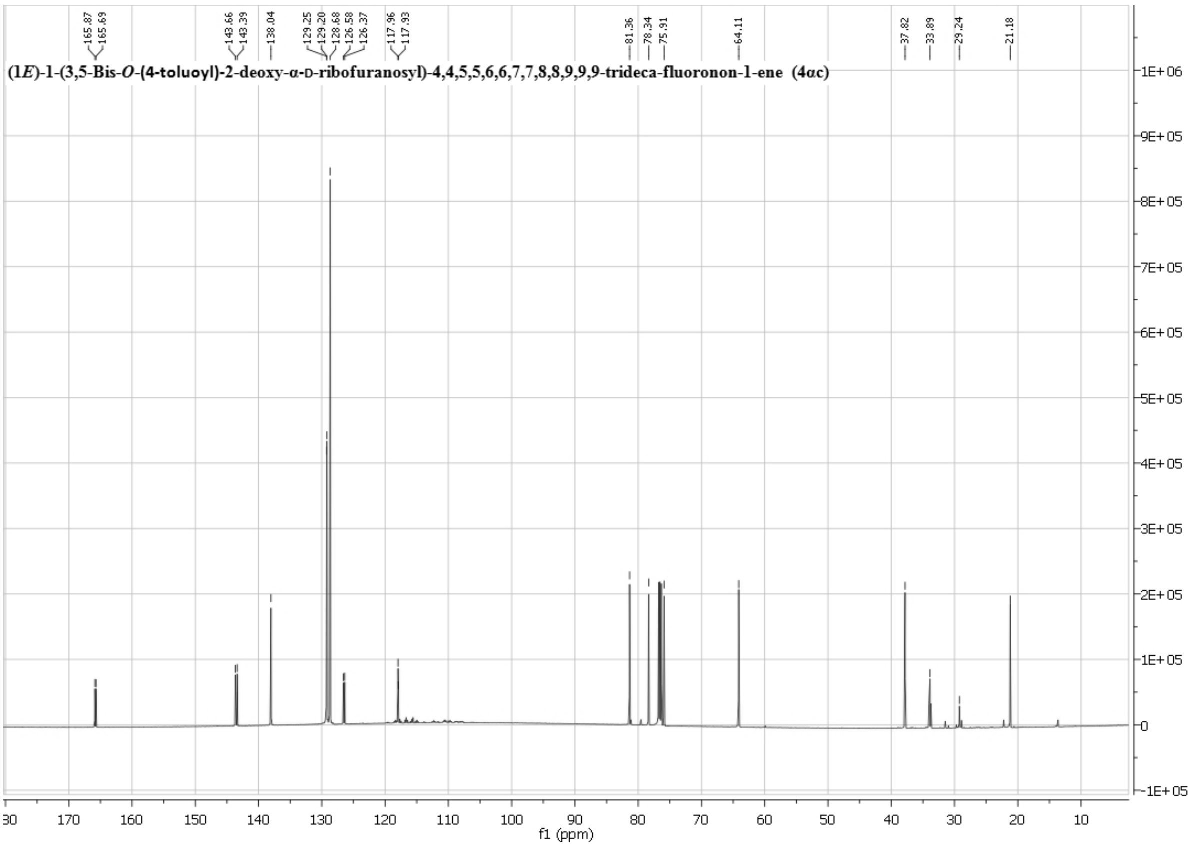

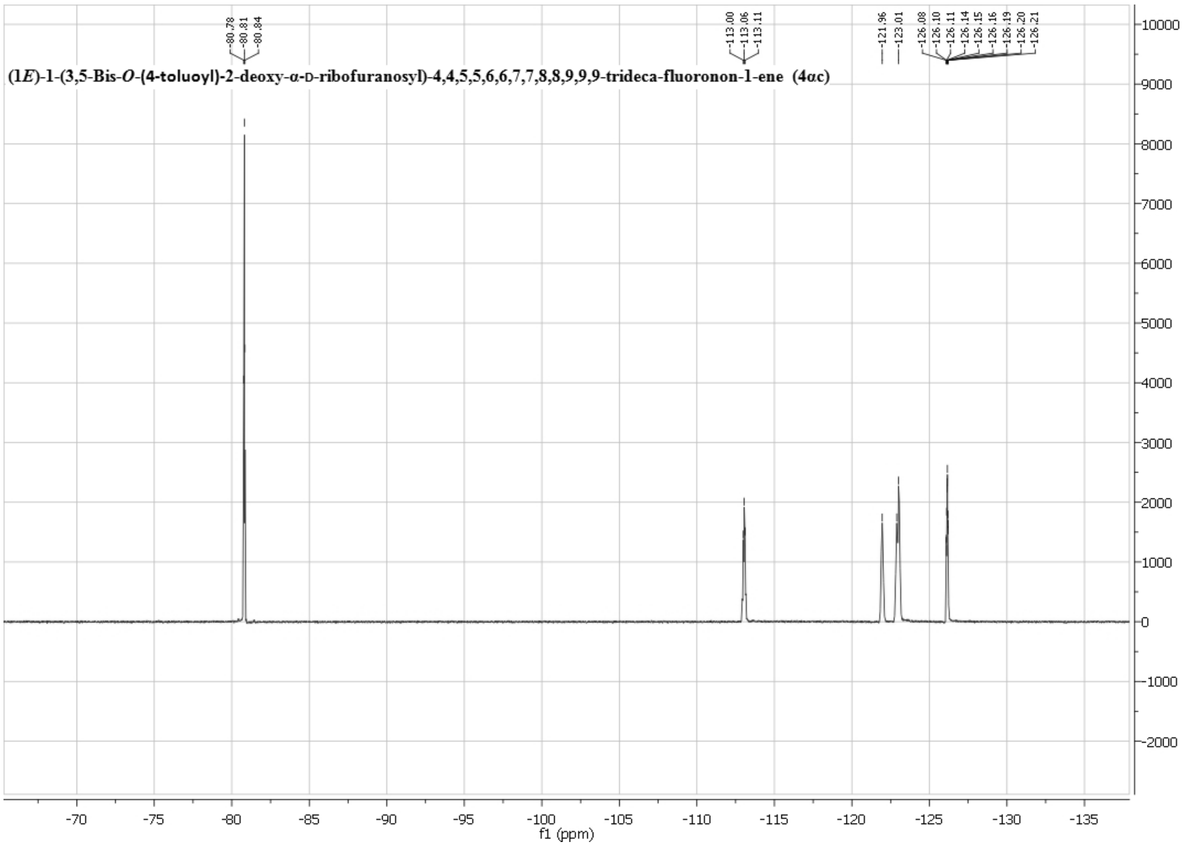
(1*E*)-1-(3,5-Bis-*O*-(4-toluoyl)-2-deoxy-α-D-ribofuranosyl)-4,4,5,5,6,6,7,7,8,8,9,9,9-trideca-fluoronon-1-ene (4αc)
-80.78
-80.81
-80.84
-113.00
-113.06
-113.11
-121.96
-123.01
-126.08
-126.10
-126.11
-126.14
-126.15
-126.16
-126.19
-126.20
-126.21
f1 (ppm)

(1*E*)-2-Phenyl-1-(3,5-bis-*O*-(4-toluoyl)-2-deoxy-α-D-ribofuranosyl)ethene (α-4d).

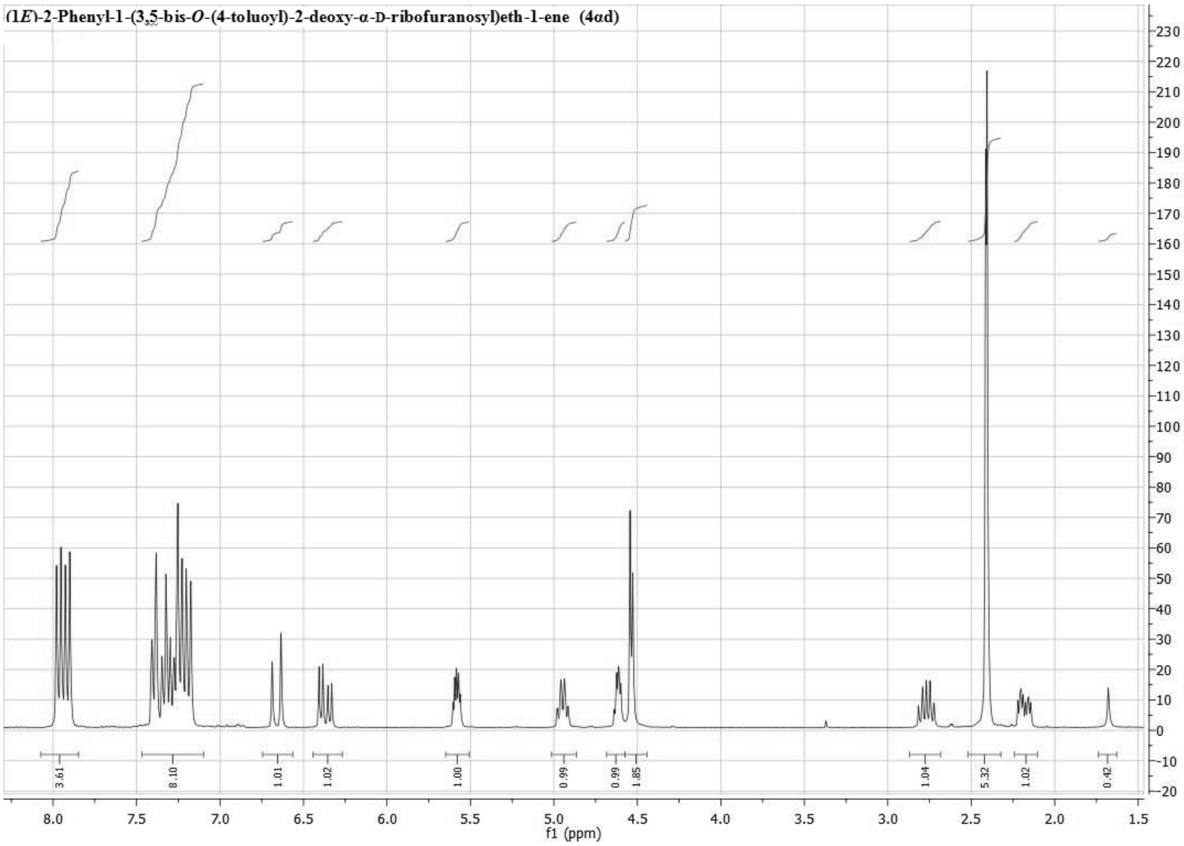

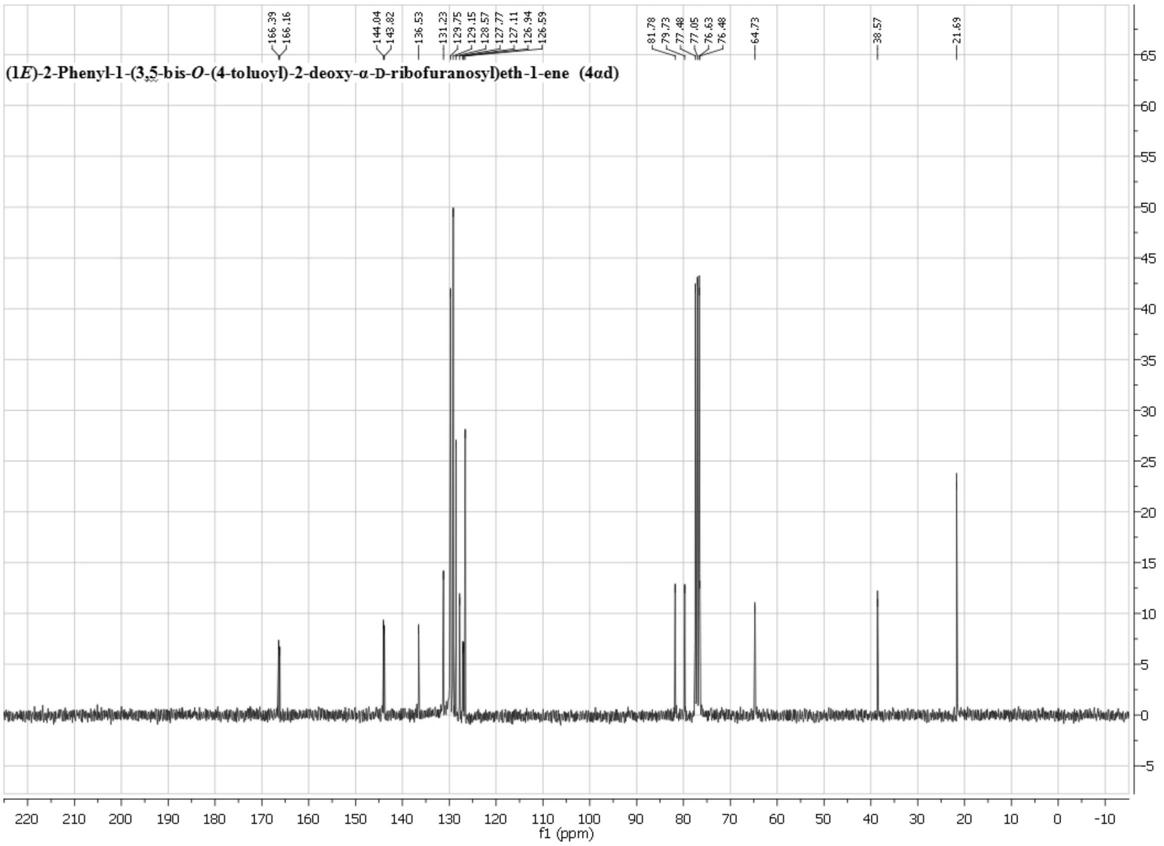

(1*E*)-2-(4-Fluorophenyl)-1-(3,5-bis-*O*-(4-toluoyl)-2-deoxy-α-D-ribofuranosyl)ethene (α-4e).

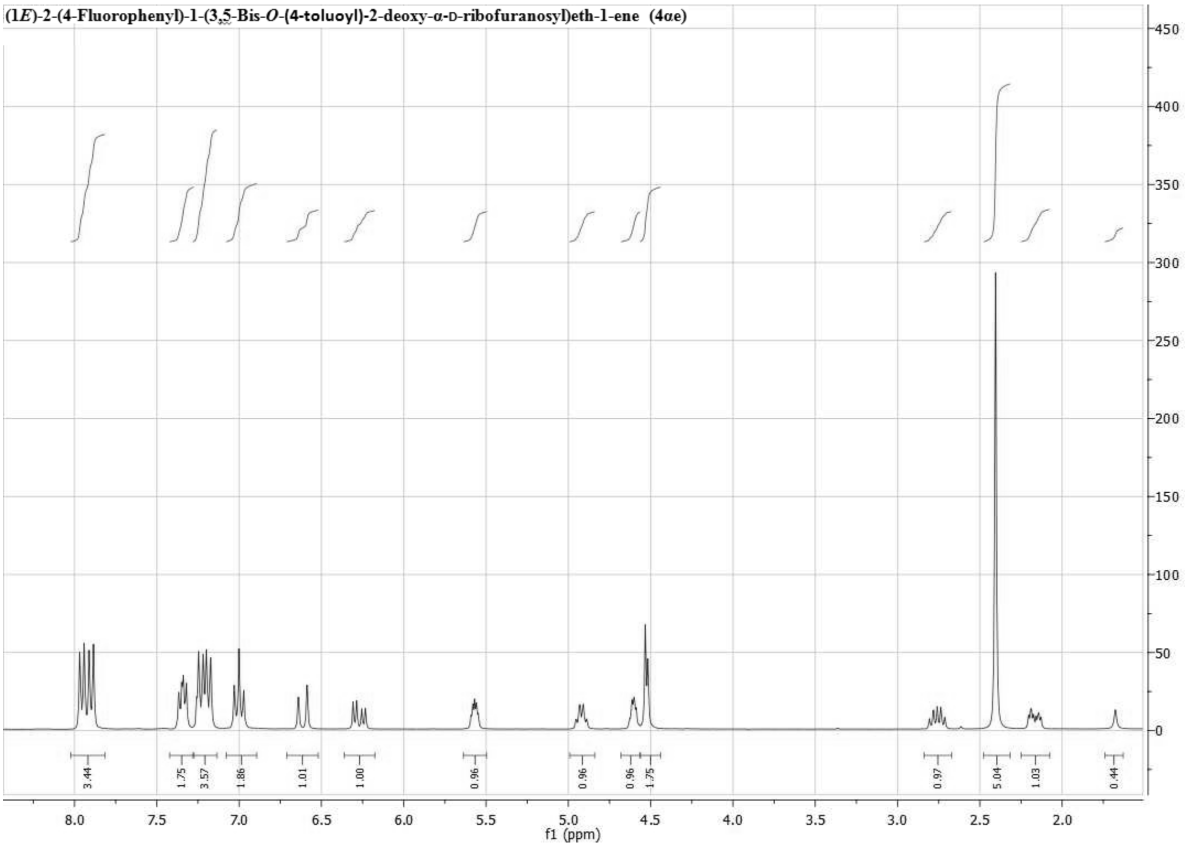

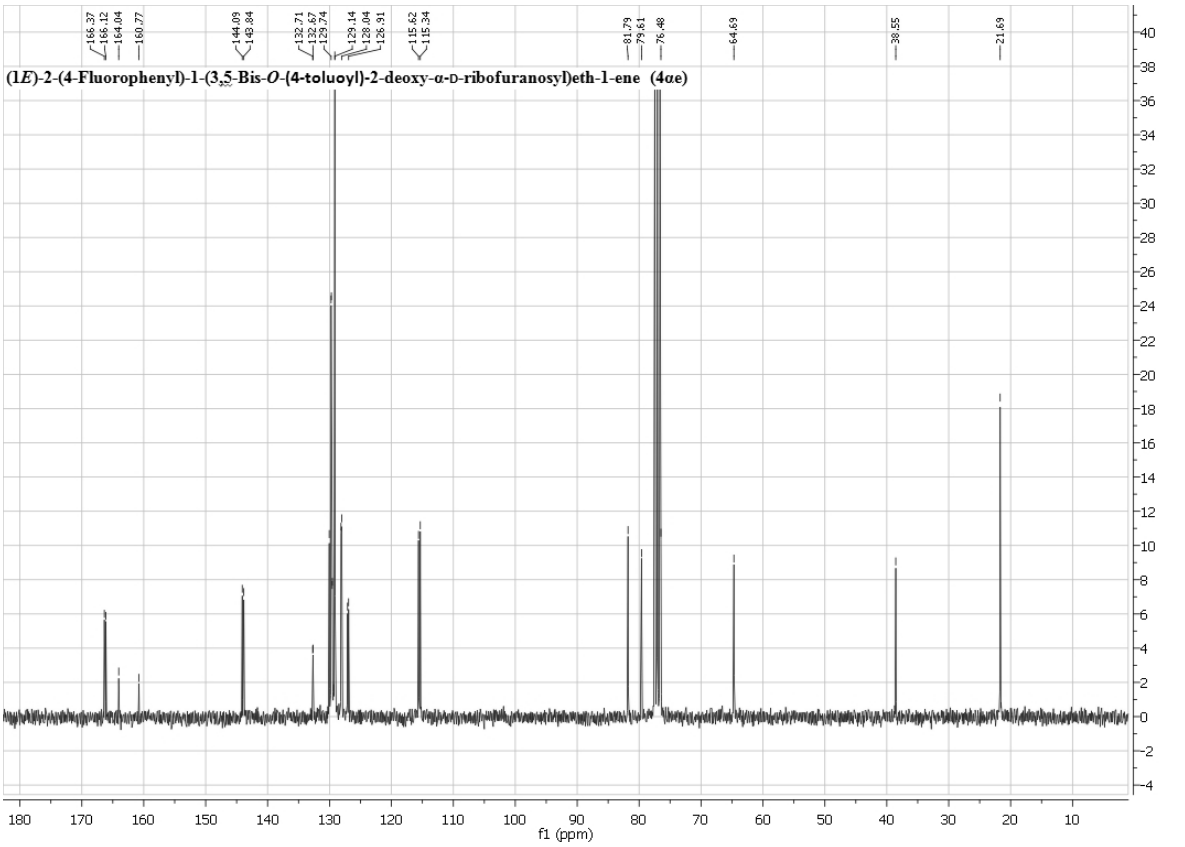

(1*E*)-2-(4-Fluorophenyl)-1-(3,5-Bis-*O*-(4-toluoyl)-2-deoxy-α-D-ribofuranosyl)eth-1-ene (4αe)

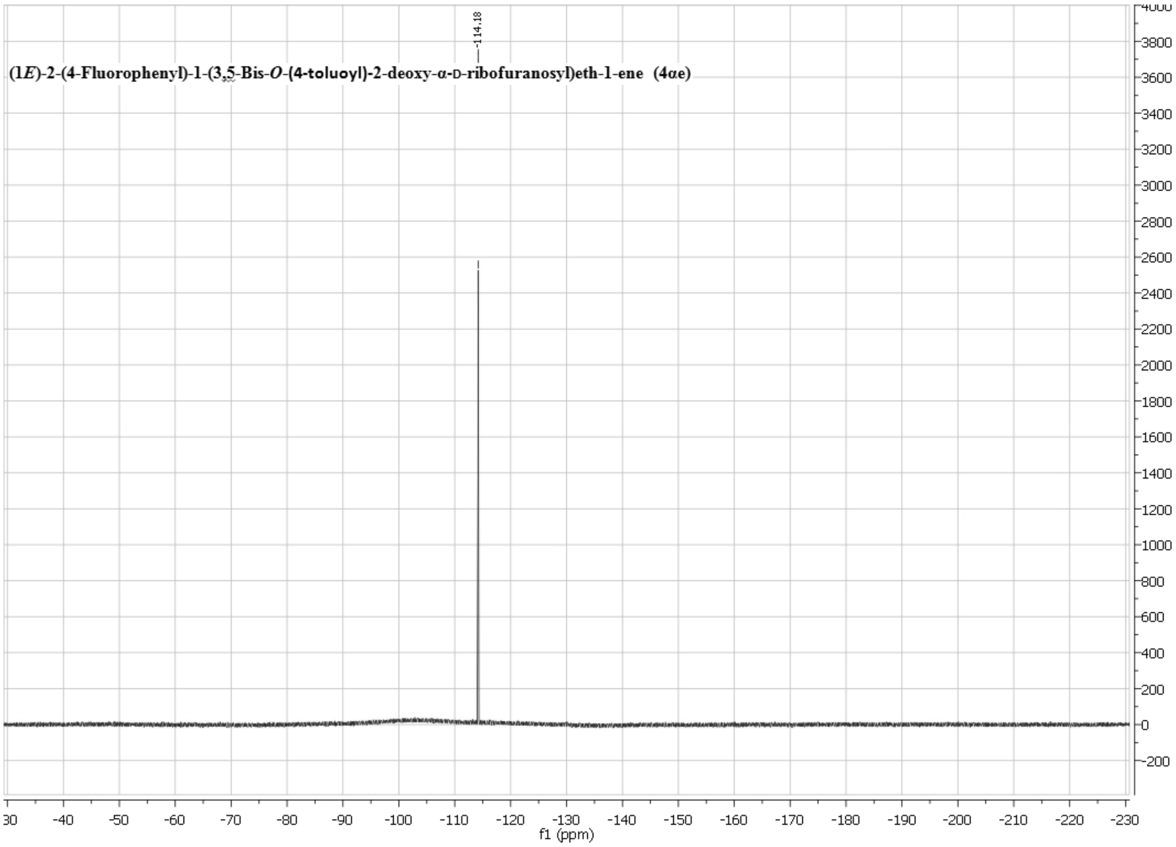

(1*E*)-2-(4-Trifluoromethylphenylphenyl)-1-(3,5-bis-*O*-(4-toluoyl)-2-deoxy-α-D-ribofura-nosyl)ethene (α-4f).

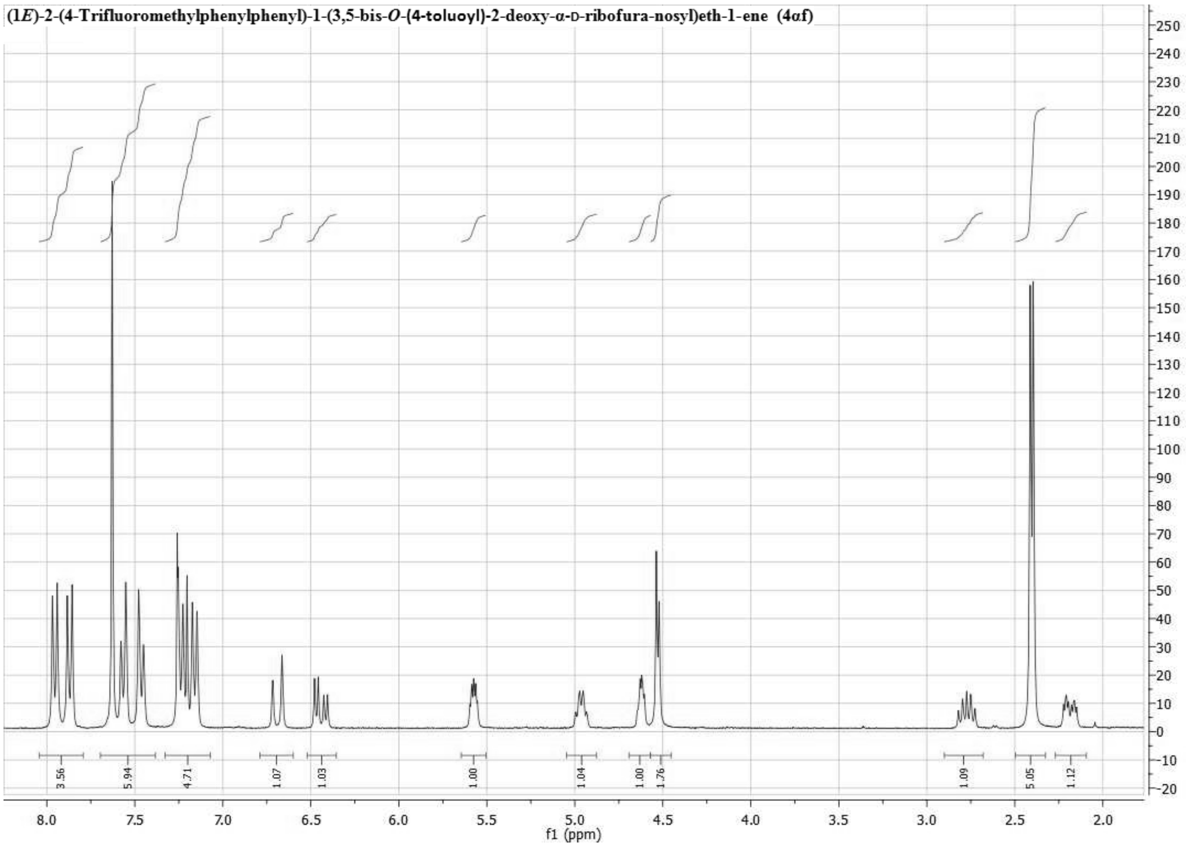

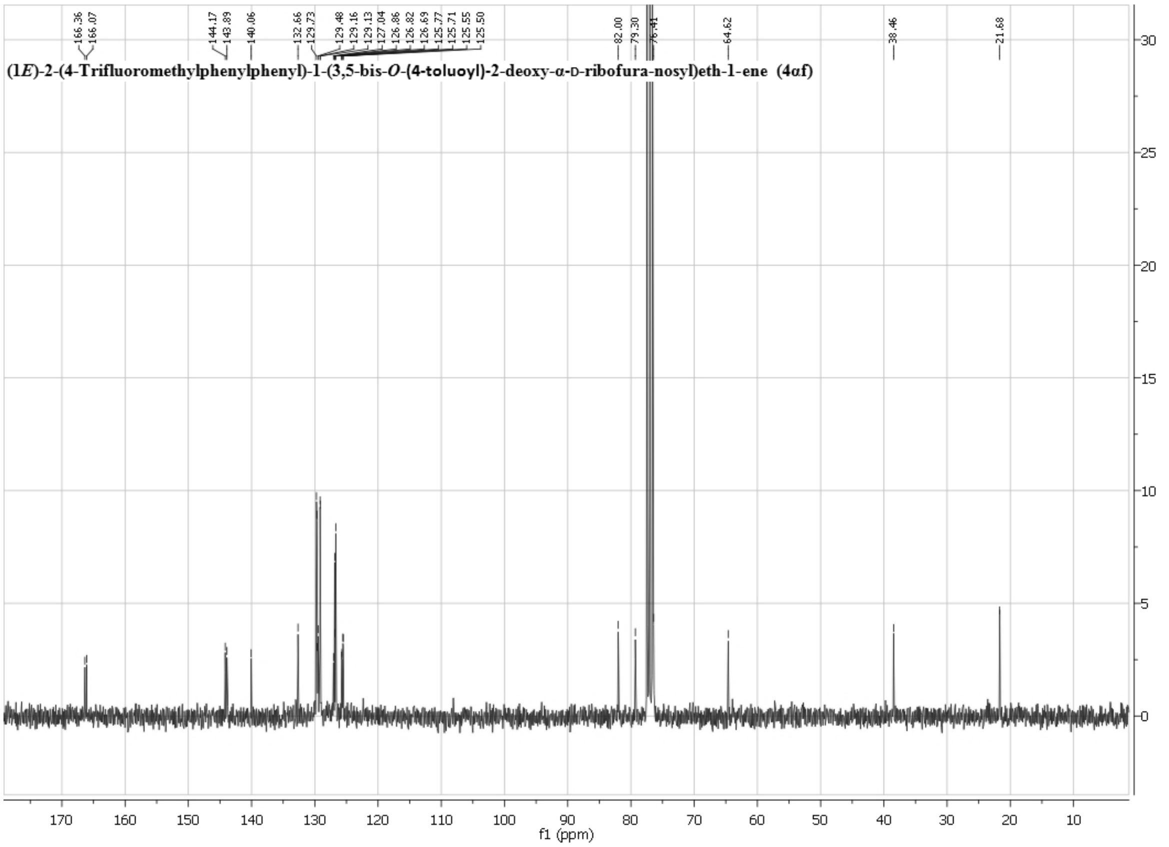

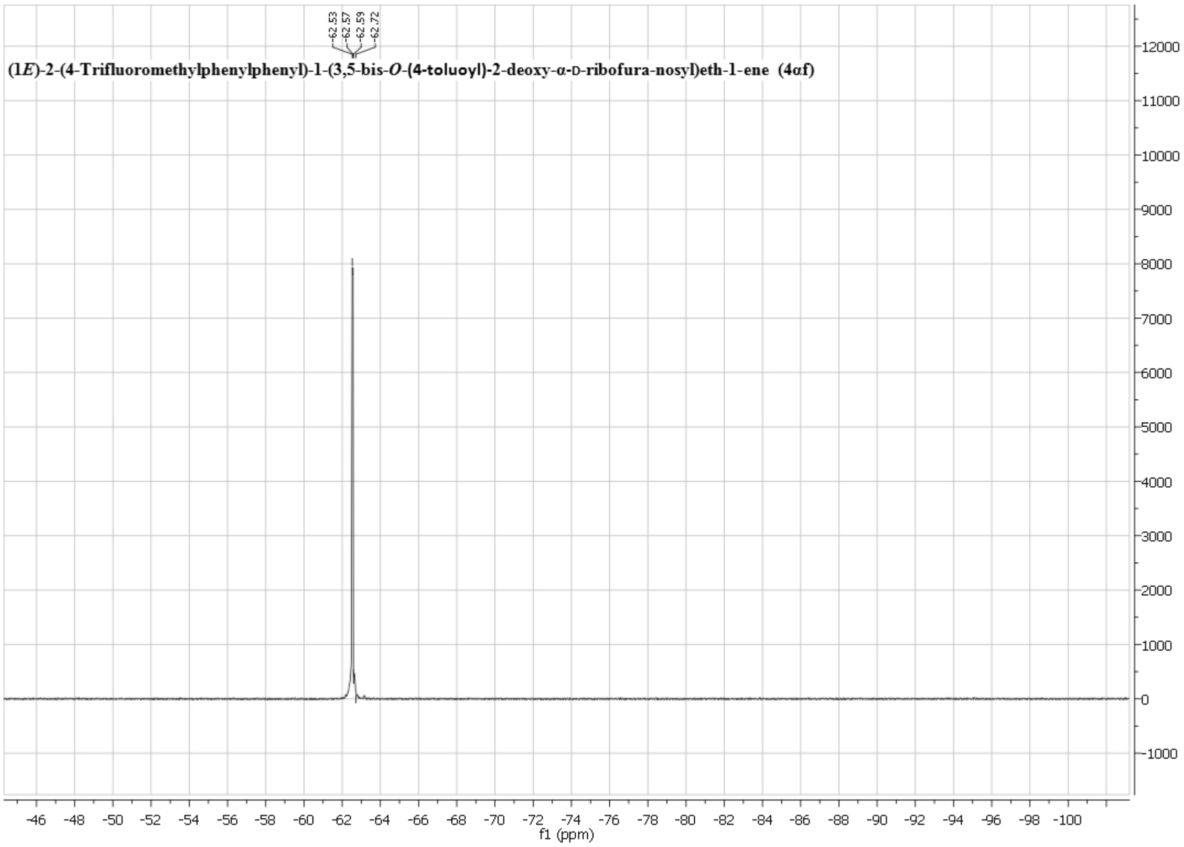
-62.53
-62.57
-62.59
-62.72
(1E)-2-(4-Trifluoromethylphenylphenyl)-1-(3,5-bis-O-(4-toluoyl)-2-deoxy-α-D-ribofura-nosyl)eth-1-ene (4αf)
12000
11000
10000
9000
8000
7000
6000
5000
4000
3000
2000
1000
0
-1000
-46 -48 -50 -52 -54 -56 -58 -60 -62 -64 -66 -68 -70 -72 -74 -76 -78 -80 -82 -84 -86 -88 -90 -92 -94 -96 -98 -100
f1 (ppm)

(1*E*)-2-(2-(4,4,5,5-Tetramethyl-1,3,2-dioxaborolan-2-yl)-1-(3,5-bis-*O*-(4-toluoyl)-2-deoxy-α-D-ribofuranosyl)ethene (α-4g).

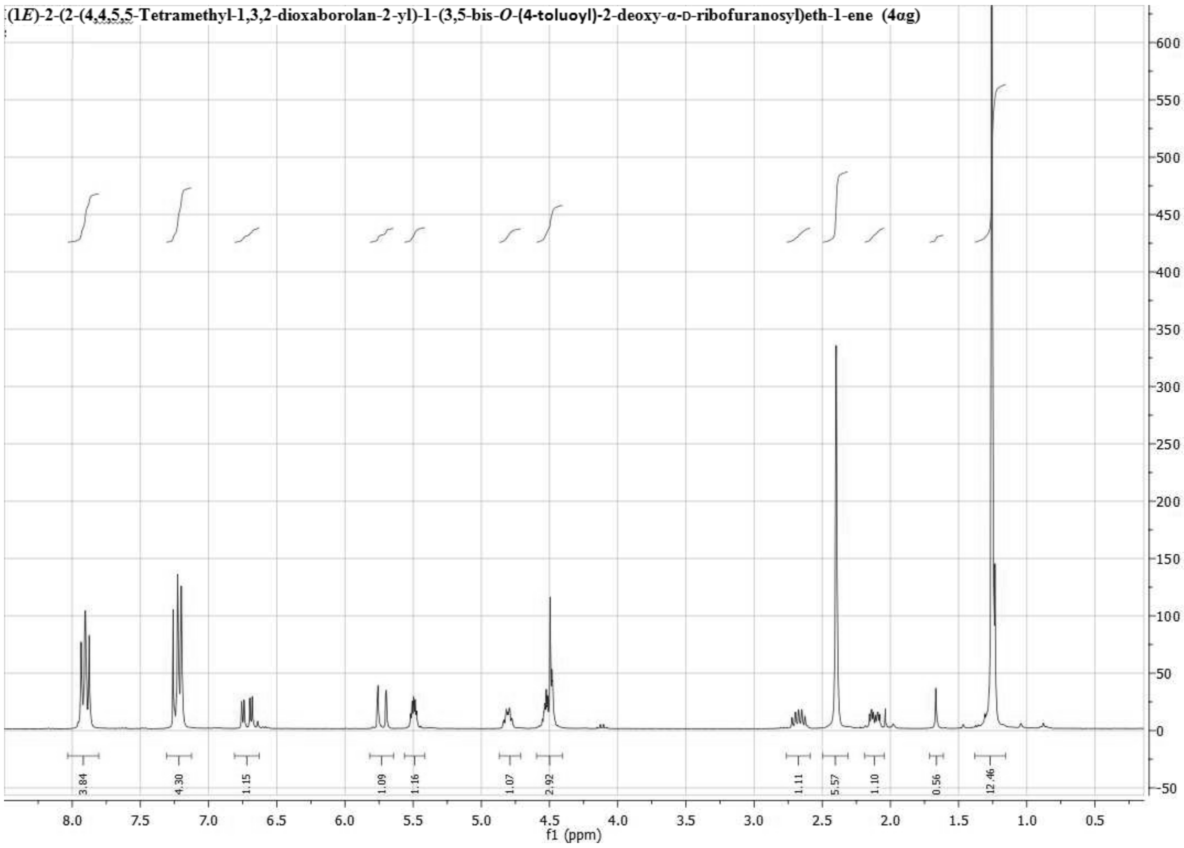

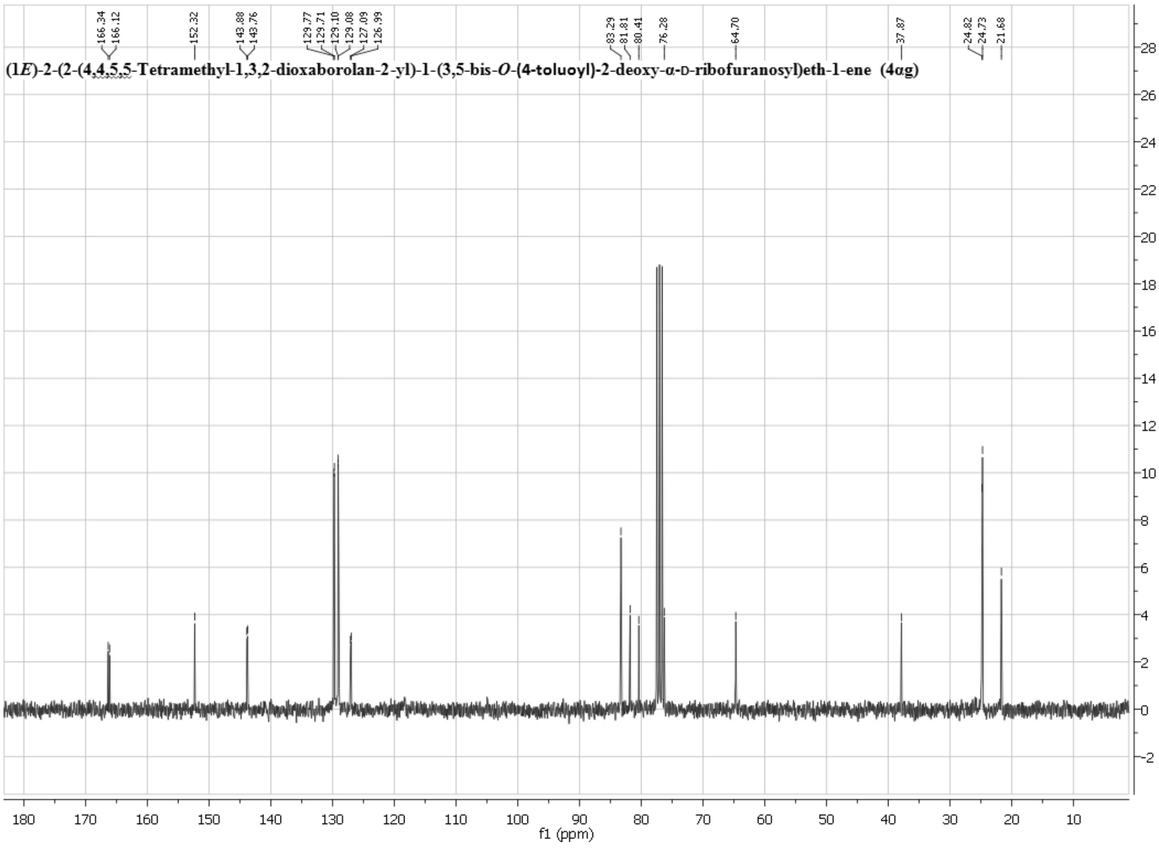

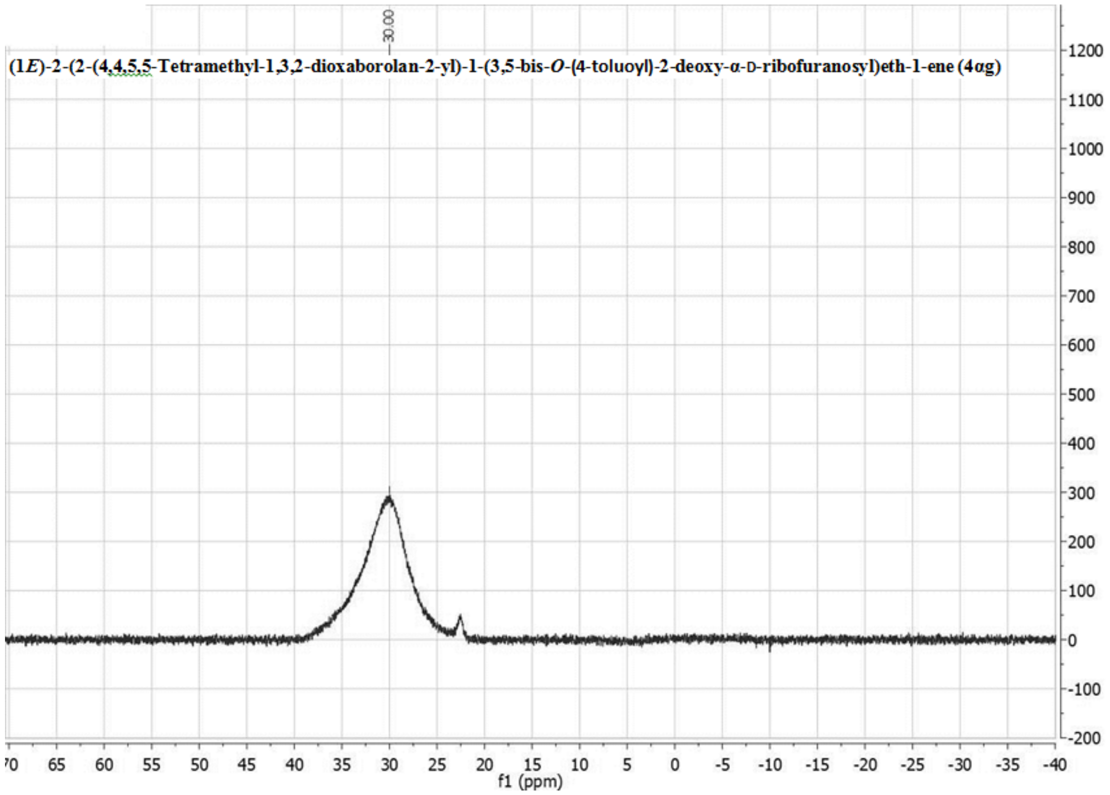

30.00
(1E)-2-(2-(4,4,5,5-Tetramethyl-1,3,2-dioxaborolan-2-yl)-1-(3,5-bis-O-(4-toluoyl)-2-deoxy-α-D-ribofuranosyl)eth-1-ene (4αg)
f1 (ppm)

(1*E*)-[3,5-Bis-*O*-(4-toluoyl)-2-deoxy-β-D-ribofuranosyl]-{8,8'-μ-(propen-3-yl-disulfido)-[3,3'-como-cobalt(III)-bis(1,2-dicarbaundecaborate)]} (β-4a).

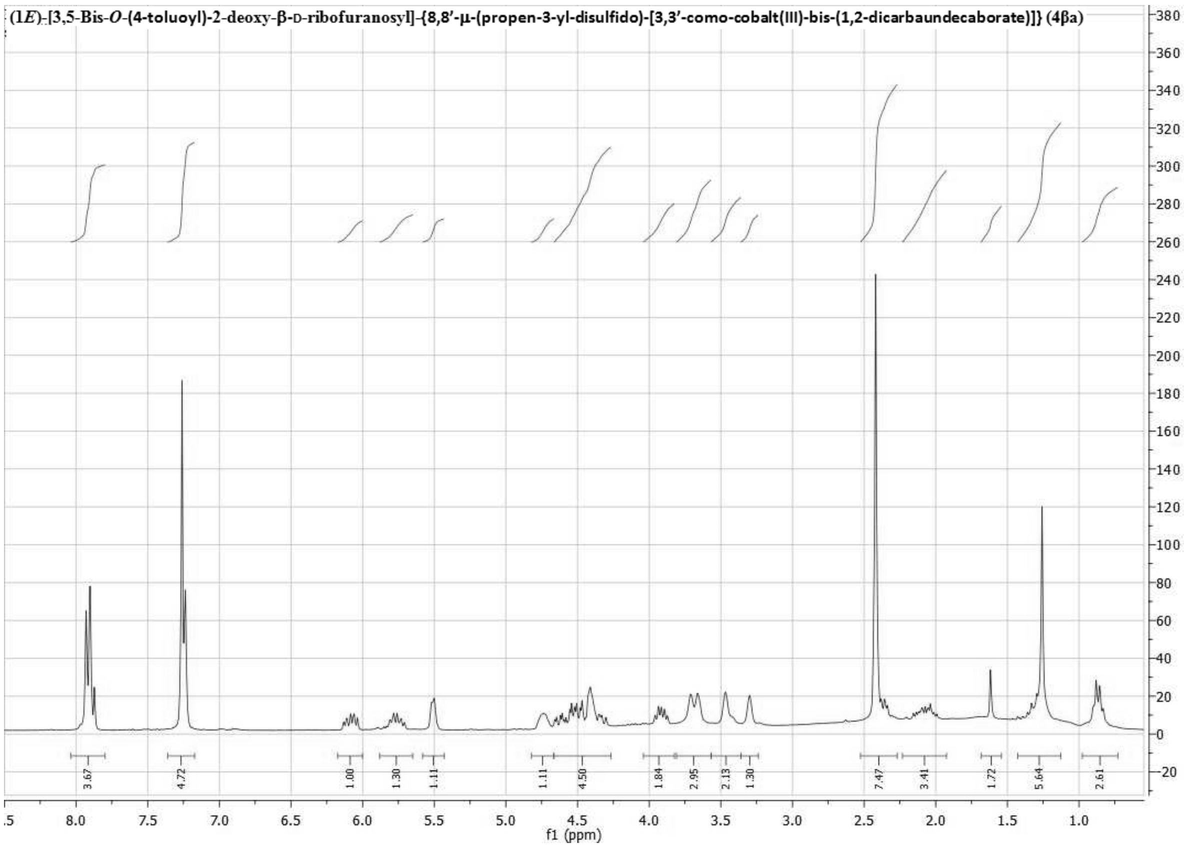

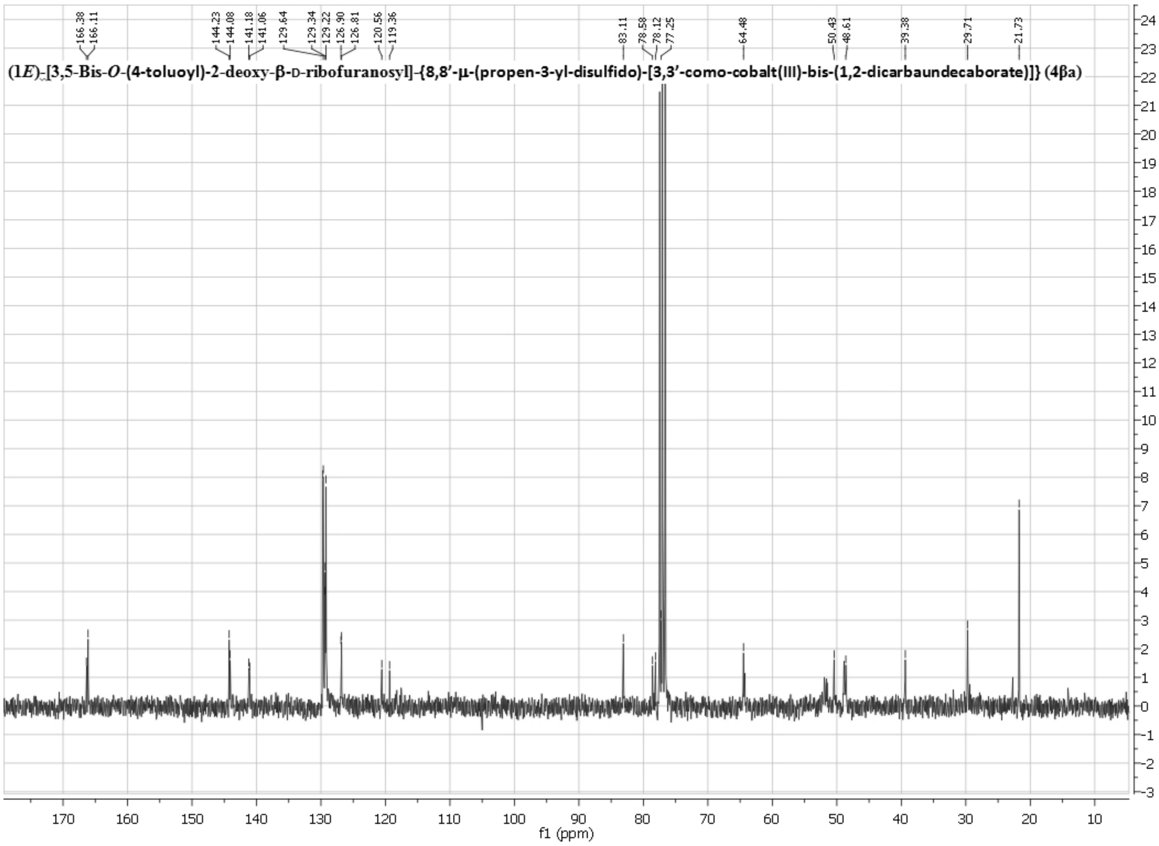

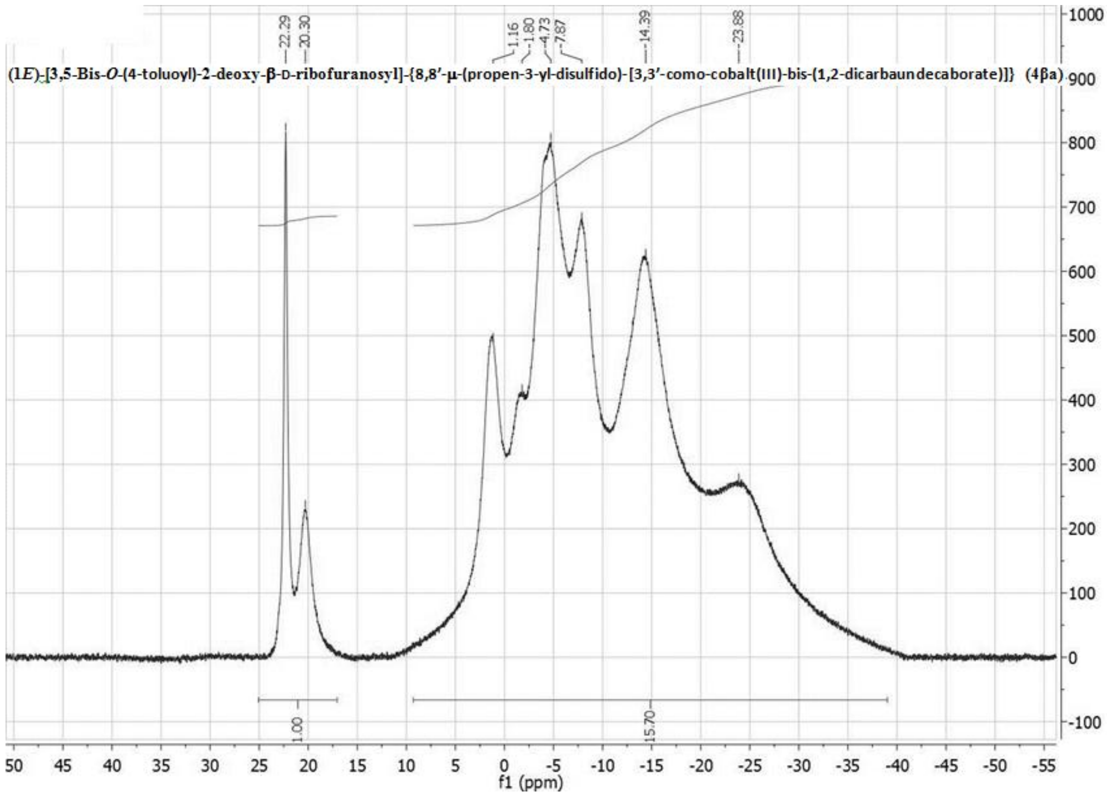

(1*E*)-[3,5-Bis-*O*-(4-toluoyl)-2-deoxy-β-D-ribofuranosyl]-{8,8′-μ-(propen-3-yl-disulfido)-[3,3′-como-cobalt(III)-bis-(1,2-dicarbaundecaborate)]} (4βa)
22.29
20.30
1.16
-1.80
-4.73
-7.87
-14.39
-23.88
1.00
15.70
f1 (ppm)

(1*E*)-1-(3,5-Bis-*O*-(4-toluoyl)-2-deoxy-β-D-ribofuranosyl)hept-1-ene (β-4b).

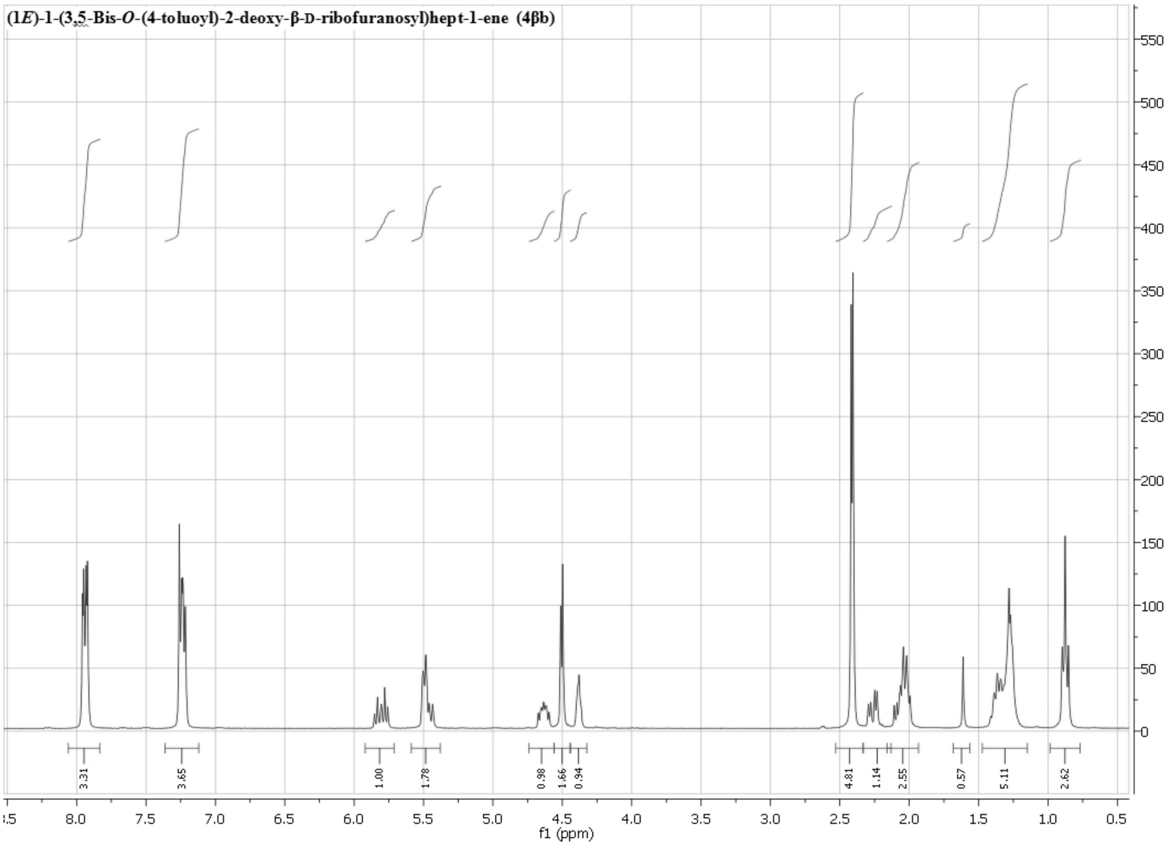

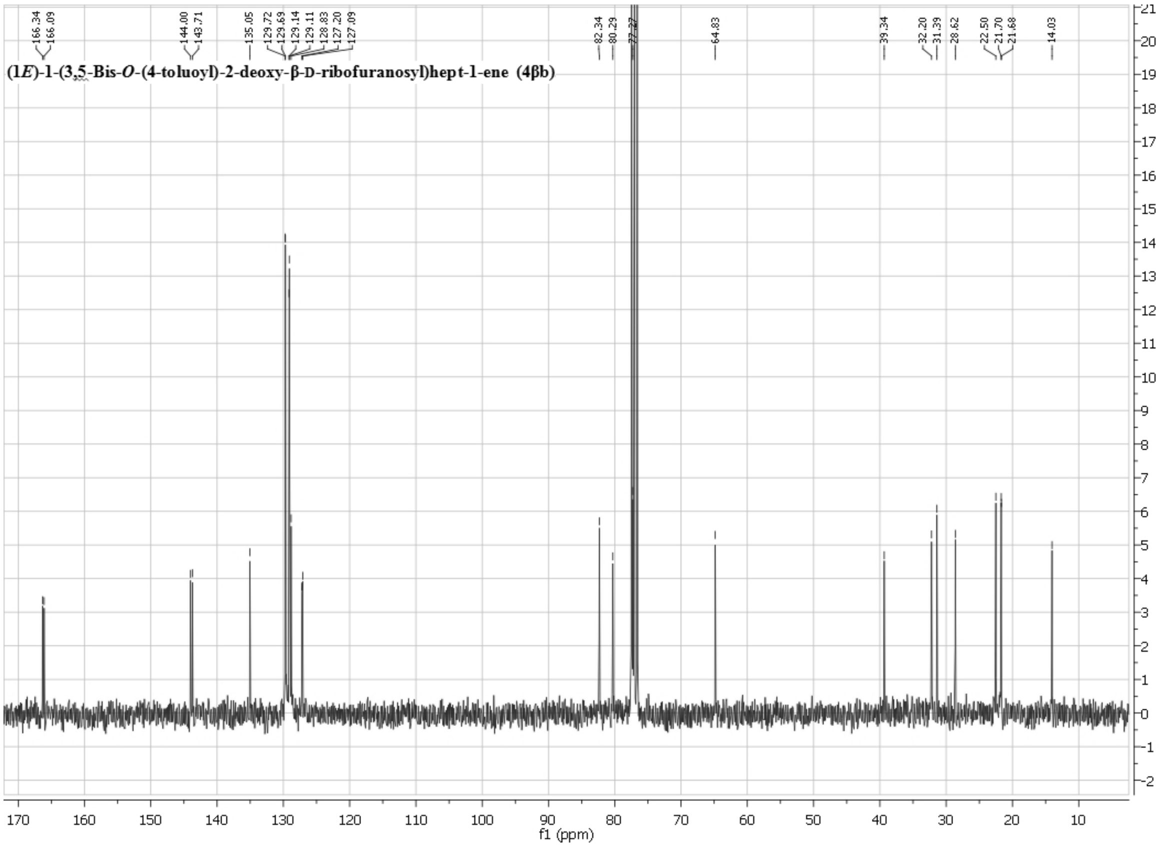

(1*E*)-1-(3,5-Bis-*O*-(4-toluoyl)-2-deoxy-β-D-ribofuranosyl)-4,4,5,5,6,6,7,7,8,8,9,9,9-trideca-fluoronon-1-ene (β-4c).

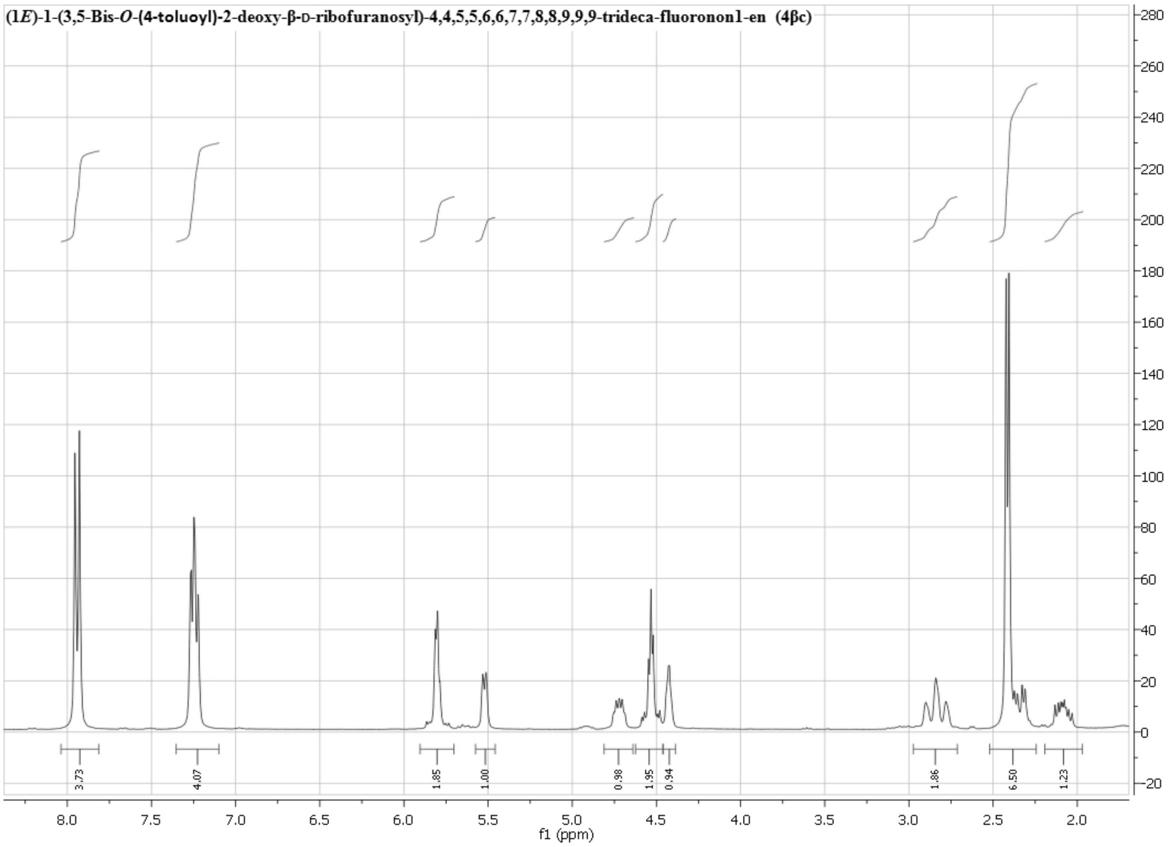

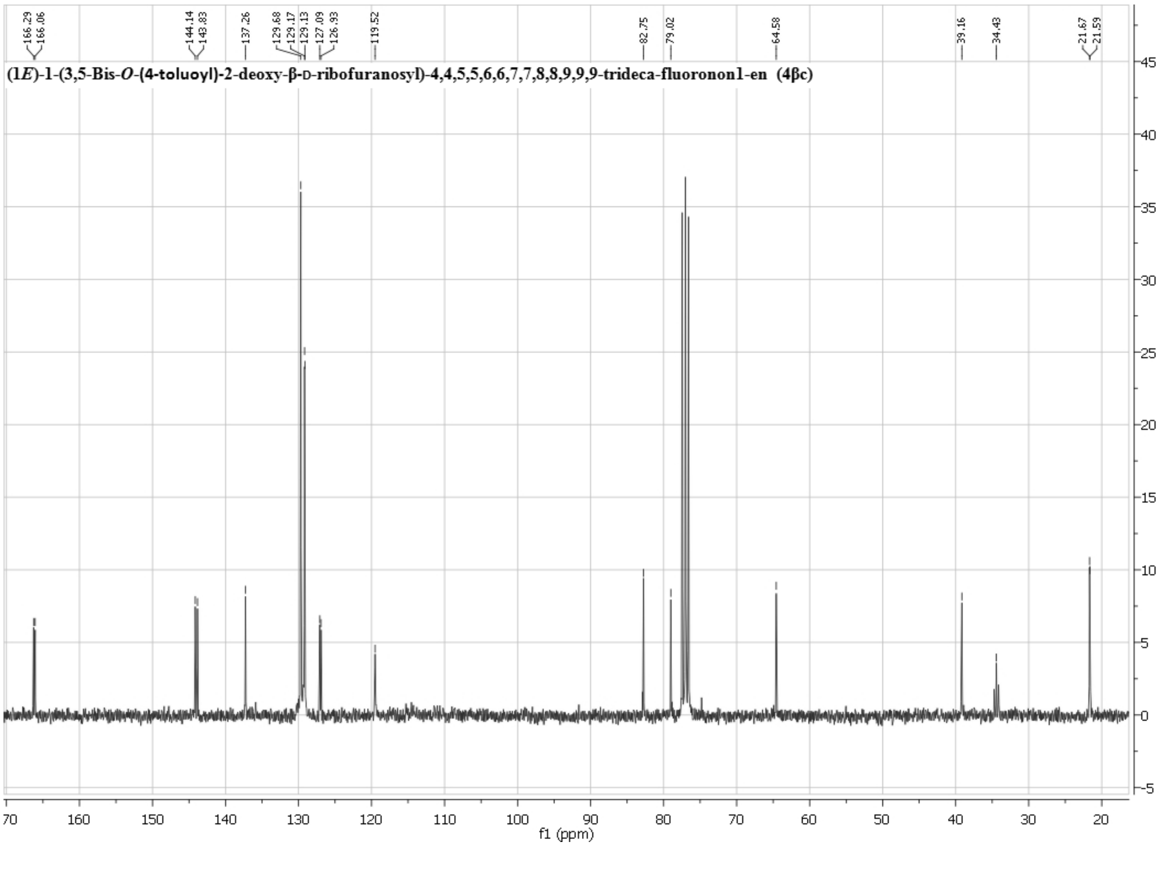

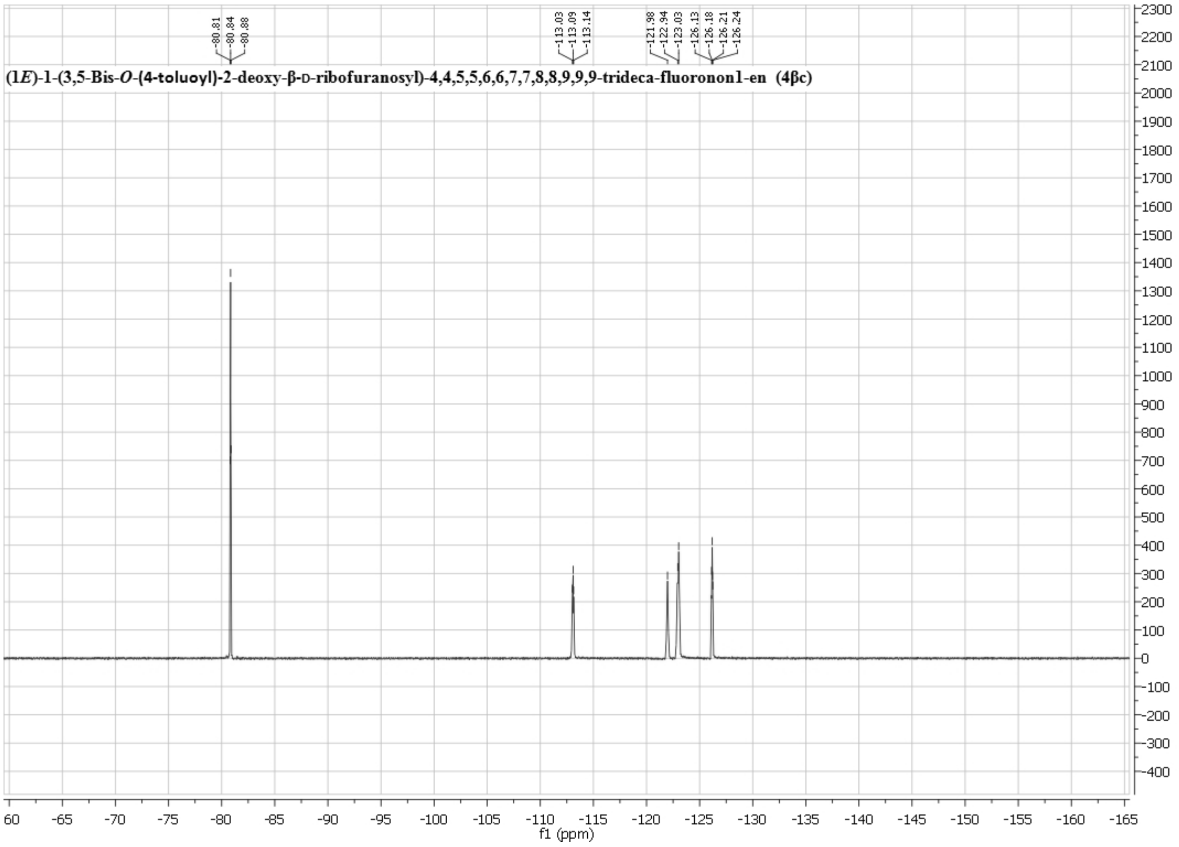
-80.81
-80.84
-80.88
-113.03
-113.09
-113.14
-121.98
-122.94
-123.03
-126.13
-126.18
-126.21
-126.24
(1*E*)-1-(3,5-Bis-*O*-(4-toluoyl)-2-deoxy-β-D-ribofuranosyl)-4,4,5,5,6,6,7,7,8,8,9,9,9-trideca-fluoronon1-en (4βc)
60 -65 -70 -75 -80 -85 -90 -95 -100 -105 -110 -115 -120 -125 -130 -135 -140 -145 -150 -155 -160 -165
f1 (ppm)
2300 2200 2100 2000 1900 1800 1700 1600 1500 1400 1300 1200 1100 1000 900 800 700 600 500 400 300 200 100 0 -100 -200 -300 -400

(1*E*)-2-Phenyl-1-(3,5-bis-*O*-(4-toluoyl)-2-deoxy-β-D-ribofuranosyl)ethene (β-4d).

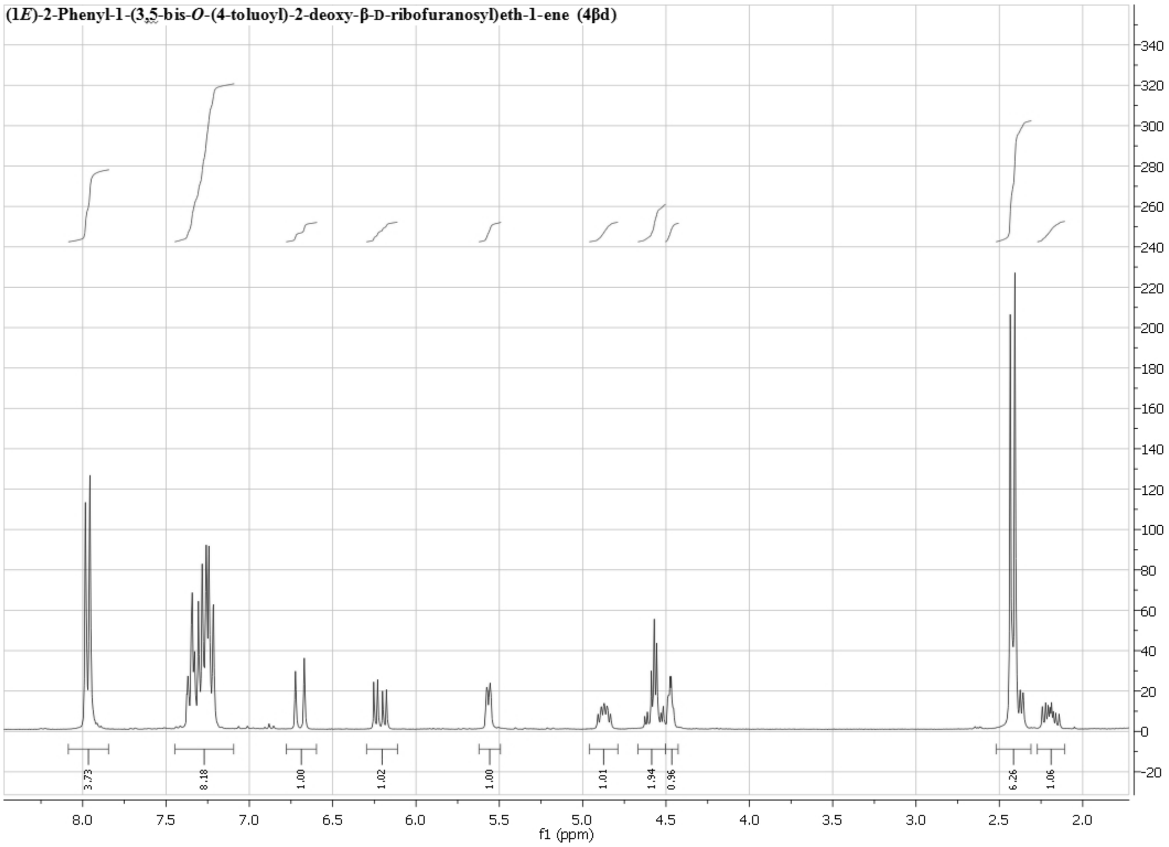

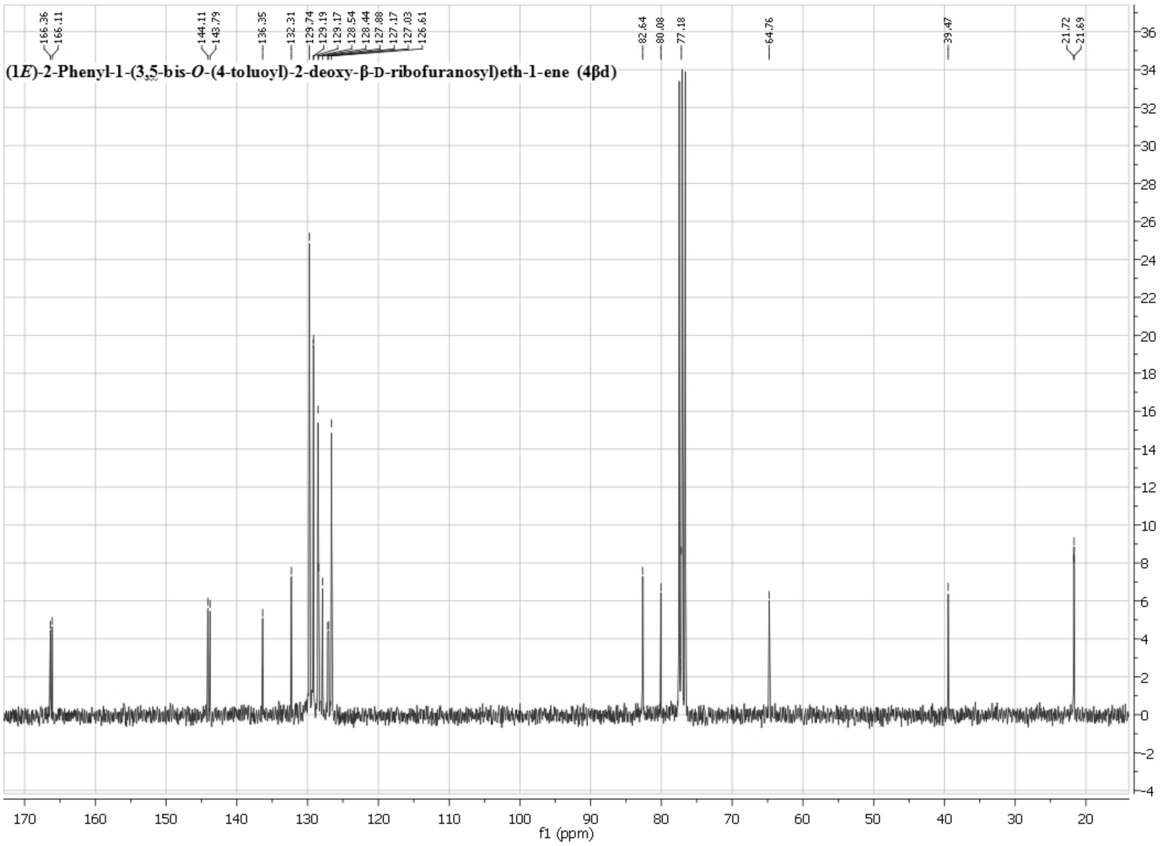

(1*E*)-2-(4-Fluorophenyl)-1-(3,5-bis-*O*-(4-toluoyl)-2-deoxy-β-D-ribofuranosyl)ethene (β-4e).

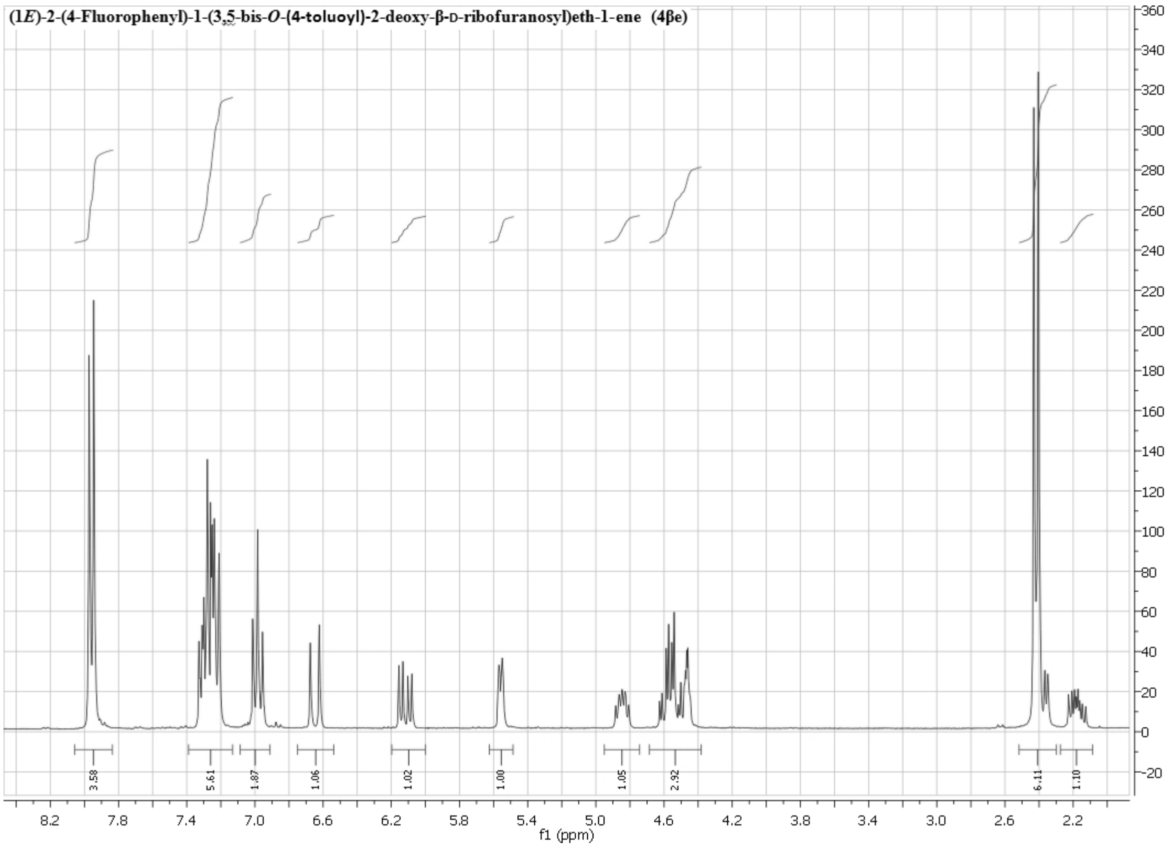

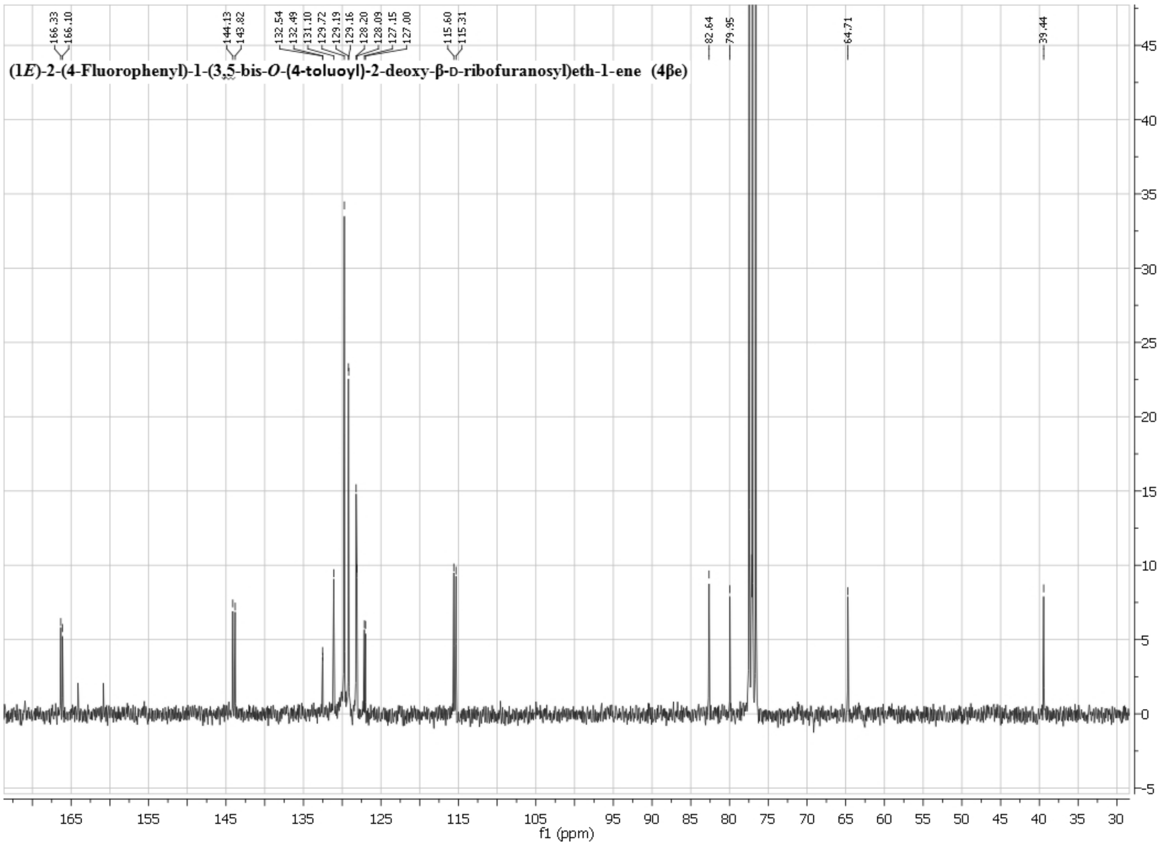

(1*E*)-2-(4-Fluorophenyl)-1-(3,5-bis-*O*-(4-toluoyl)-2-deoxy-β-D-ribofuranosyl)eth-1-ene (4βe)

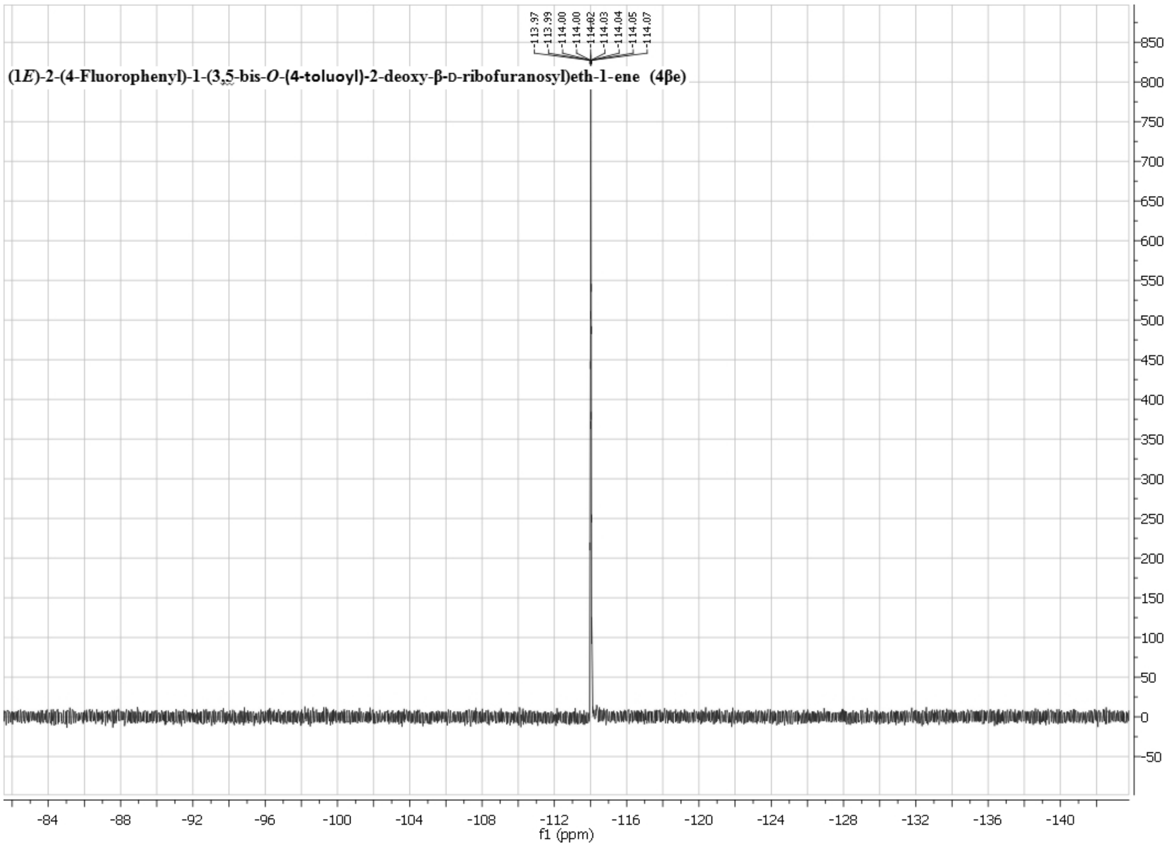

(1*E*)-2-(4-Trifluoromethylphenyl)-1-(3,5-bis-*O*-(4-toluoyl)-2-deoxy-β-D-ribofuranosyl)-ethene (β-4f).

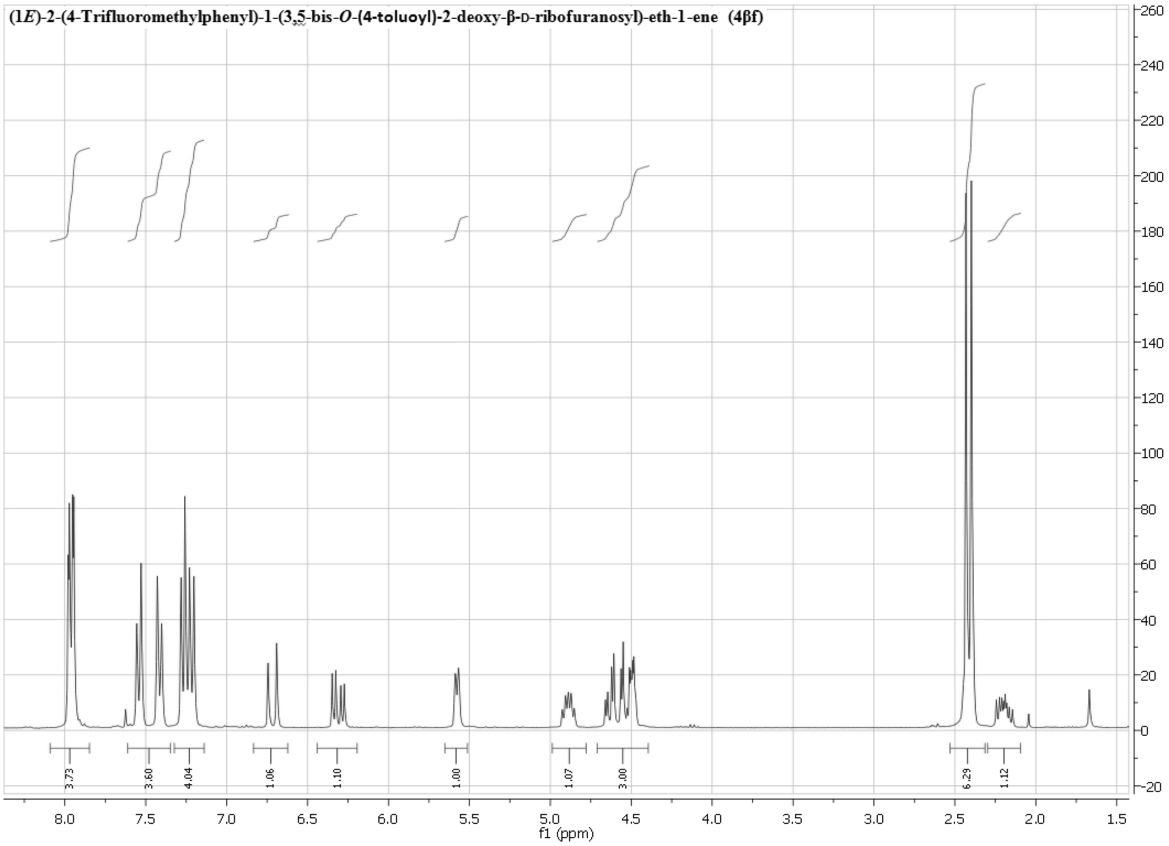

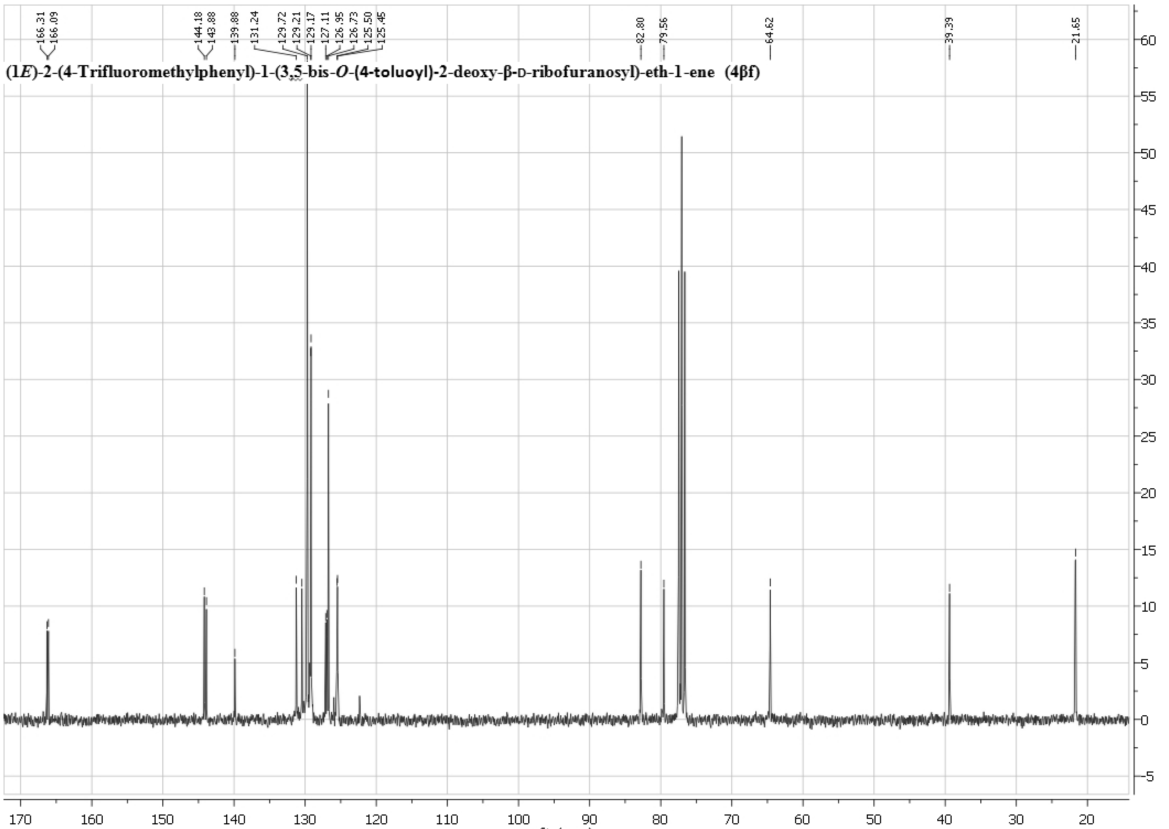

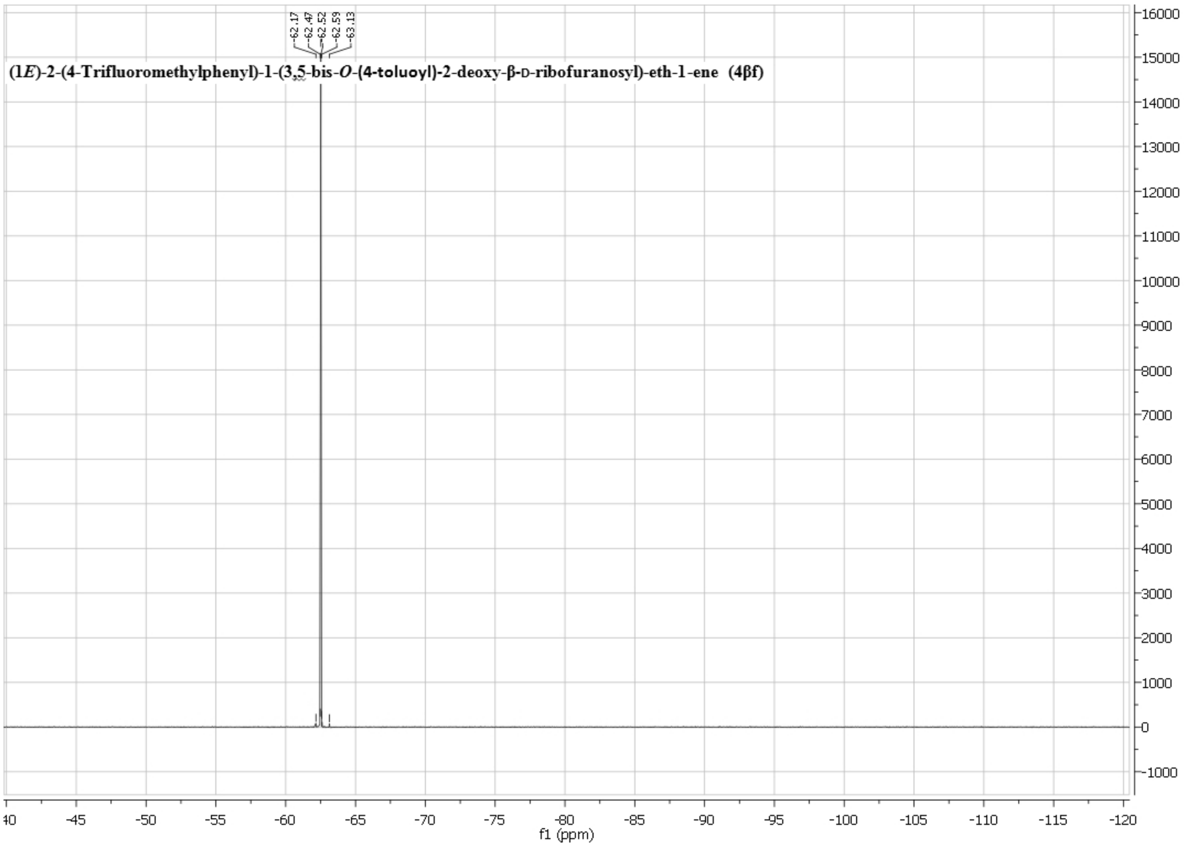

-62.17
-62.47
-62.52
-62.59
-63.13
(1*E*)-2-(4-Trifluoromethylphenyl)-1-(3,5-bis-*O*-(4-toluoyl)-2-deoxy-β-D-ribofuranosyl)-eth-1-ene (4βf)
f1 (ppm)

(1*E*)-2-(2-(4,4,5,5-Tetramethyl-1,3,2-dioxaborolan-2-yl)-1-(3,5-bis-*O*-(4-toluoyl)-2-deoxy-β-D-ribofuranosyl)ethene (β-4g).

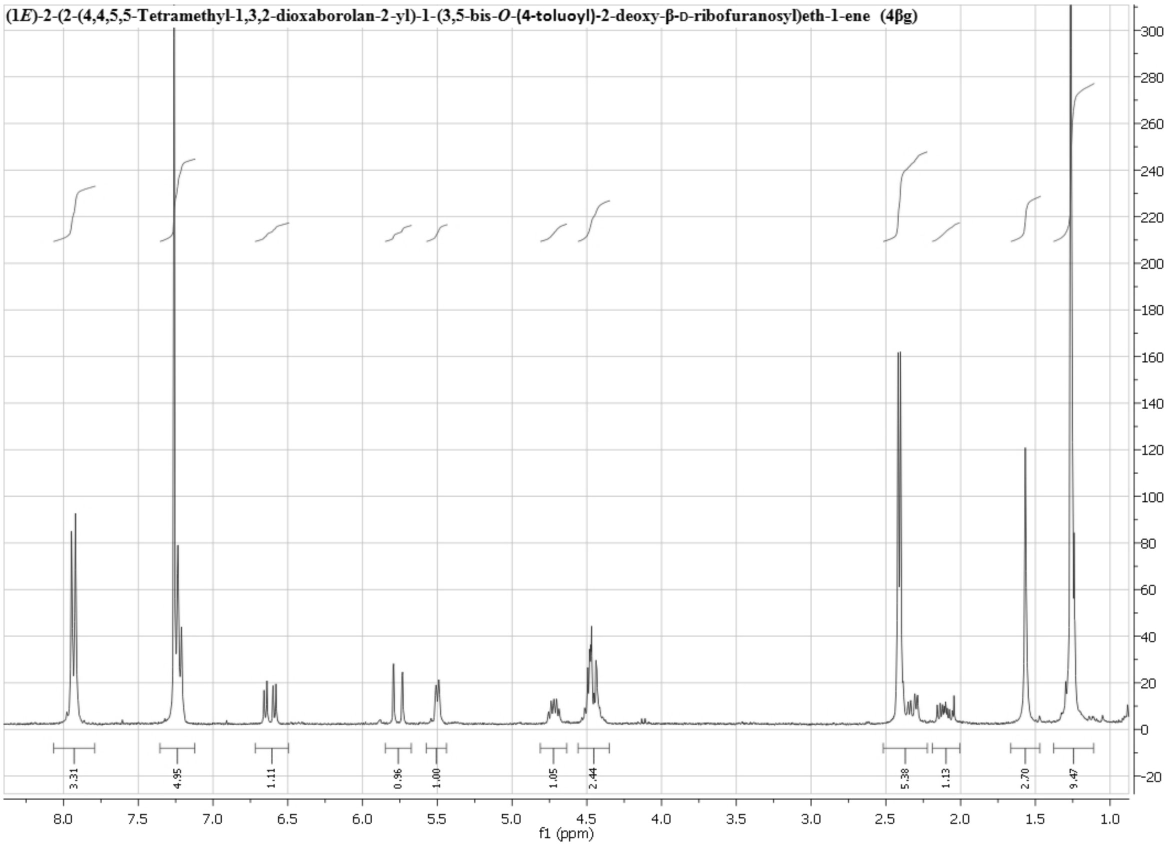

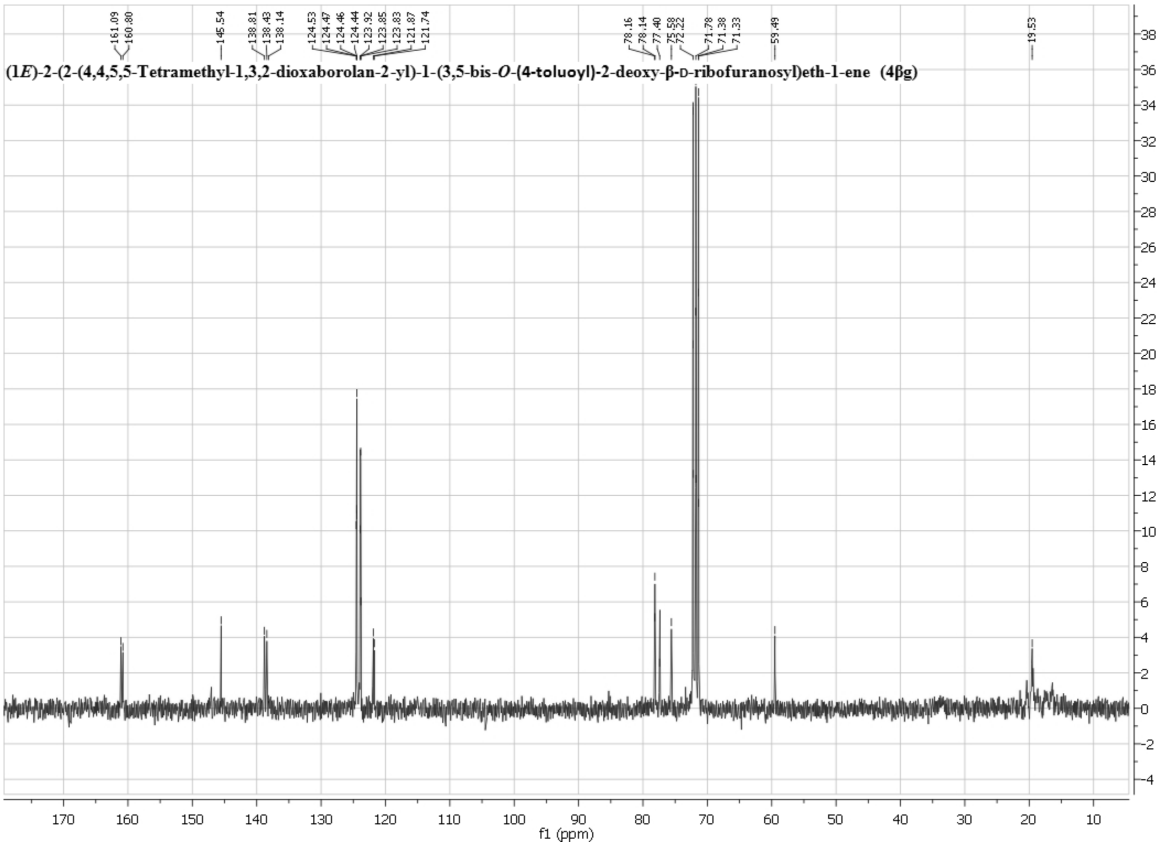

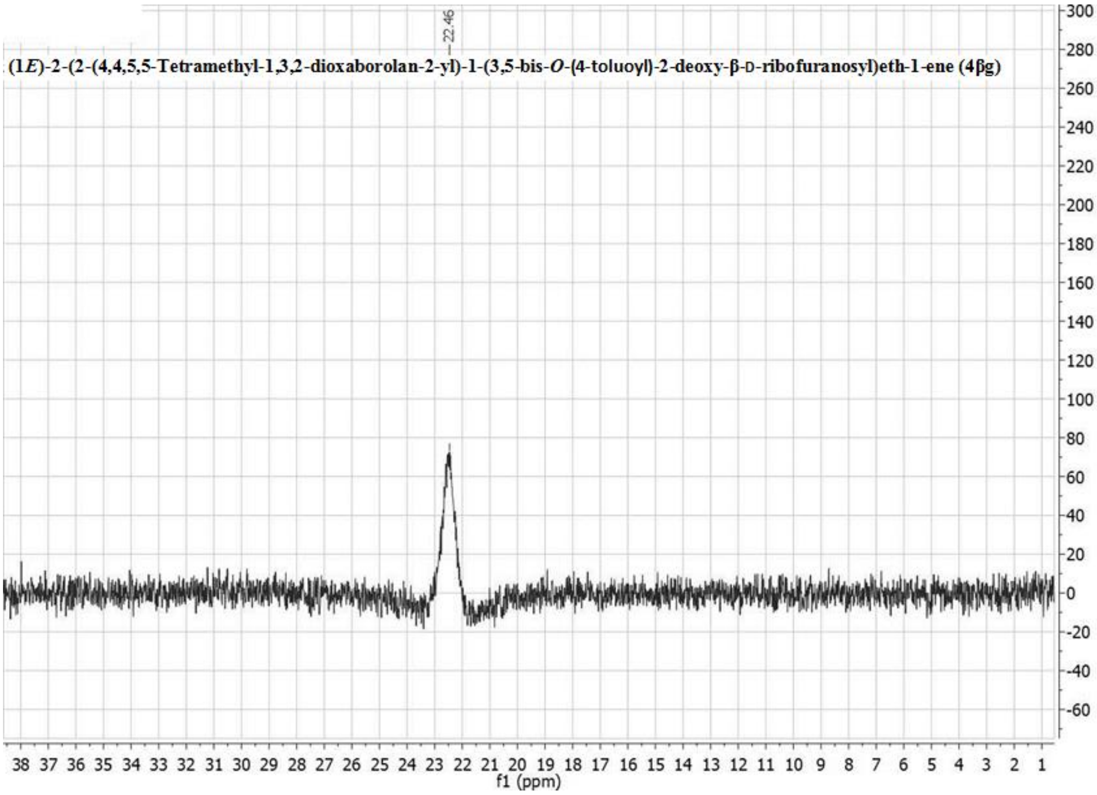

(1*E*)-2-(2-(4,4,5,5-Tetramethyl-1,3,2-dioxaborolan-2-yl)-1-(3,5-bis-*O*-(4-toluoyl)-2-deoxy-β-D-ribofuranosyl)eth-1-ene (4βg)

1-(3,5-Bis-*O*-(4-toluoyl)-2-deoxy-β-D-ribofuranosyl)heptane (β-5b).

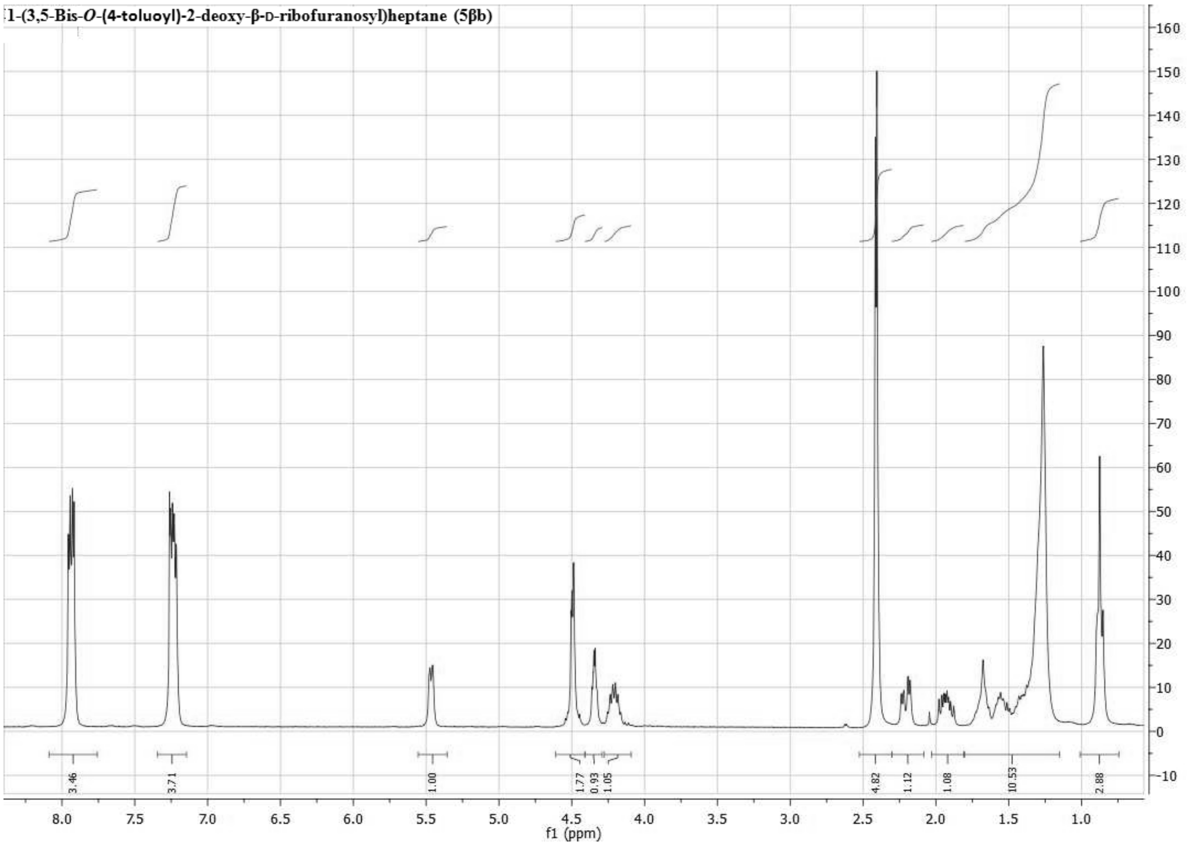

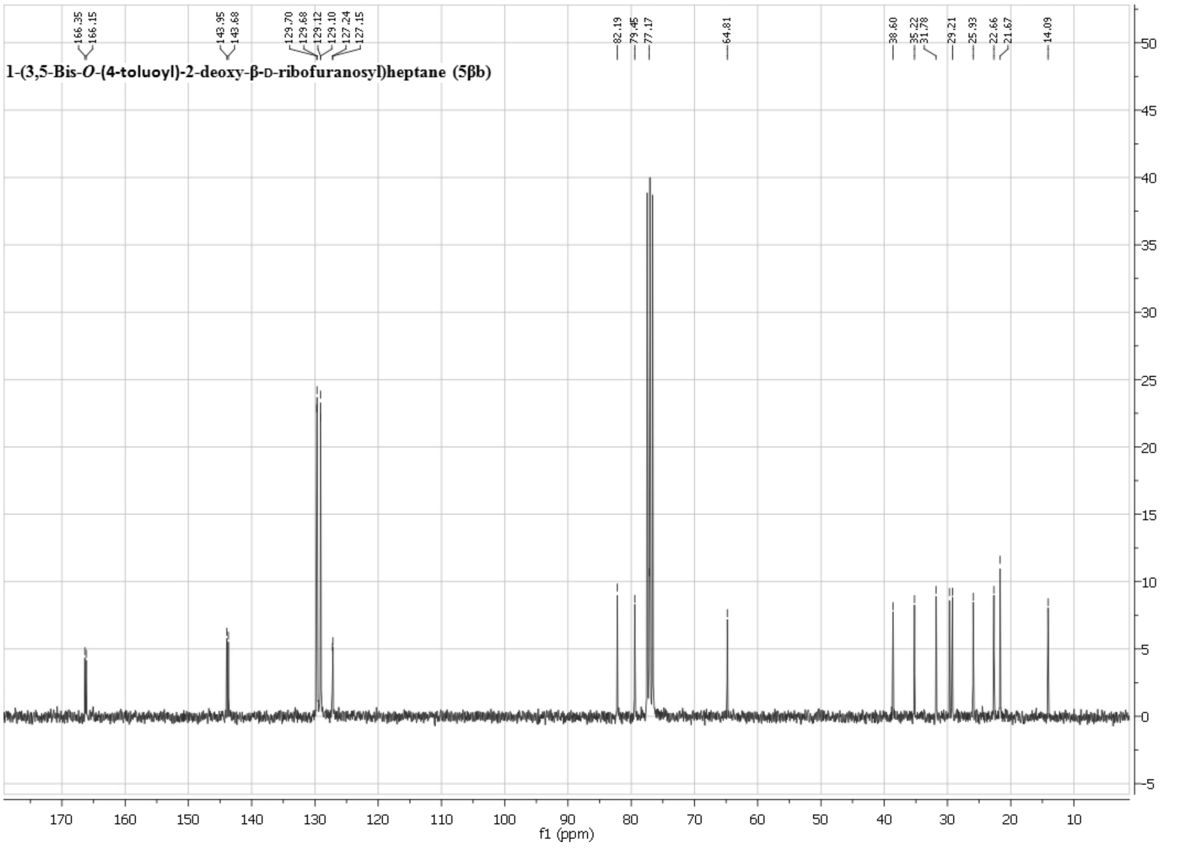

1-(3,5-Bis-*O*-(4-toluoyl)-2-deoxy-β-D-ribofuranosyl)-4,4,5,5,6,6,7,7,8,8,9,9,9-tridecafluororononane (β-5c).

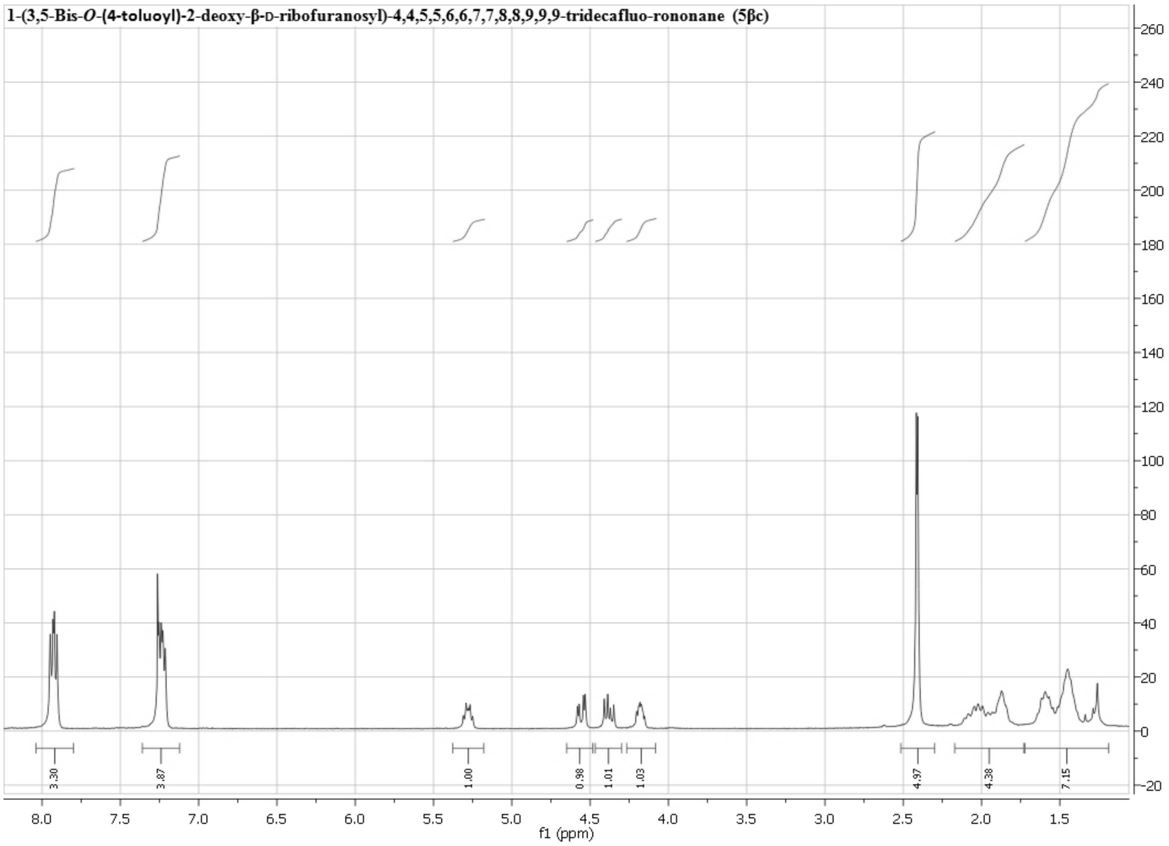

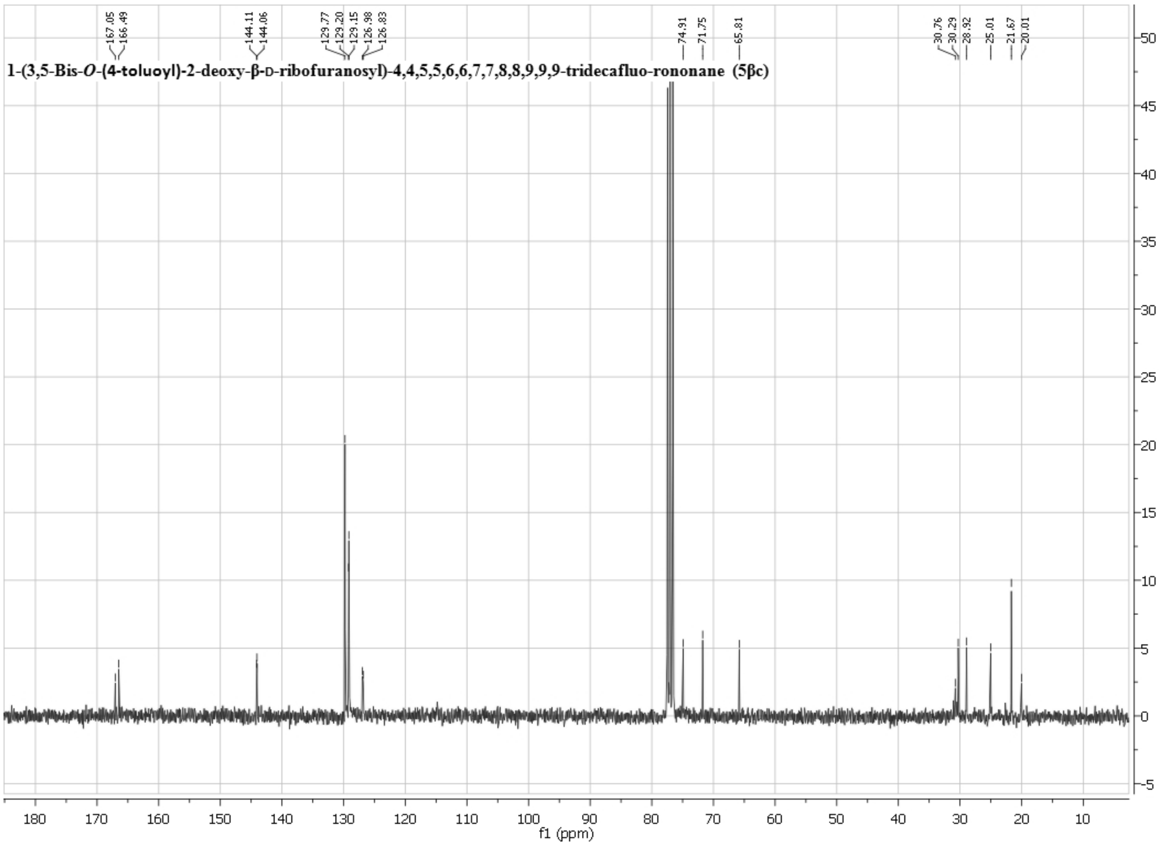

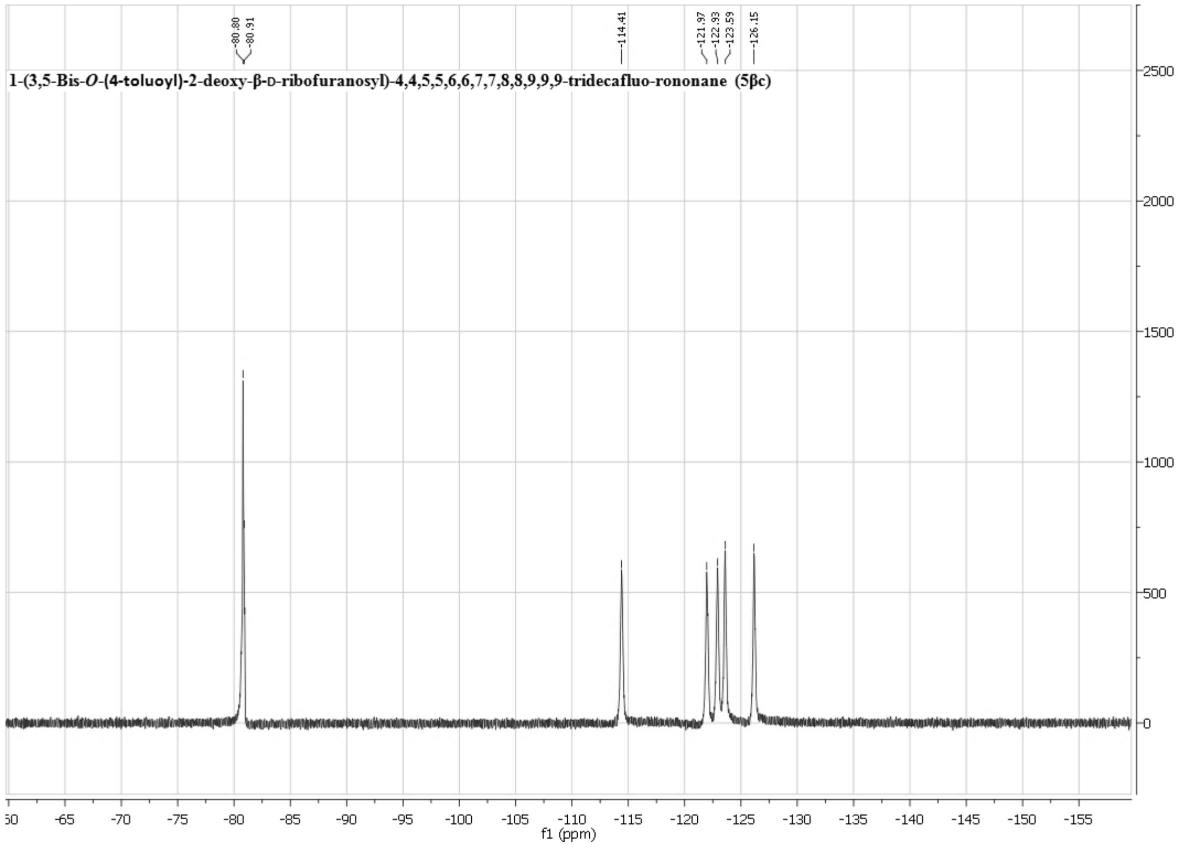
1-(3,5-Bis-O-(4-toluoyl)-2-deoxy-β-D-ribofuranosyl)-4,4,5,5,6,6,7,7,8,8,9,9,9-tridecafluo-rononane (5βc)
-80.80
-80.91
-114.41
-121.97
-122.93
-123.59
-126.15
2500
2000
1500
1000
500
0
-65
-70
-75
-80
-85
-90
-95
-100
-105
-110
-115
-120
-125
-130
-135
-140
-145
-150
-155
f1 (ppm)

2-(Phenyl)-1-(3,5-bis-*O*-(4-toluoyl)-2-deoxy-β-D-ribofuranosyl)ethane (β-5d).

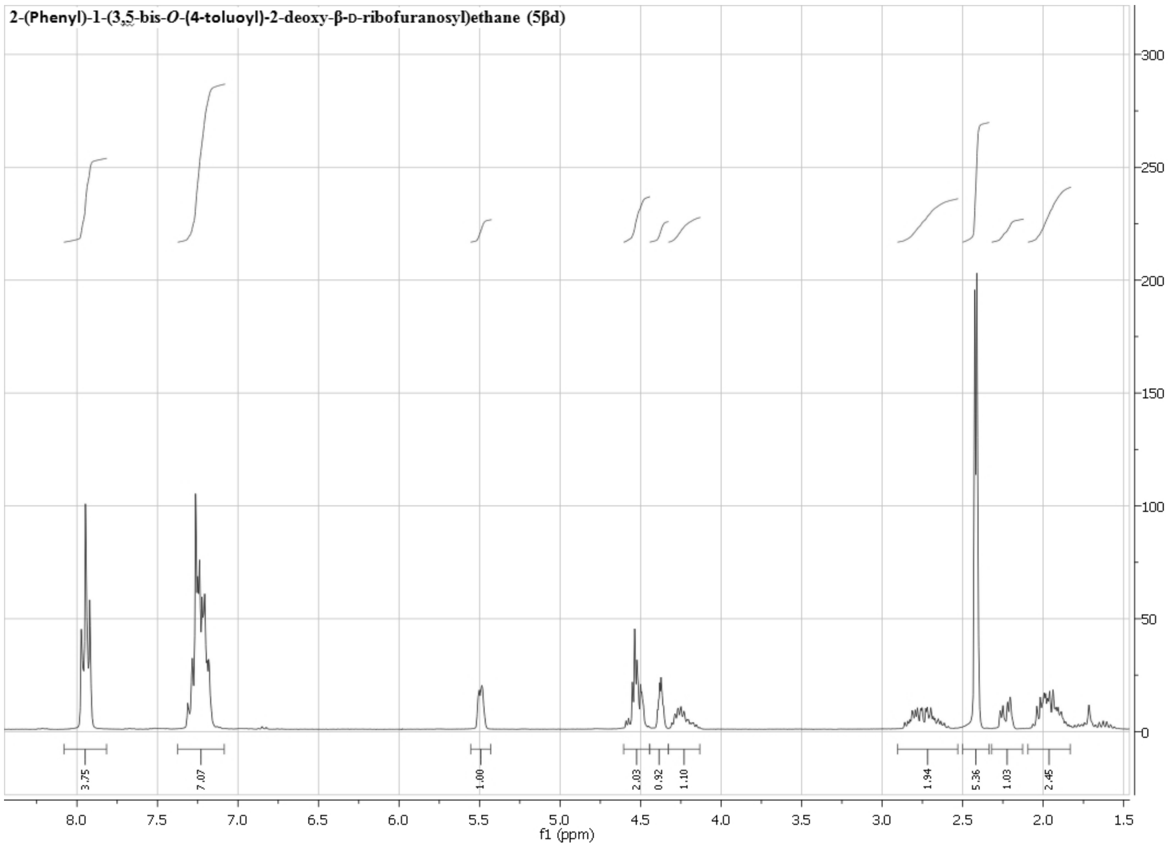

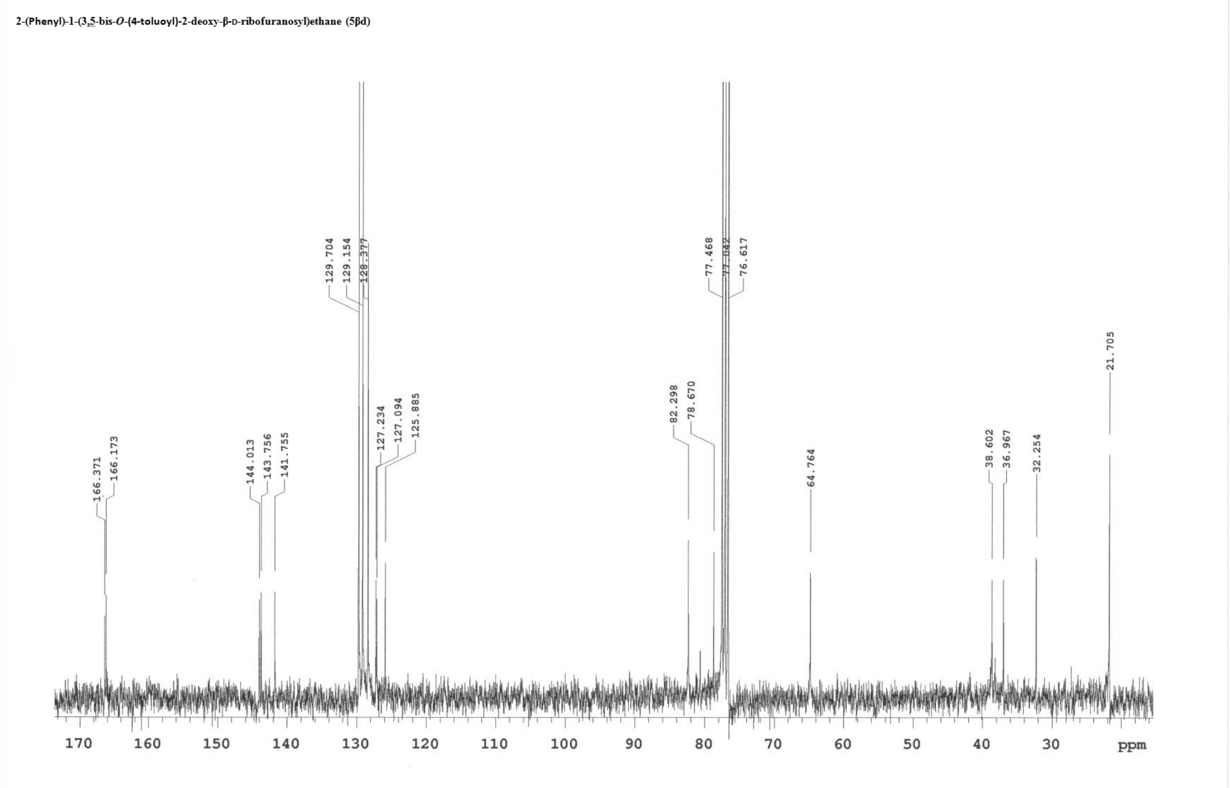

(1*E*)-2-(4-Fluorophenyl)-1-(2-deoxy-β-D-ribofuranosyl)ethene (β-6e).

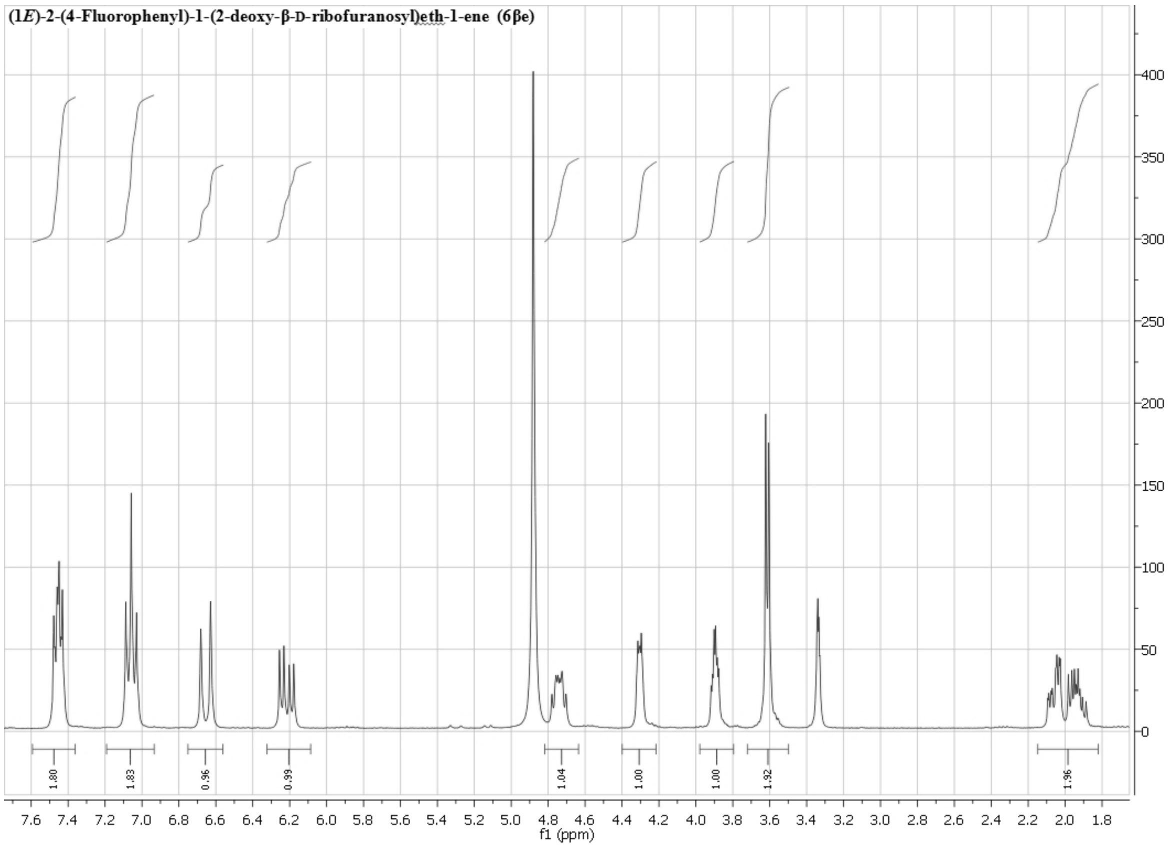

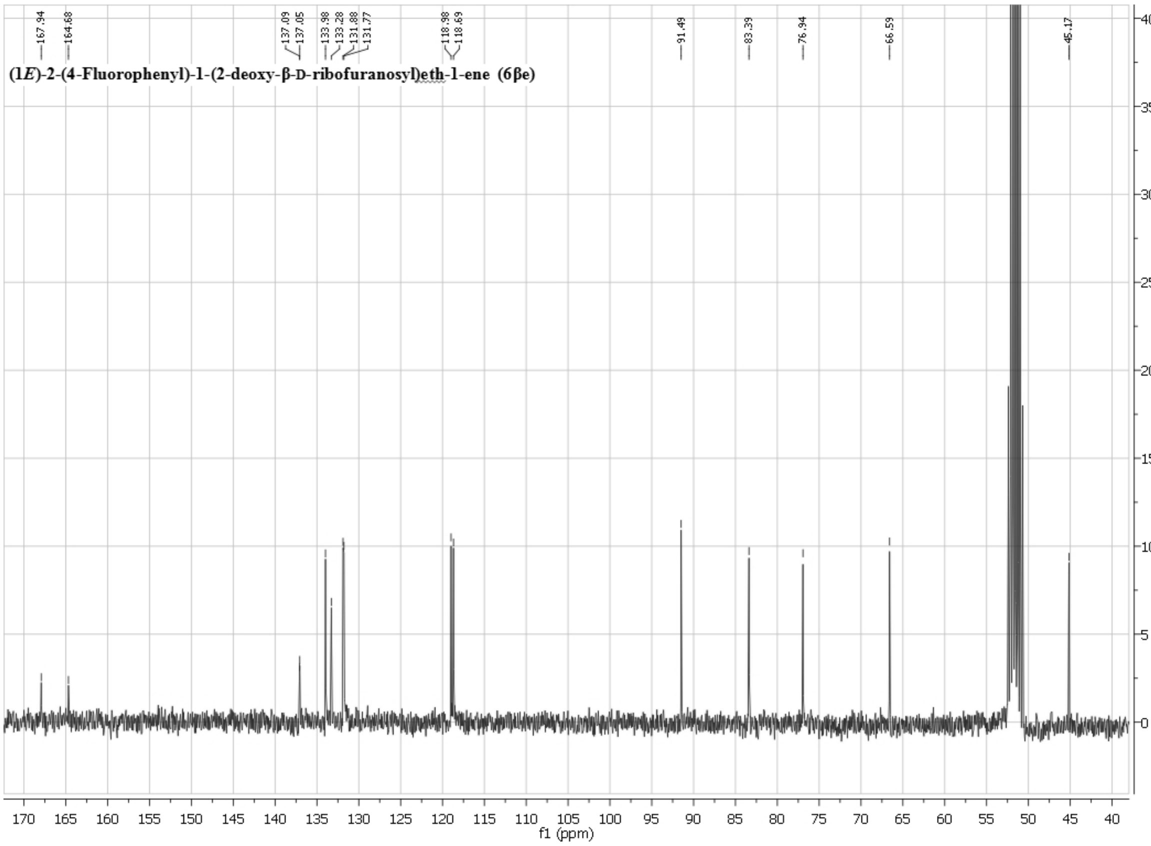

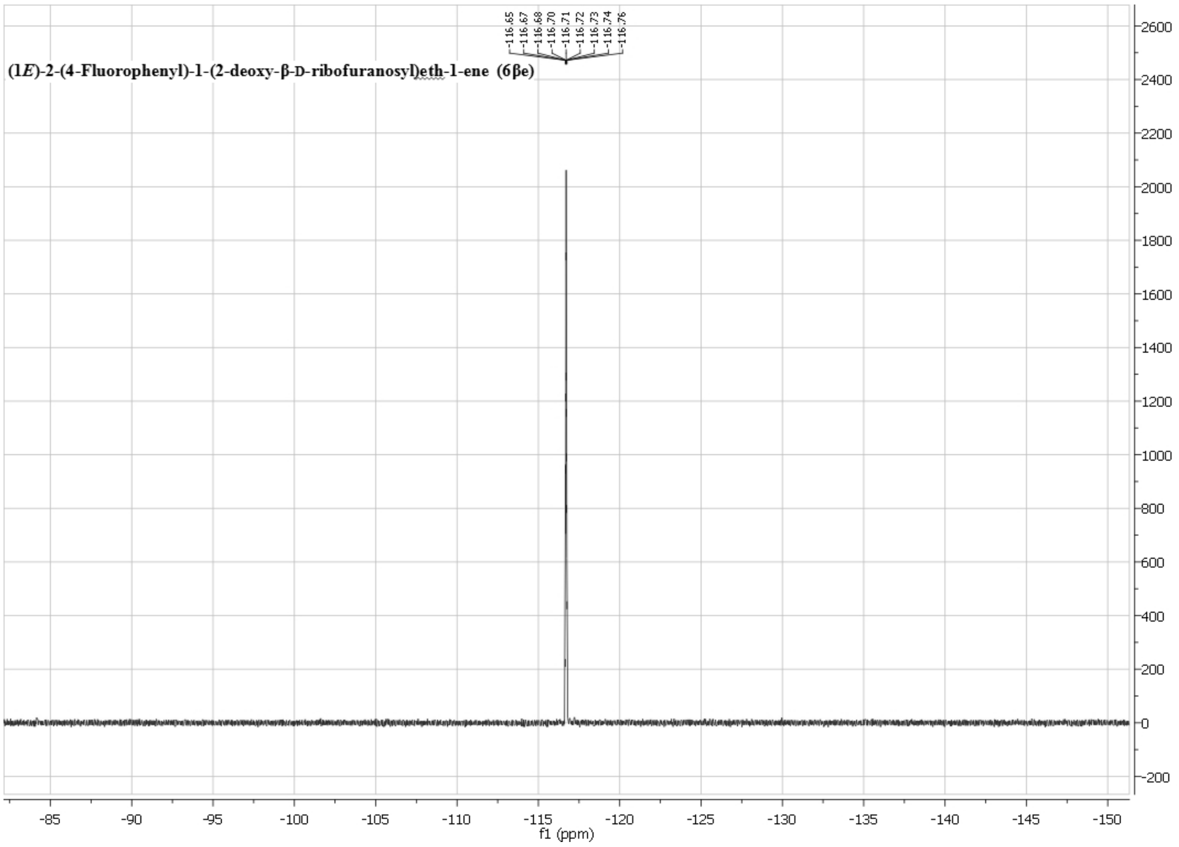
(1E)-2-(4-Fluorophenyl)-1-(2-deoxy-β-D-ribofuranosyl)eth-1-ene (6βe)
-116.65
-116.67
-116.68
-116.70
-116.71
-116.72
-116.73
-116.74
-116.76
f1 (ppm)

(1*E*)-1-(2-Deoxy-β-D-ribofuranosyl)heptane (β-7b).

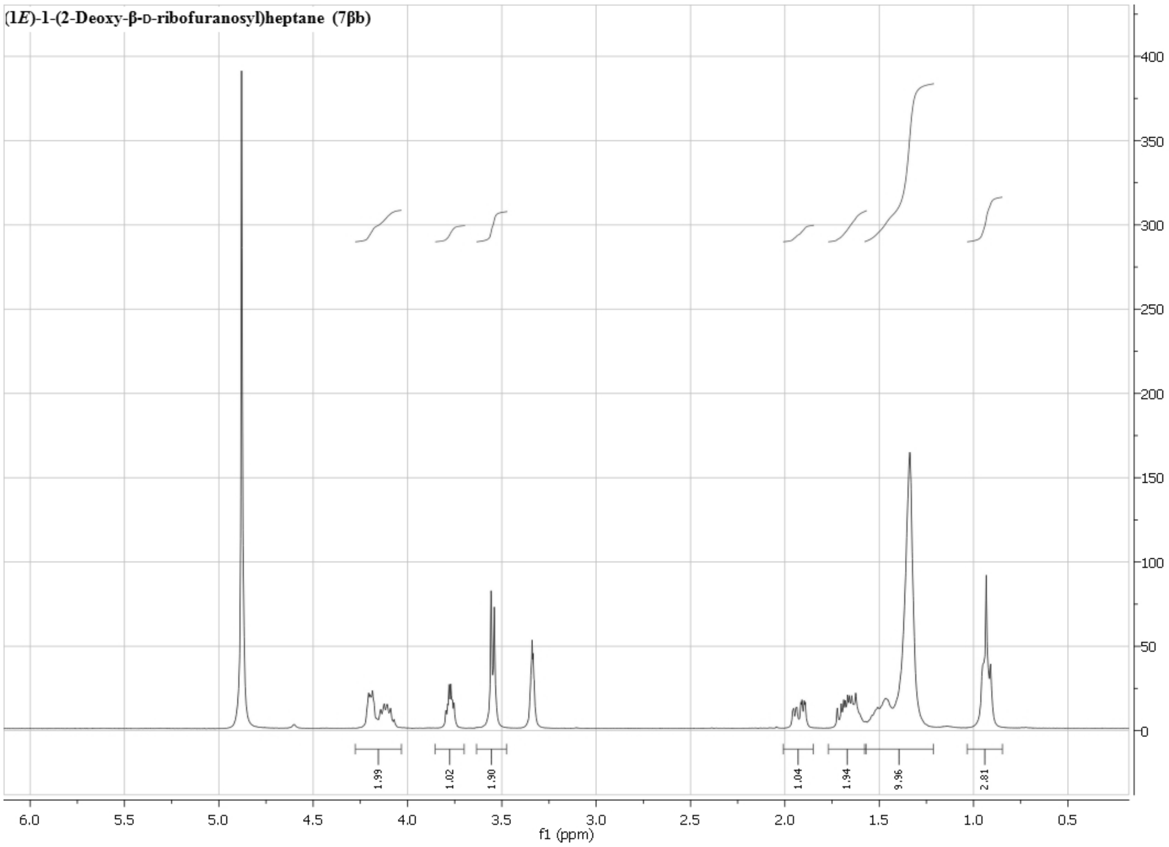

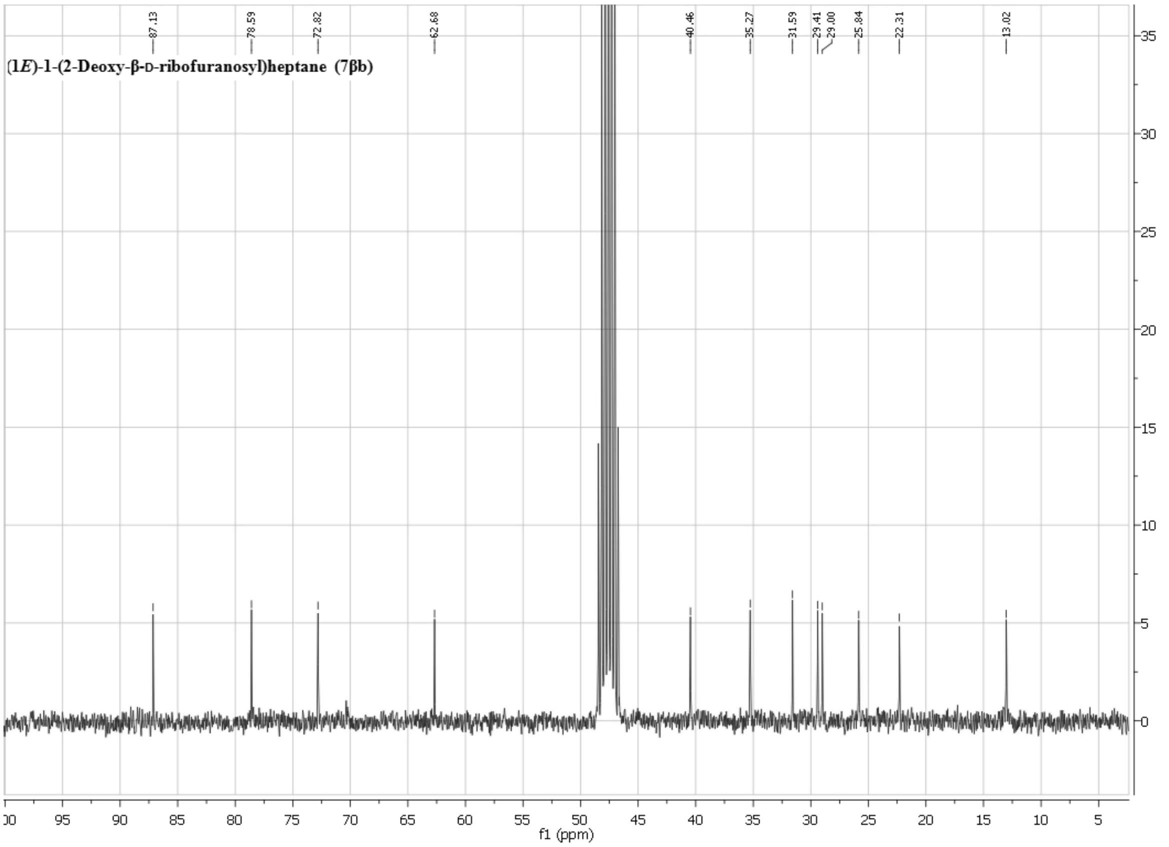

(1*E*)-1-(2´,3´,4´,6´-tetra-*O*-acetyl-α-D-galactopyranosyl)hept-1-ene (9b). ^{1}H NMR 500 MHz.

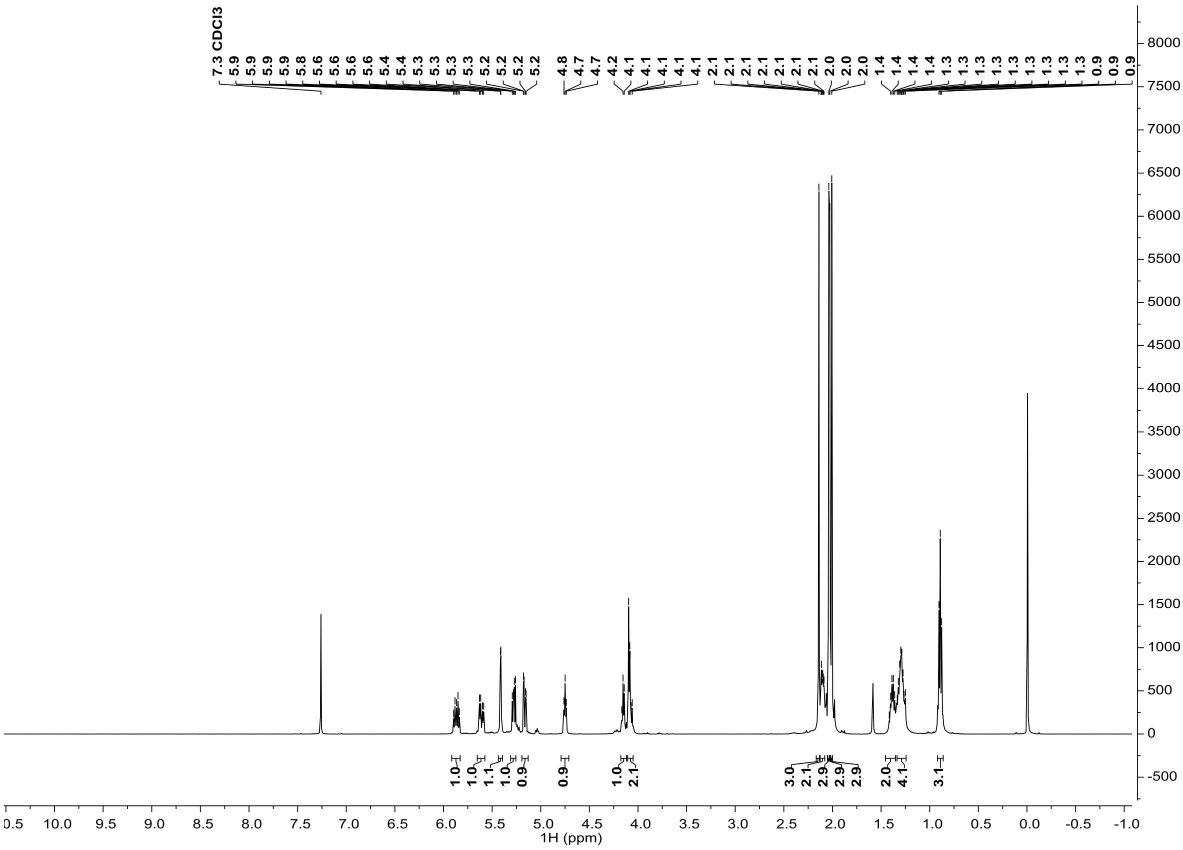

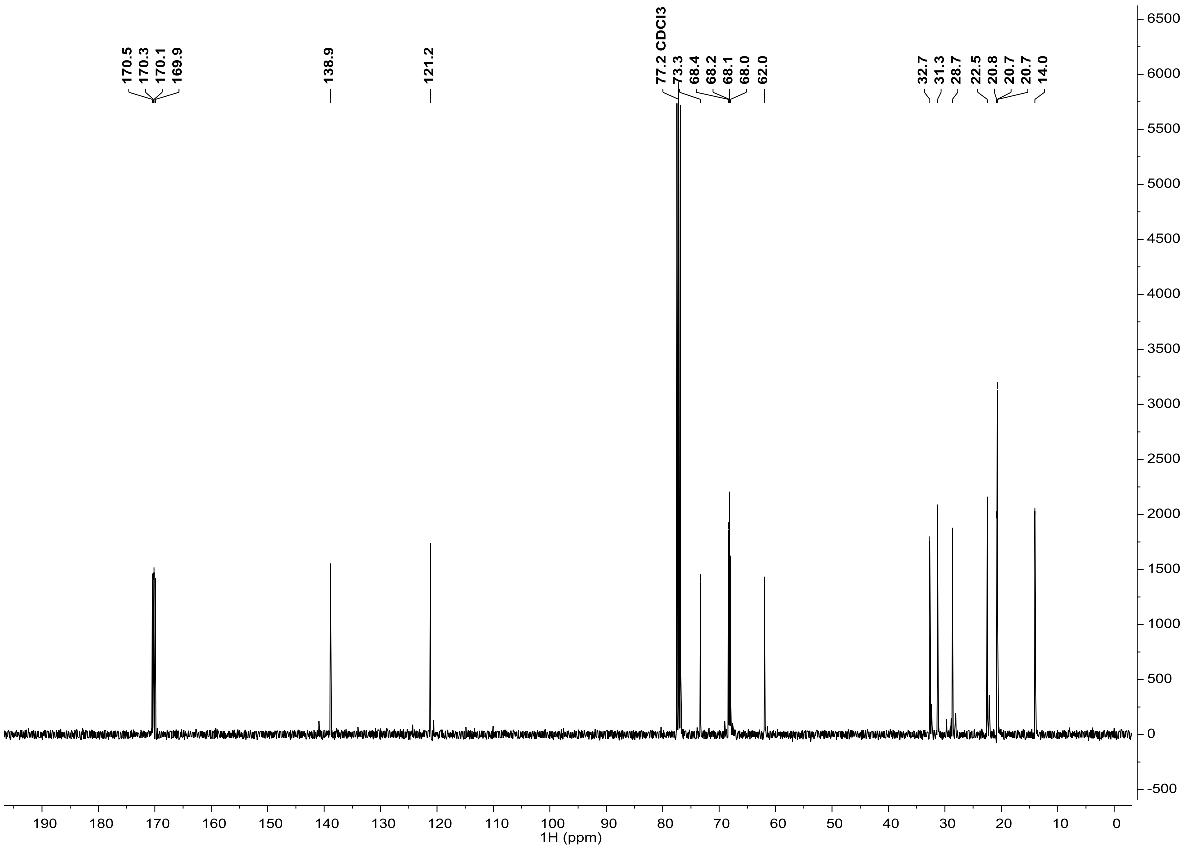

(1*E*)-1-(2´,3´,4´,6´-tetra-*O*-acetyl-α-D-galactopyranosyl)-4,4,5,5,6,6,7,7,8,8,9,9,9-tridecafluoronon-1-ene (9c). ^{1}H NMR (400 MHz, ^{13}C NMR 101 MHz, ^{19}F NMR 377 MHz in $CDCl_3$.

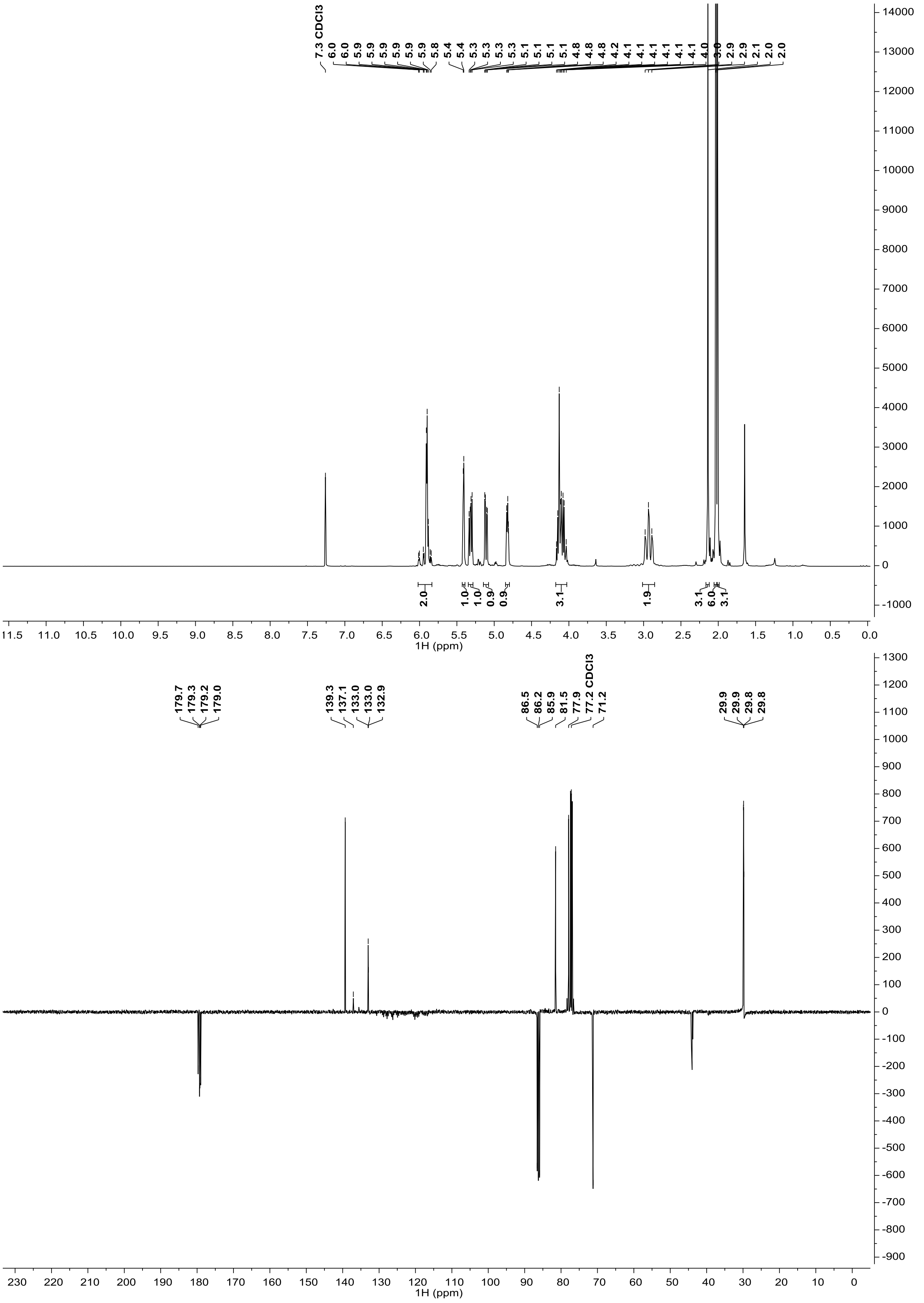

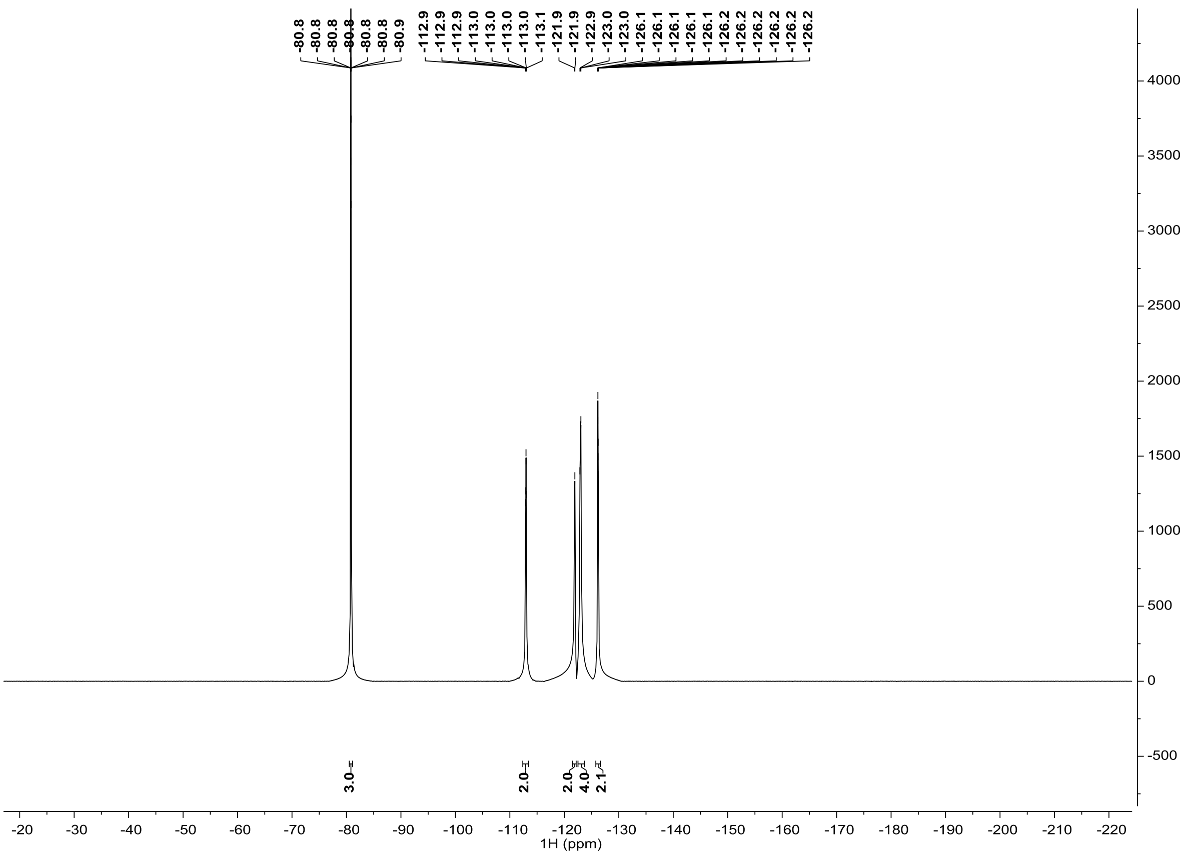
-80.8
-80.8
-80.8
-80.8
-80.8
-80.8
-80.9
-112.9
-112.9
-112.9
-113.0
-113.0
-113.0
-113.0
-113.1
-121.9
-121.9
-122.9
-123.0
-123.0
-126.1
-126.1
-126.1
-126.1
-126.1
-126.2
-126.2
-126.2
-126.2
-126.2
-126.2
3.0
2.0
2.0
4.0
2.1
1H (ppm)

(1*E*)-2-Phenyl-1-(2´,3´,4´,6´-tetra-*O*-acetyl-α-D-galactopyranosyl)ethene (9d). ^{1}H NMR 600 MHz, ^{13}C NMR 151 MHz in $CDCl_3$.

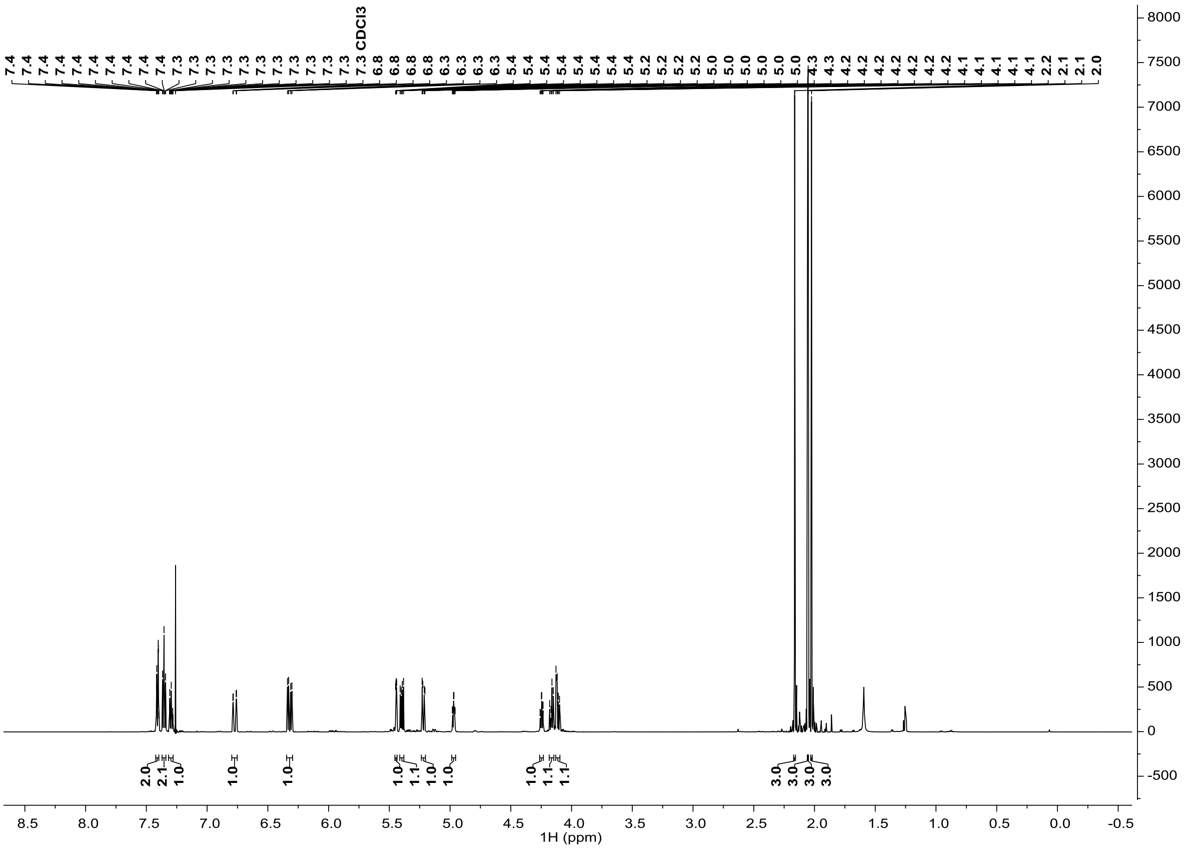

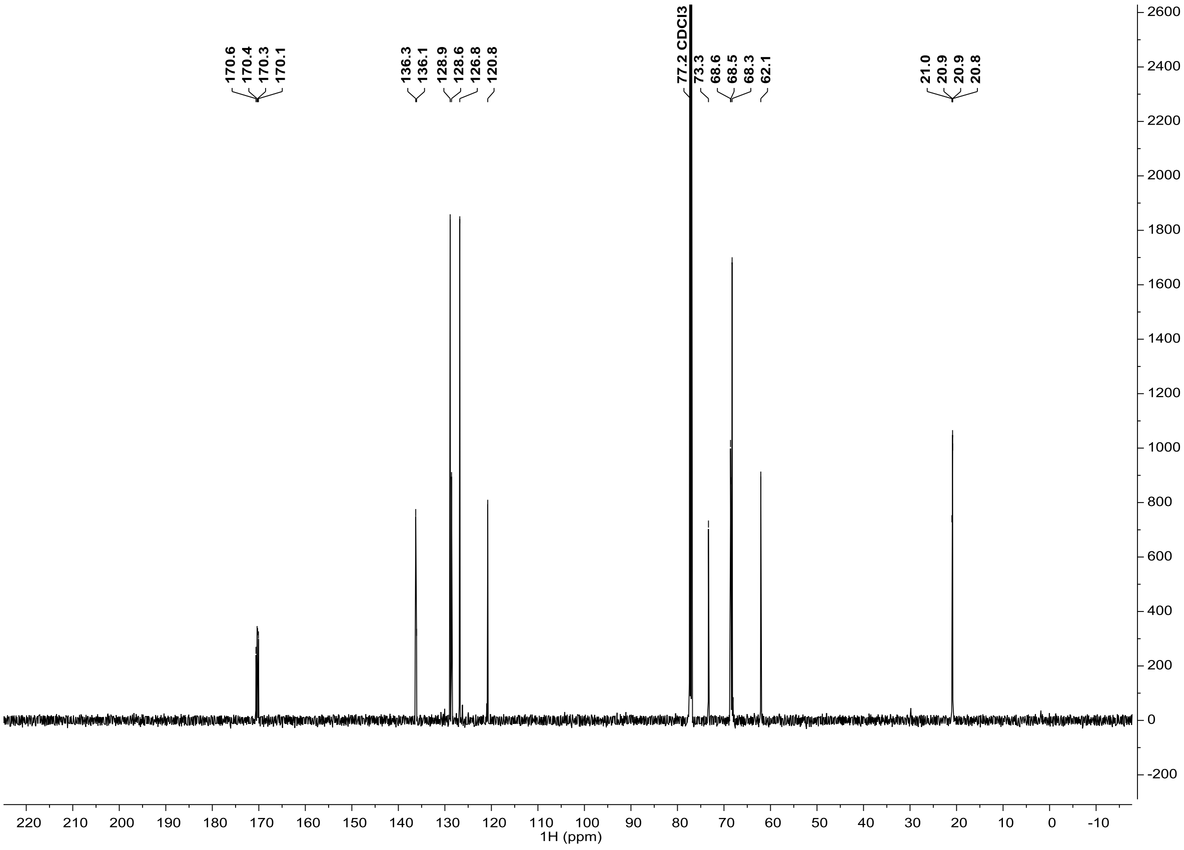

(1*E*)-2-(4´´-Fluorophenyl)-1-(2´,3´,4´,6´-tetra-*O*-acetyl-α-D-galactopyranosyl)ethene (9e).

^{1}H NMR 600 MHz, ^{13}C NMR 151 MHz, ^{19}F NMR 376 MHz in $CDCl_3$.

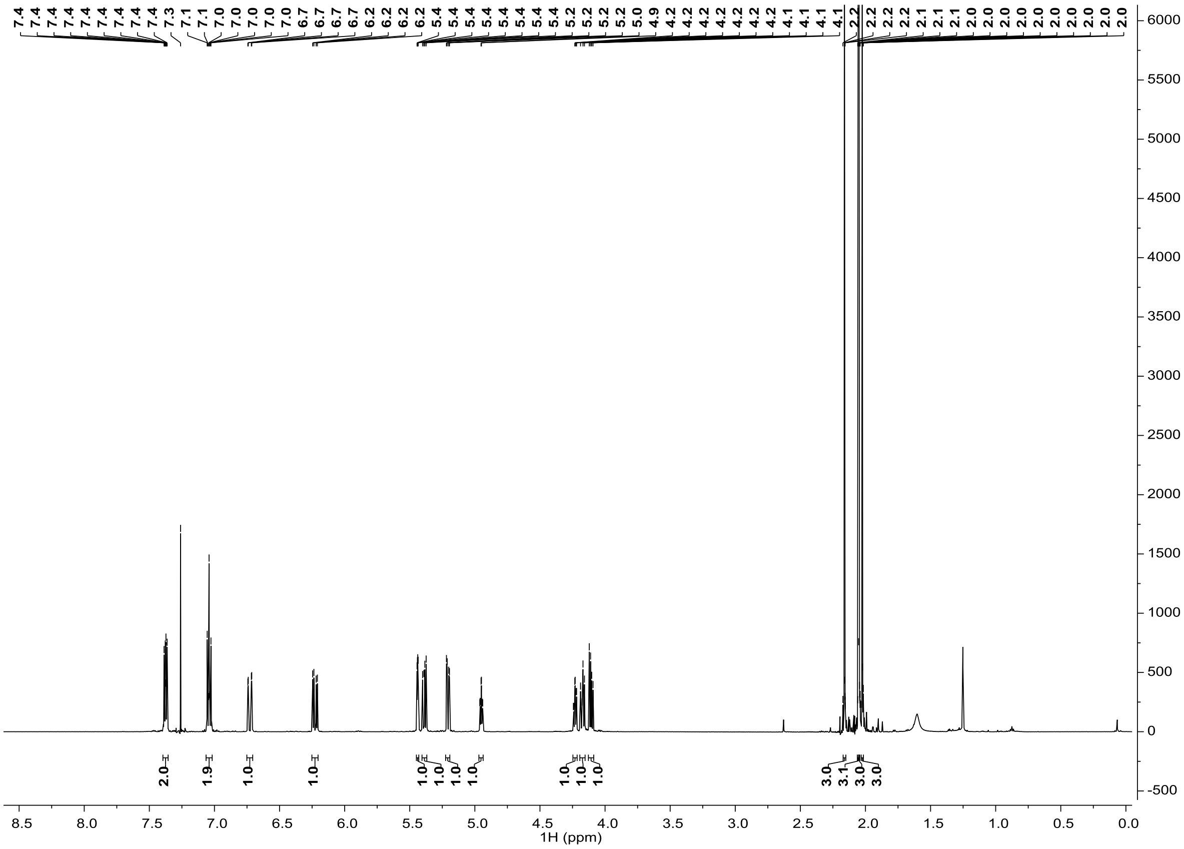

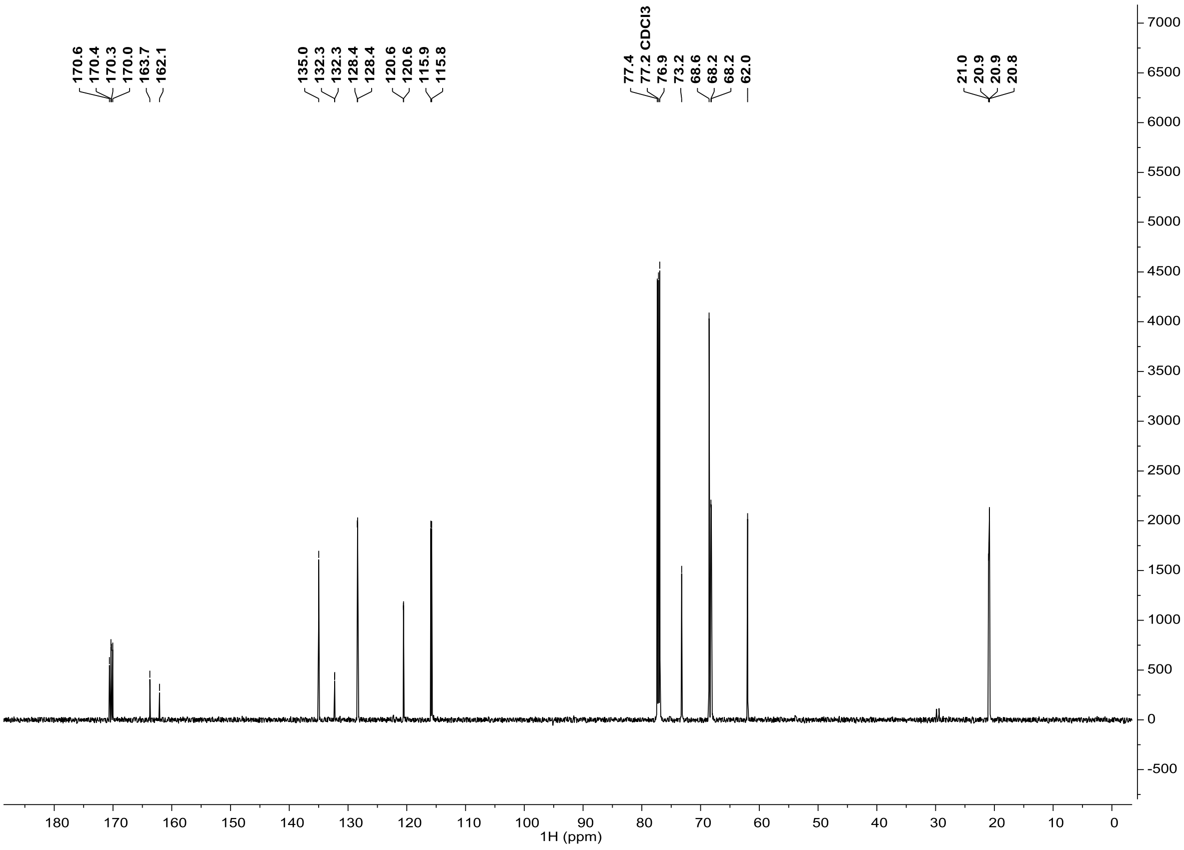

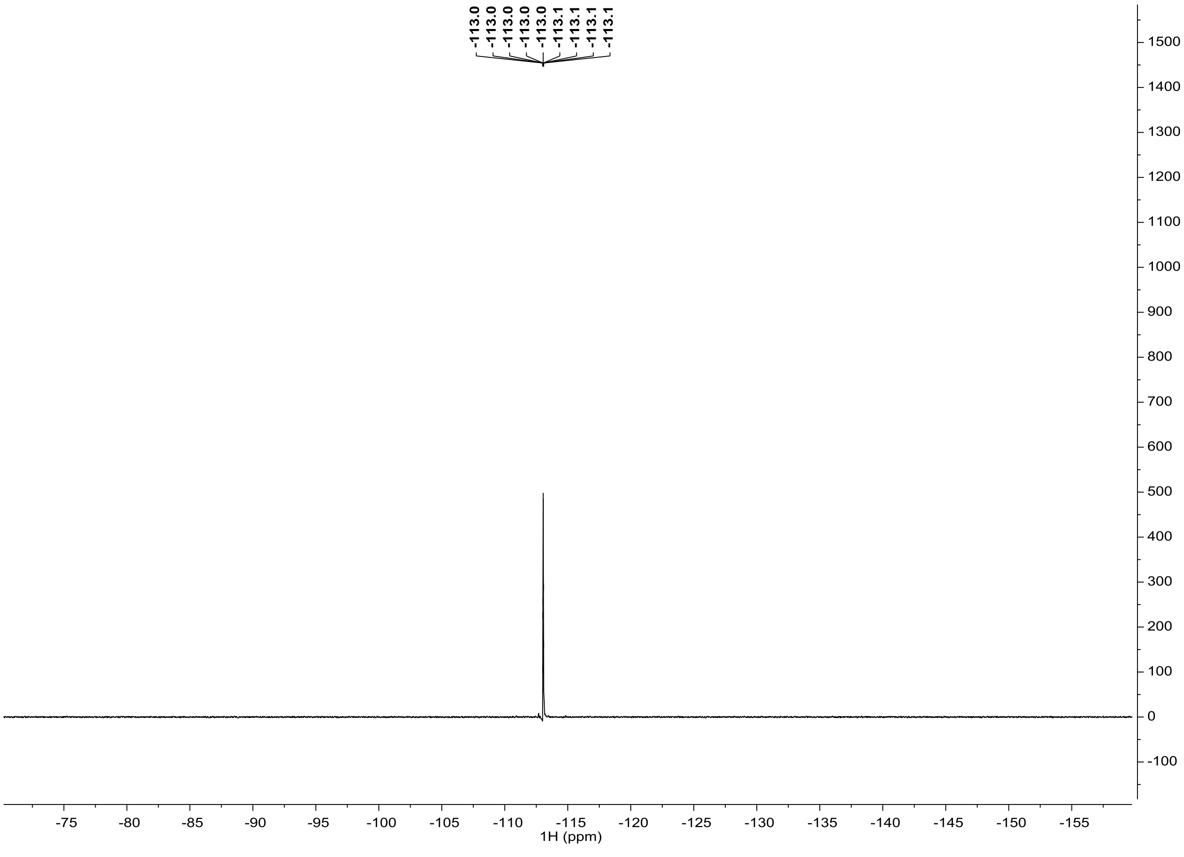
-113.0
-113.0
-113.0
-113.0
-113.0
-113.1
-113.1
-113.1
-113.1
1H (ppm)

(1*E*)-2-(4´´-Trifluoromethylphenyl)-1-(2´,3´,4´,6´-tetra-*O*-acetyl-α-D-galactopyranosyl)ethene (9f). ^{1}H NMR 600 MHz, ^{13}C NMR 151 MHz, ^{19}F NMR 376 MHz in $CDCl_3$.

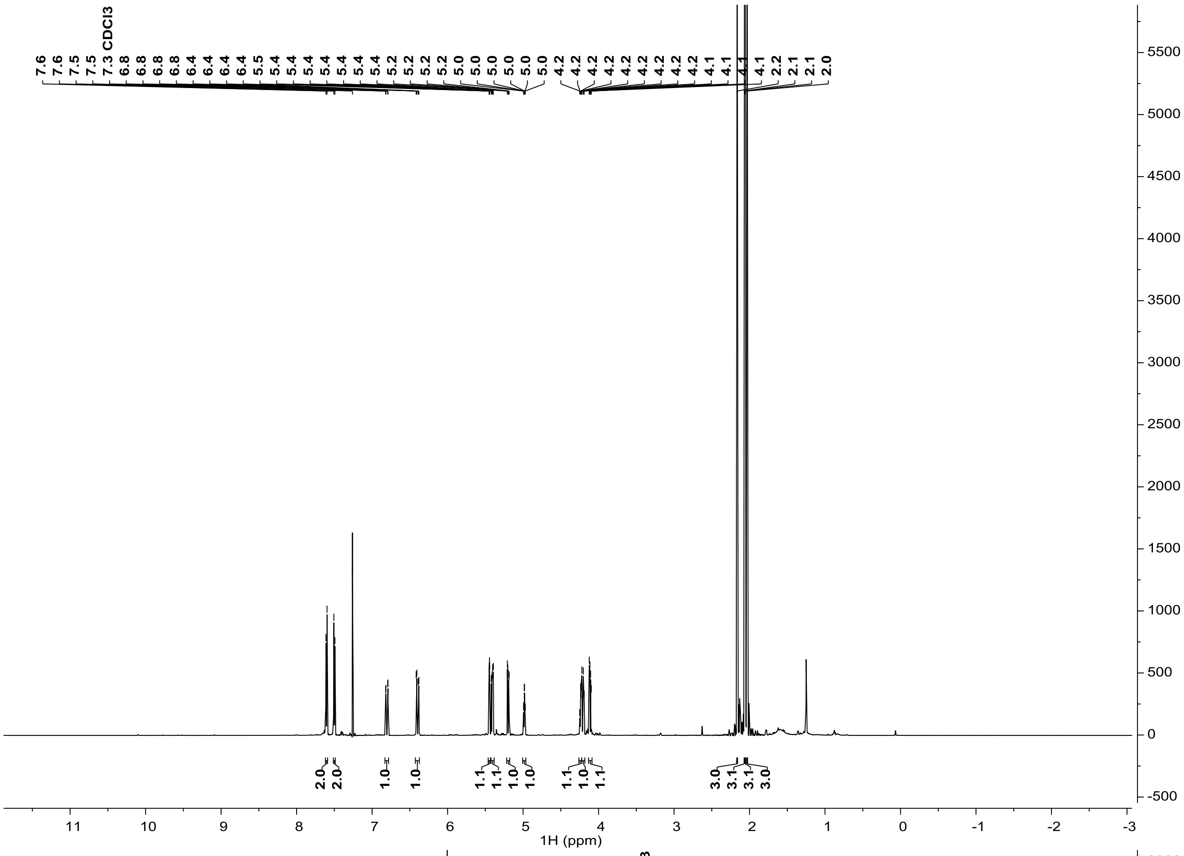

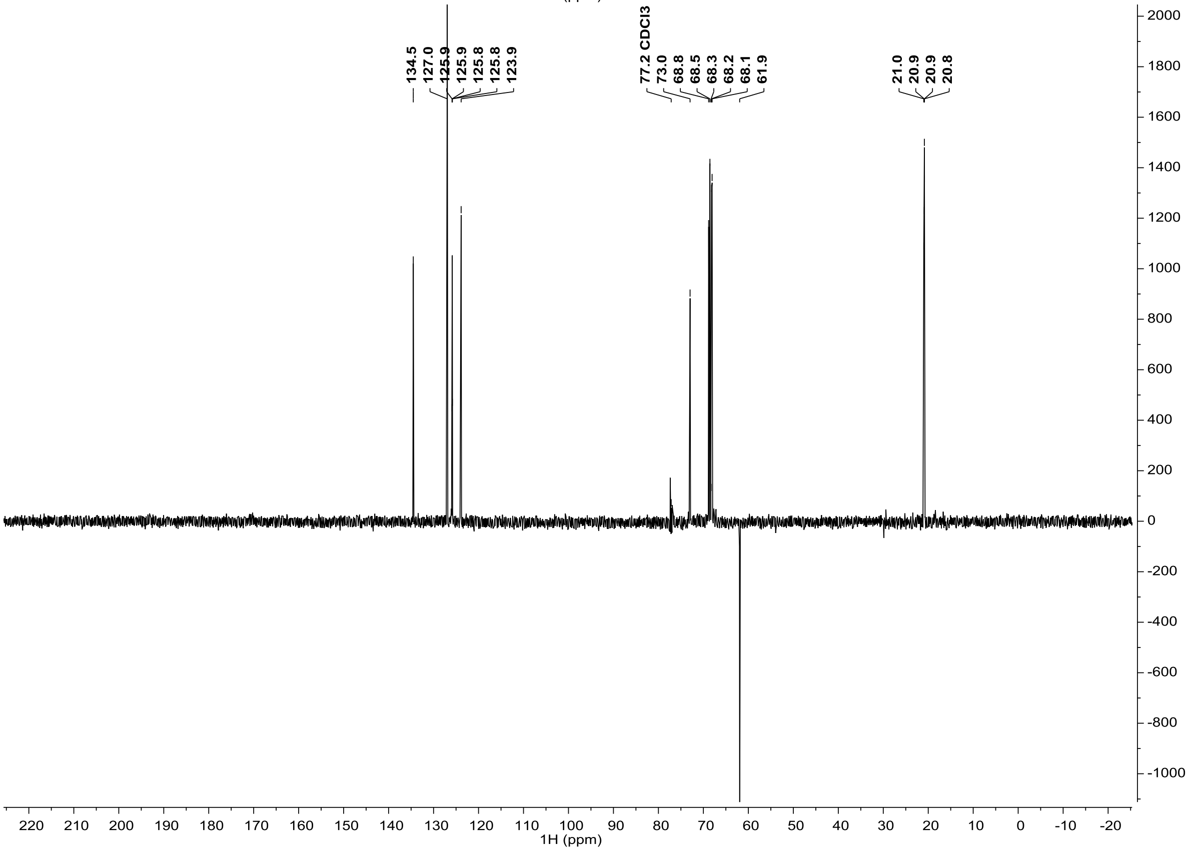

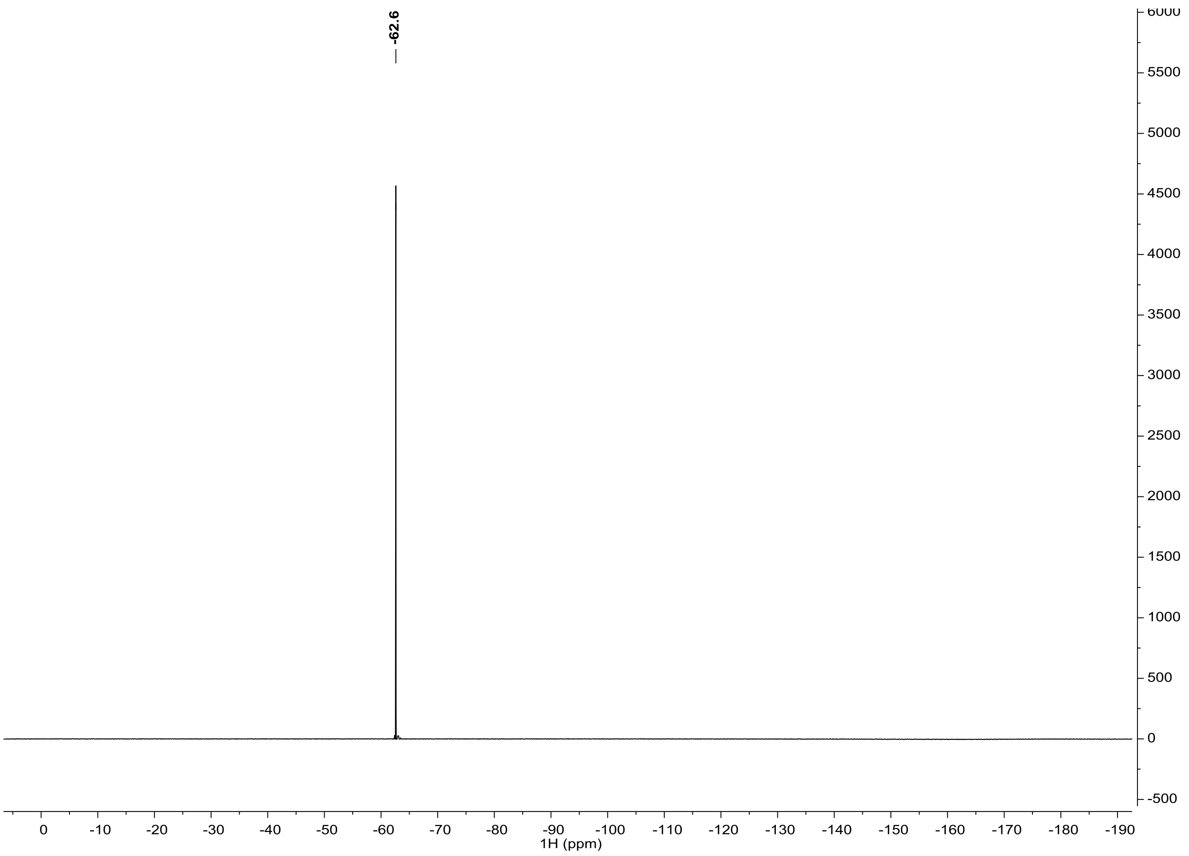
-62.6
6000
5500
5000
4500
4000
3500
3000
2500
2000
1500
1000
500
0
-500
0 -10 -20 -30 -40 -50 -60 -70 -80 -90 -100 -110 -120 -130 -140 -150 -160 -170 -180 -190
1H (ppm)

(1*E*)-1-(α-D-Galactopyranosyl)hept-1-ene (10b). ^{1}H NMR 400 MHz, ^{13}C NMR 101 MHz in MeOD.

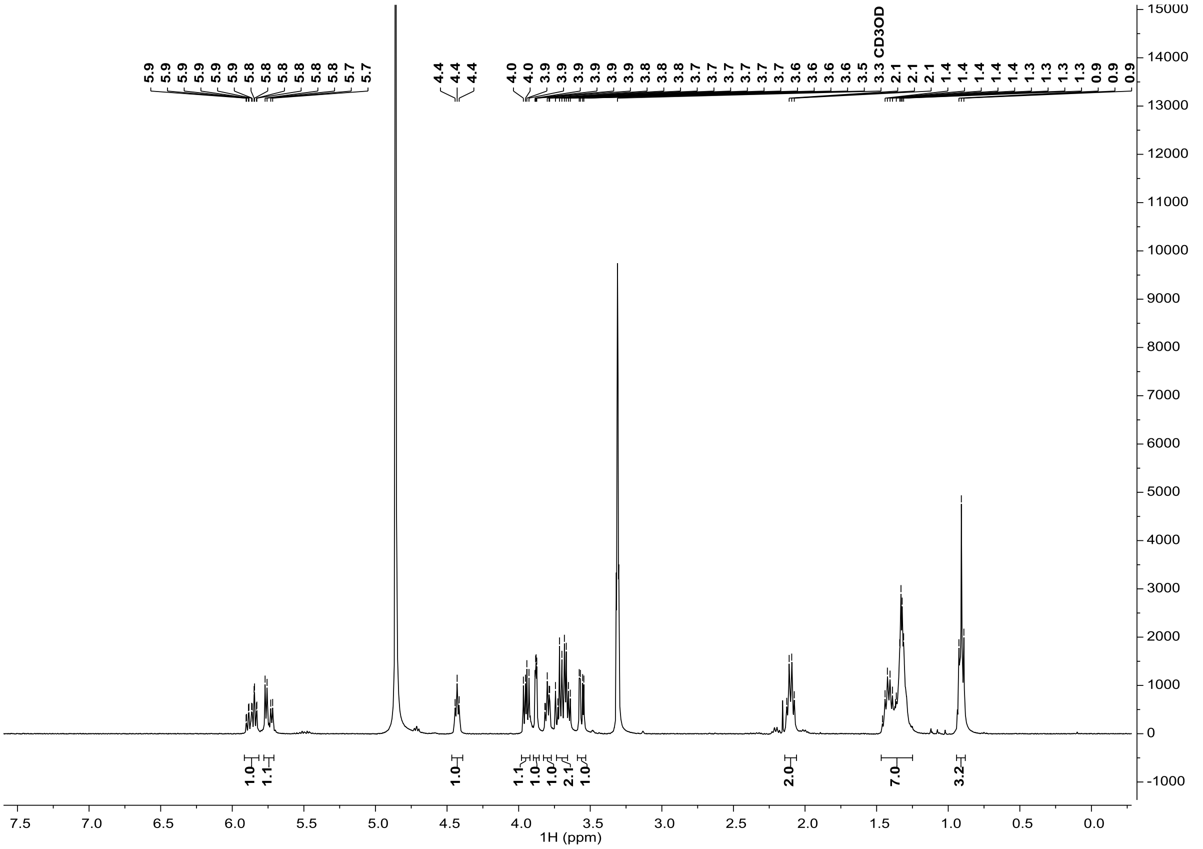

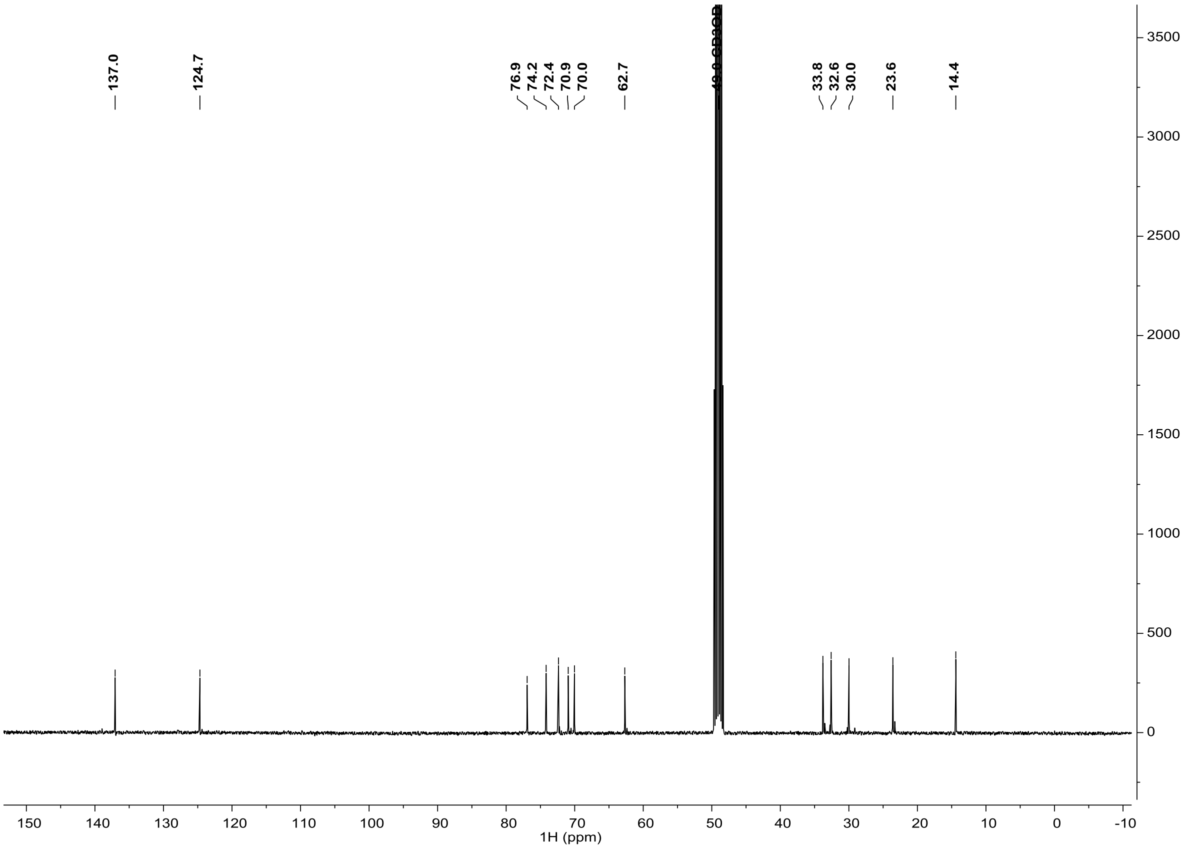

(1*E*)-1-(α-D-Galactopyranosyl)-4,4,5,5,6,6,7,7,8,8,9,9,9-tridecafluoronon-1-ene (10c). ^{1}H NMR 401 MHz, ^{13}C NMR 101 MHz, ^{19}F 377 MHz in MeOD.

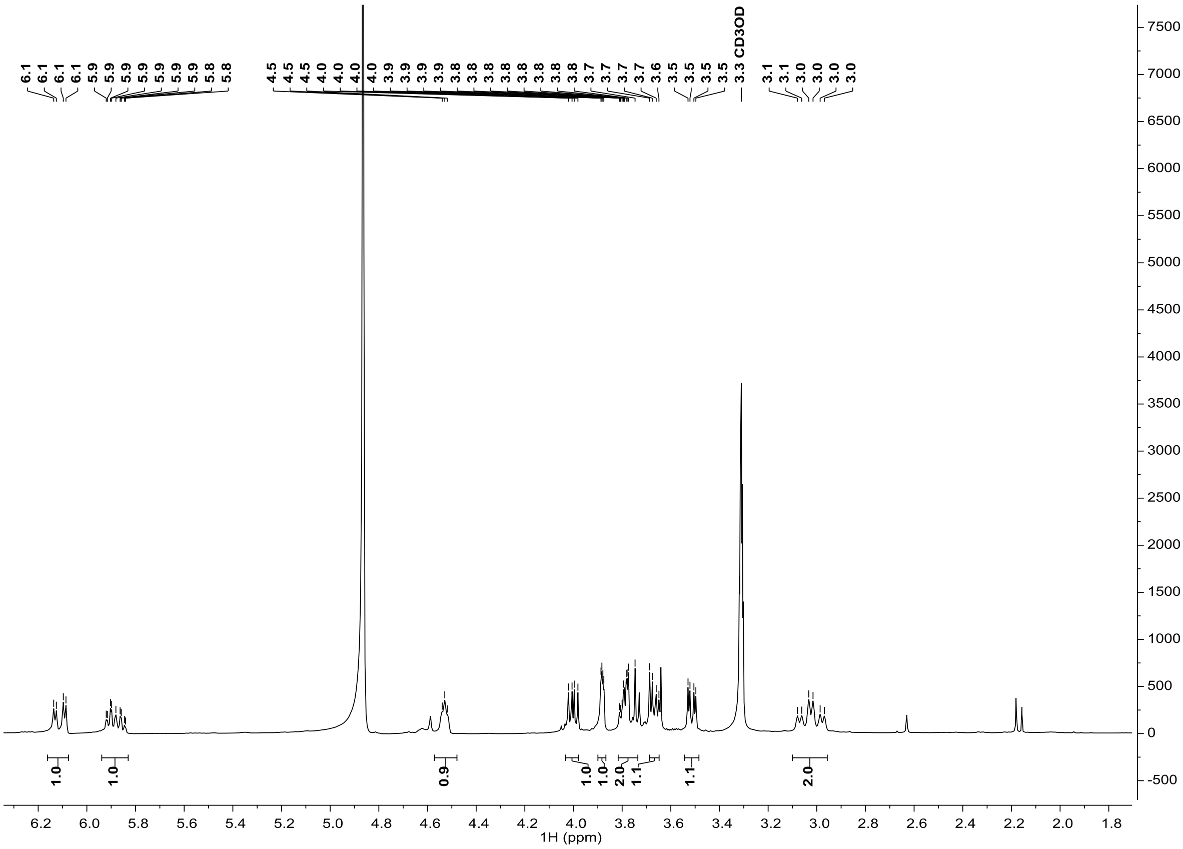

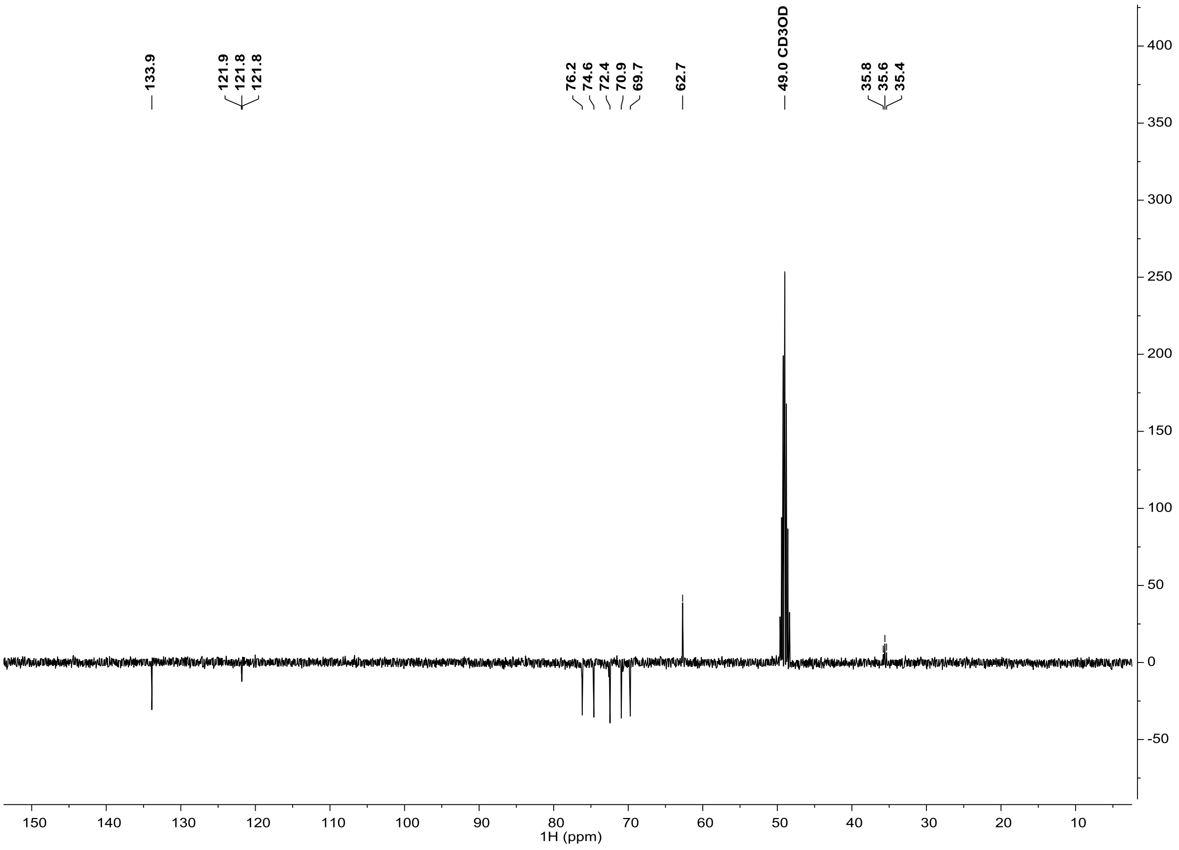

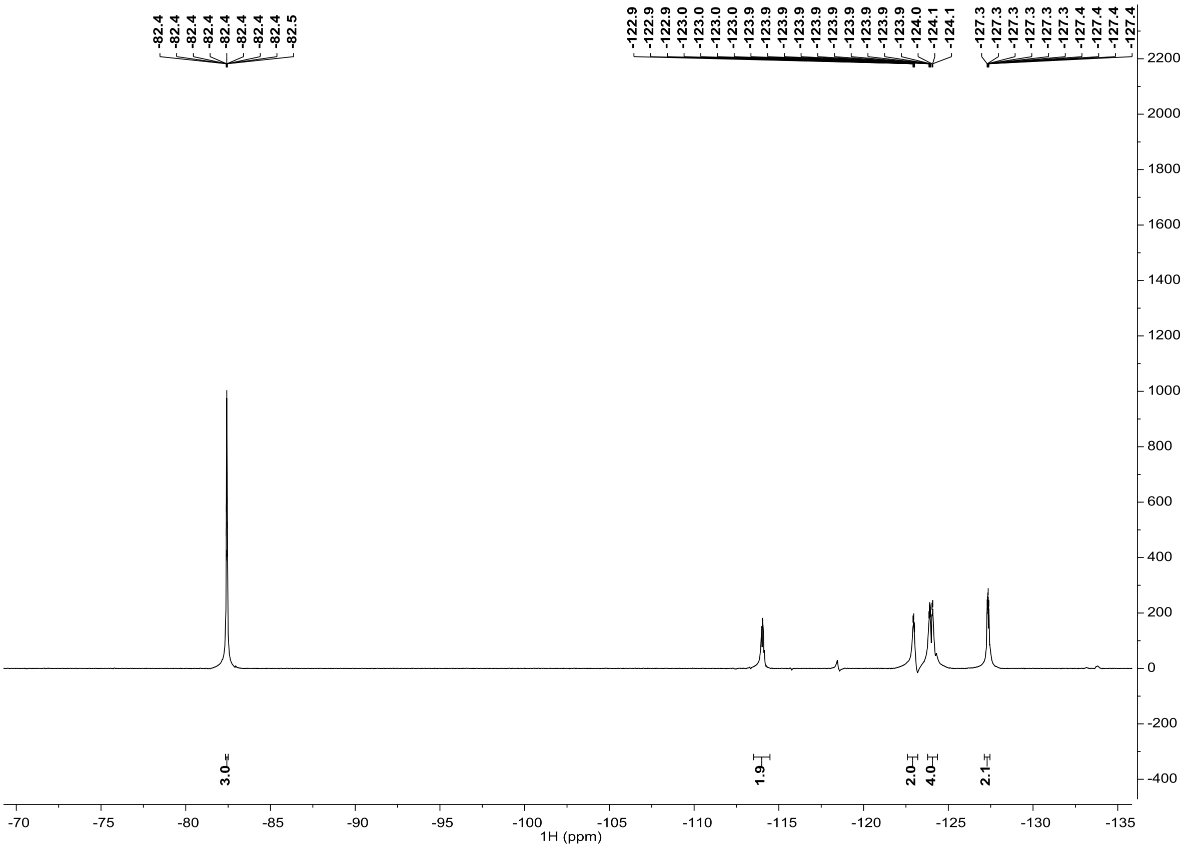

-82.4
-82.4
-82.4
-82.4
-82.4
-82.4
-82.4
-82.4
-82.5
-122.9
-122.9
-122.9
-123.0
-123.0
-123.0
-123.9
-123.9
-123.9
-123.9
-123.9
-123.9
-123.9
-123.9
-124.0
-124.1
-124.1
-127.3
-127.3
-127.3
-127.3
-127.3
-127.4
-127.4
-127.4
-127.4
3.0
1.9
2.0
4.0
2.1
1H (ppm)

(1*E*)-2-Phenyl-1-(α-D-galactopyranosyl)ethene (10d). ^{1}H NMR 600 MHz, ^{13}C NMR 150 MHz in MeOD.

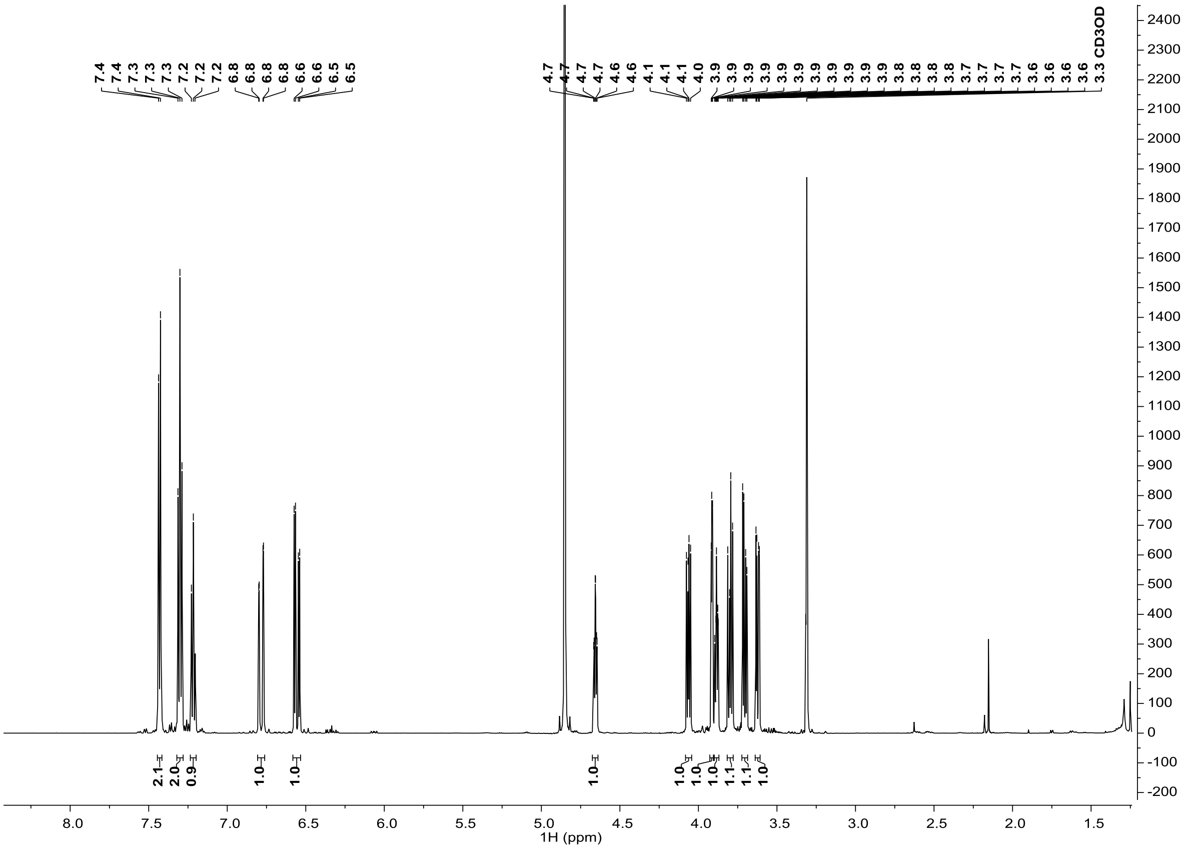

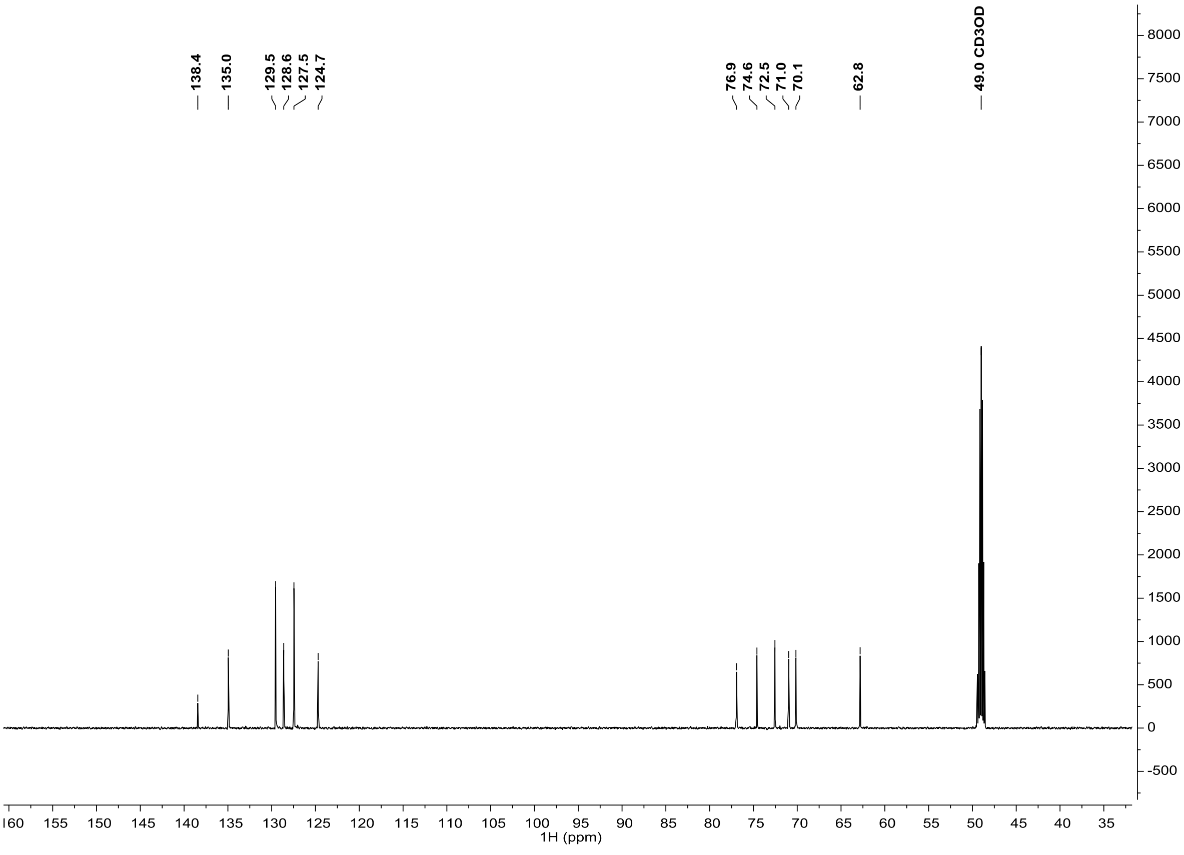

(1*E*)-2-(4´´-Fluorophenyl)-1-(α-D-galactopyranosyl)ethene (10e). ^{1}H NMR 600 MHz, ^{13}C NMR 151 MHz, ^{19}F 376 MHz in MeOD.

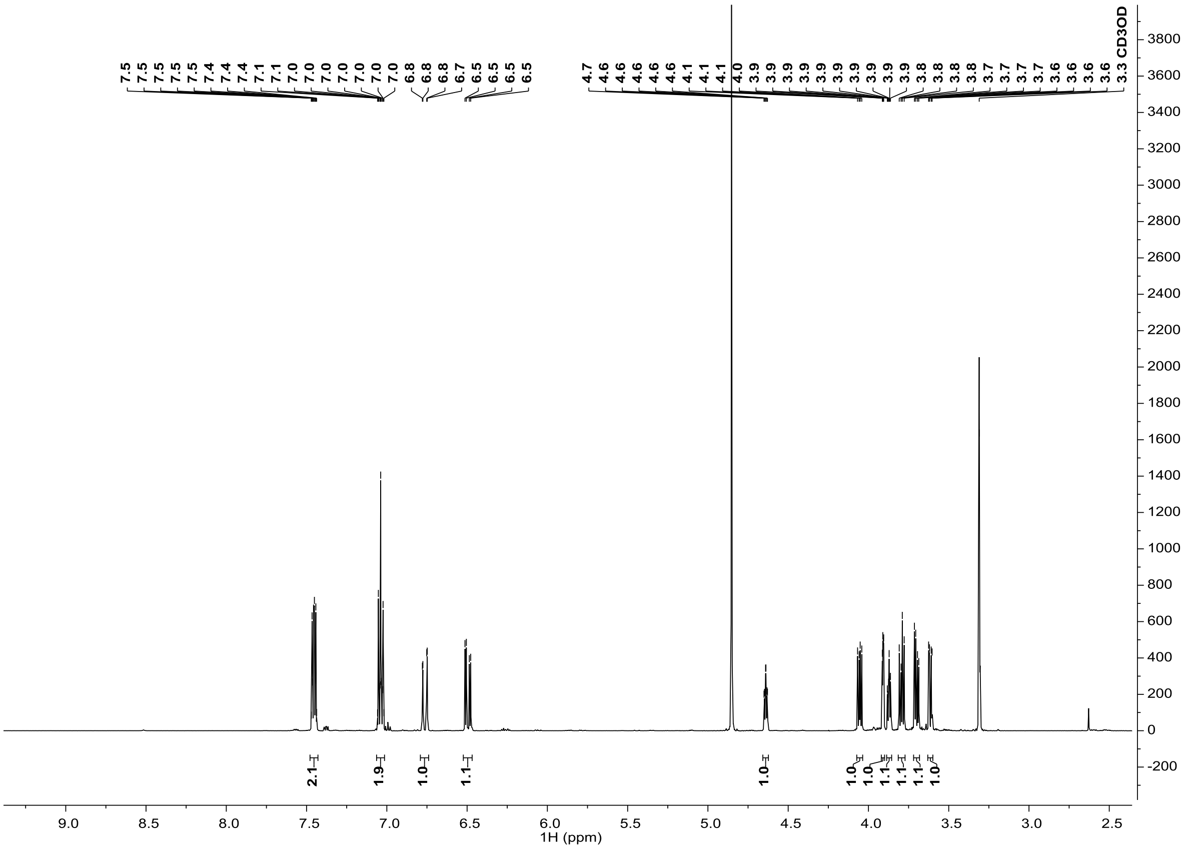

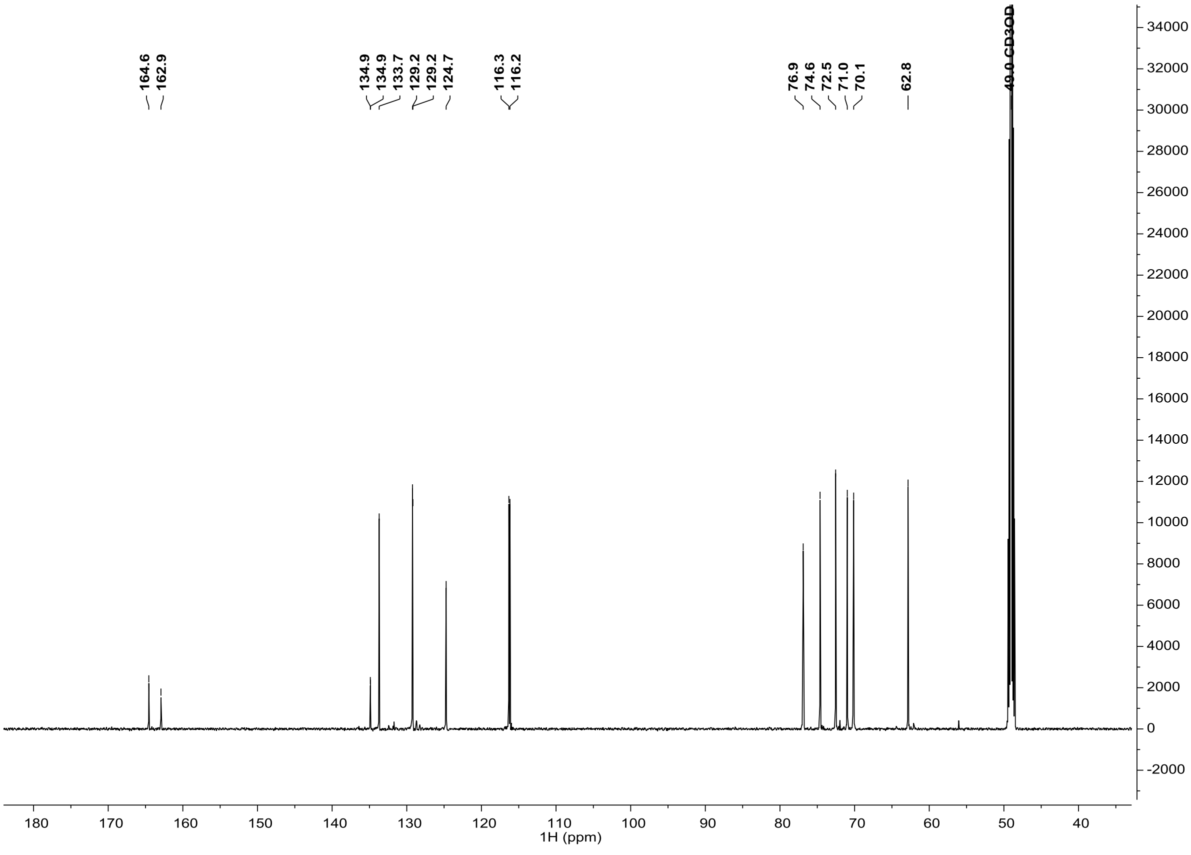

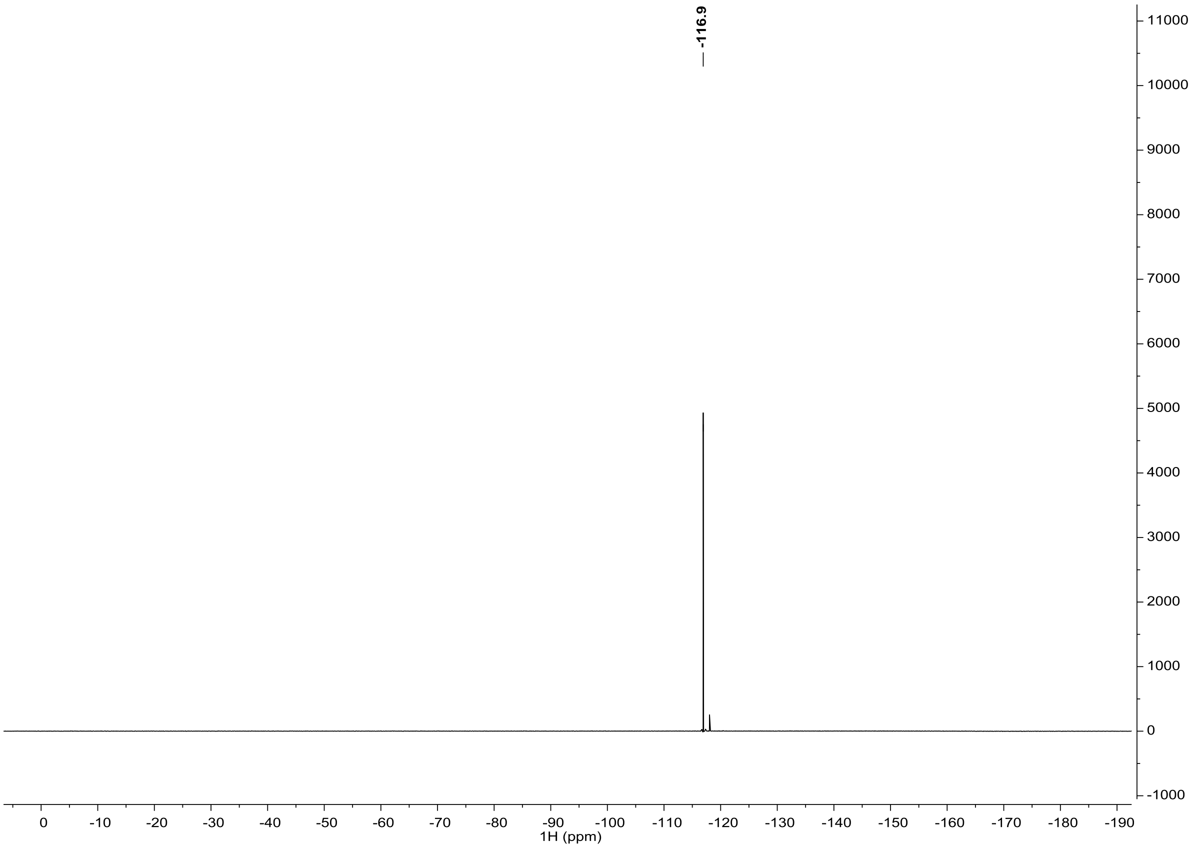
-116.9
1H (ppm)

(1*E*)-2-(4´´-Trifluoromethylphenyl)-1-(α-D-galactopyranosyl)ethene (10f). ^{1}H NMR 401 MHz, ^{13}C NMR 101 MHz, ^{19}F NMR 376 MHz in MeOD.

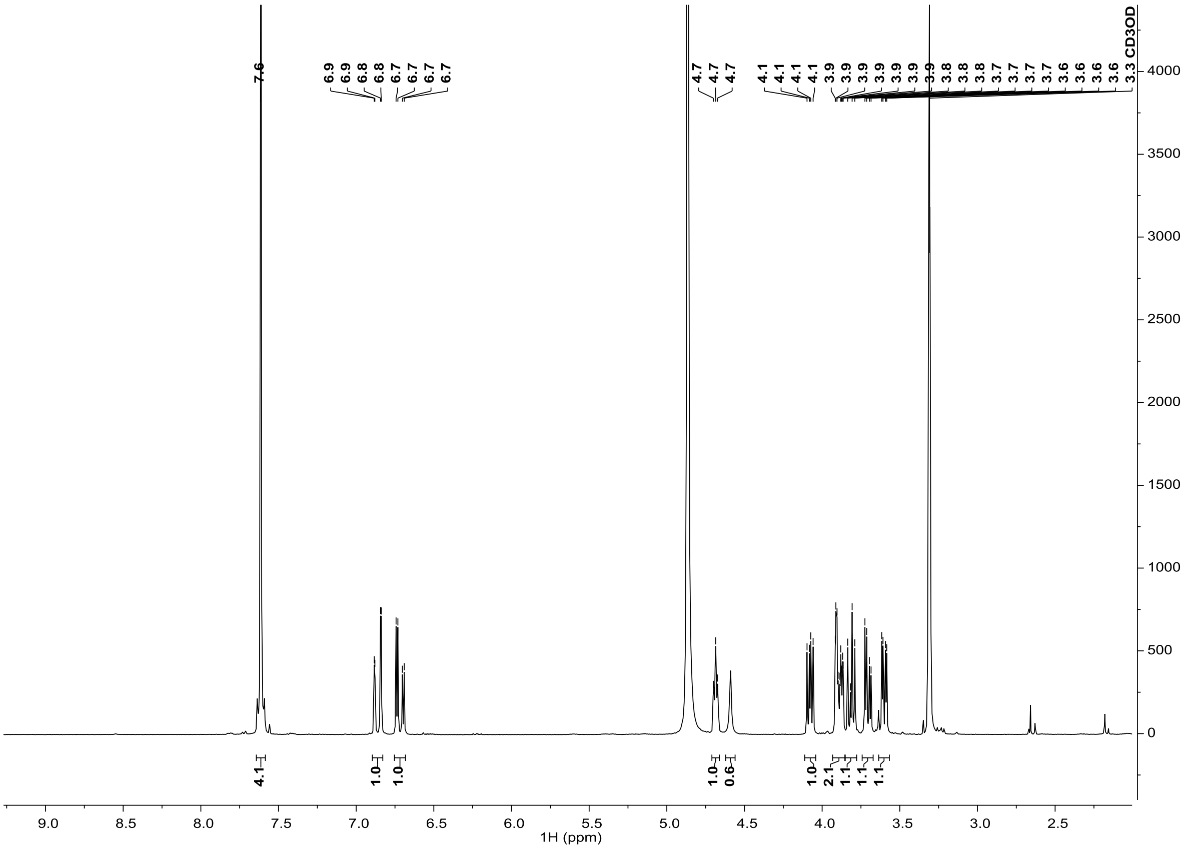

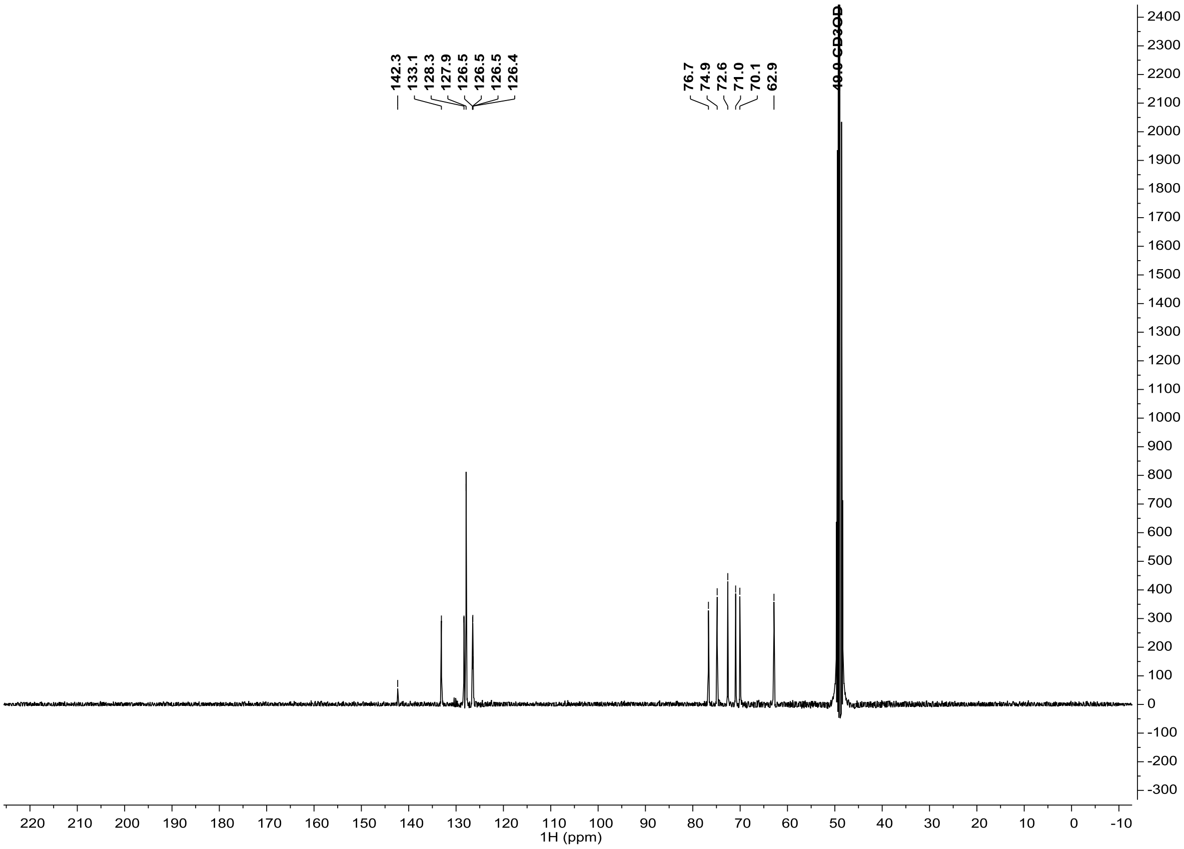

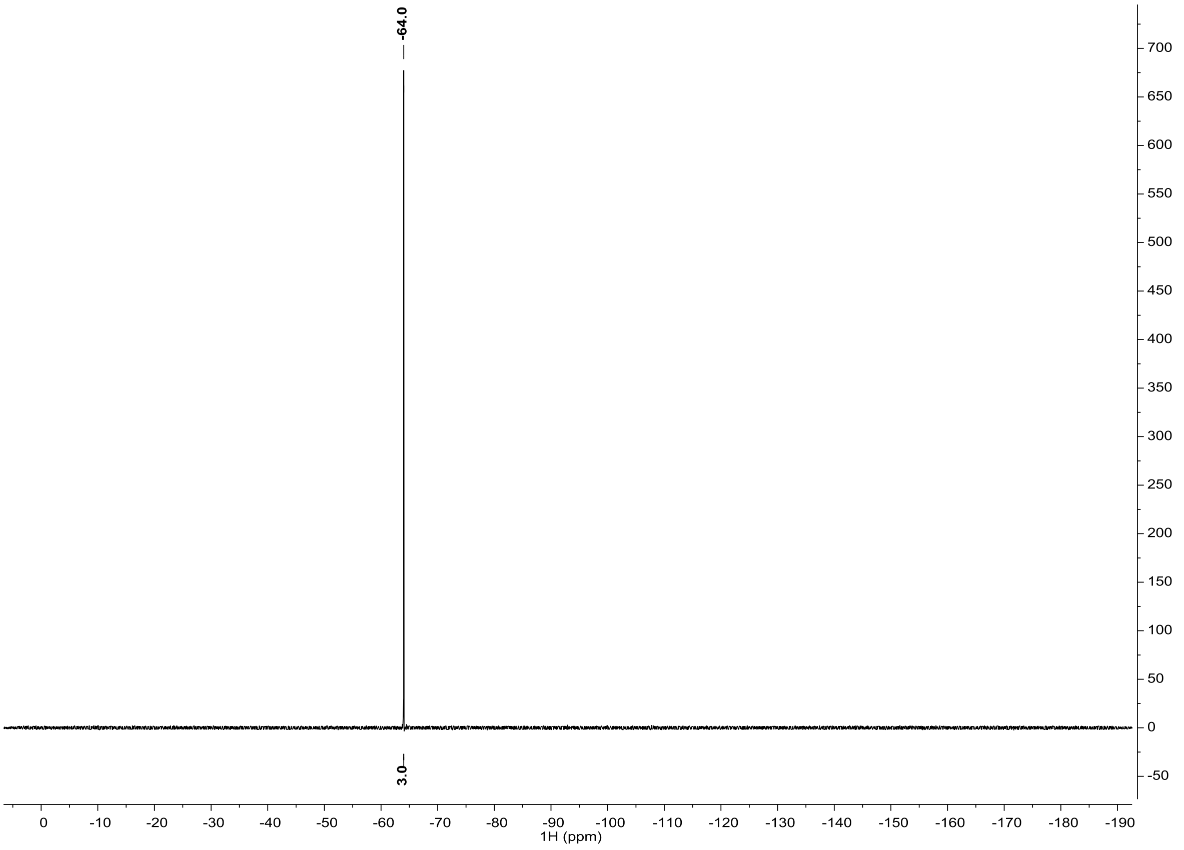

-64.0
3.0
700
650
600
550
500
450
400
350
300
250
200
150
100
50
0
-50
0
-10
-20
-30
-40
-50
-60
-70
-80
-90
-100
-110
-120
-130
-140
-150
-160
-170
-180
-190
1H (ppm)
